# Stress-Induced Secondary Metabolite Profiling in *Cistanche deserticola* Callus Cultures: Insights from GC-MS and HPLC-MS Analysis

**DOI:** 10.3390/ijms26136091

**Published:** 2025-06-25

**Authors:** Maxim Sutula, Nurtai Gubaidullin, Aizhan Rakhimzhanova, Shuga Manabayeva

**Affiliations:** 1 Plant Genetic Engineering Laboratory, National Center for Biotechnology, Astana 010000, Kazakhstan; m.sutula@biocenter.kz (M.S.); nur-tai.kz@mail.ru (N.G.); r.aizhann@mail.ru (A.R.); 2 Faculty of Natural Sciences, L.N. Gumilyov Eurasian National University, Astana 010000, Kazakhstan

**Keywords:** *C. deserticola*, callus culture, secondary metabolites, GC-MS, qTOF-UHPLC-MS, PhGs

## Abstract

Throughout human history, wild plant resources have played an invaluable role, serving as critical sources of food, medicine, and industrial materials. This study examined the callus cultures of *Cistanche deserticola* Y.C. Ma, a medicinal desert plant, by subjecting them to abiotic stress under controlled in vitro conditions. The secondary metabolite profiles were then analyzed using GC-MS and qTOF-UHPLC-MS. The GC-MS analysis revealed several bioactive compounds of pharmaceutical interest, such as γ-sitosterol and homovanillyl alcohol. PhGs, including echinacoside and salidroside, were quantified for the first time across 16 callus samples exposed to various stress treatments. The application of 0.1% Na_2_CO_3_ for 50 days resulted in the highest accumulation of echinacoside (13,378.9 µg/mL), and heavy metal stress notably increased salidroside levels to 27.0 µg/mL. There was a clear correlation between callus pigmentation and metabolic activity: orange and white calli produced significantly more PhGs than dark calli. These results suggest that *C. deserticola* callus cultures could be a sustainable, controllable platform for producing high-value secondary metabolites. This reinforces the importance of wild plant resources in modern science and industry.

## 1. Introduction

Wild plant resources have played an invaluable role throughout human history. Like many other countries, Kazakhstan is home to numerous plant species with medicinal properties [1]. These species hold significant potential for the development of the pharmaceutical industry. According to estimates, more than 1000 plant species in Kazakhstan have medicinal properties [2]. The officially approved list of medicinal plants in the Republic of Kazakhstan (Order No. 77, dated 7 March 2023, by the Ministry of Ecology and Natural Resources of the Republic of Kazakhstan) includes 278 species used in both official and traditional medicine. Among these species are those of the genus *Cistanche*. Secondary metabolites, which are naturally produced chemical compounds found in plants, are key contributors to their pharmacological activity [3,4]. These compounds serve various biological functions, including antioxidant activity. They are also used as dyes, flavor enhancers in food, cosmetic agents, insecticides, and therapeutic agents [5,6,7]. They participate in vital physiological processes, such as photosynthesis, nutrient assimilation, differentiation, translocation, protein synthesis, respiration, solute transport, and plant growth [8,9]. Unlike primary metabolites, such as amino acids, nucleotides, sugars, and lipids, which are ubiquitous in the plant kingdom, secondary metabolites are typically species- or genus-specific [10]. Several secondary metabolites found in natural extracts, including alkaloids, terpenoids, saponins, tannins, and flavonoids, exhibit notable antibacterial activity [11]. The number of known secondary metabolites is estimated to exceed 2,140,000, with approximately 4000 new compounds added annually [12]. This highlights the chemical diversity and therapeutic potential of plant-derived compounds.

Five species of the genus *Cistanche* Hoffmanns. & Link (Orobanchaceae Vent.) are native to Kazakhstan and are of particular interest in chemical and pharmacological research: *C. deserticola*, *C. salsa* (C.A. Mey.) Beck, *C. fissa* (C.A. Mey.) Beck, *C. flava* (C.A. Mey.) Korsh., and *C. tubulosa* (Schenk) Wight ex Hook.f. (Plants of the World Online). These perennial, herbaceous, parasitic plants inhabit the desert regions in Moiynkum, Betpak-Dala, and Mangystau. Since they lack chlorophyll [13], they have adopted a heterotrophic lifestyle and depend entirely on the roots of host plants, primarily *Tamarix* (Tamaricaceae) [14], *Haloxylon* [15], *Salsola* L. [16], *Anabasis* L. [17], *Kalidium* (*Chenopodiaceae*) [18], and *Calligonum* L. (*Polygonaceae*) [19]. Their thick stolons, which can reach up to 10 cm in diameter, are covered with alternating lanceolate scales. Flowering begins in March, and fruiting occurs from June to August. Reproduction occurs via seeds [13].

These plants have garnered considerable attention due to their rich chemical profiles and promising pharmacological properties [20,21]. Consequently, *Cistanche* species are widely used as raw materials for manufacturing pharmacologically active agents in many developed countries. Previous studies have examined species such as *C. salsa*, *C. deserticola*, and *C. tubulosa* for their bioactive and medicinal properties [22]. According to the official list of medicinal plants of the Republic of Kazakhstan, *C. salsa* is recognized for use in official medicine (https://adilet.zan.kz/rus/docs/V2300032038, accessed on 21 May 2025). The other four species are classified as valuable yet underutilized medicinal resources. They are not currently used in official medicine, though they are widely employed in traditional practices in China, Korea, Japan, and Kazakhstan. Numerous studies have documented their pharmacological activities [23,24].

However, growing populations, urbanization, climate change, and unregulated harvesting from the wild endanger these valuable species. Therefore, developing and applying in vitro cultivation techniques, including micropropagation, is essential to conserving and sustainably using these species. Advanced propagation protocols now effectively meet pharmaceutical demands [25]. Micropropagation is widely used to produce bioactive compounds in medicinal plants [26,27], including rare and endangered species [28,29].

*Cistanche* species are particularly noteworthy for their ability to accumulate phenylethanoid glycosides (PhGs), which are natural compounds of high pharmacological value [30,31]. The biosynthesis and accumulation of PhGs are governed by complex biochemical pathways influenced by tissue type, environmental conditions, and stress factors [32]. Manipulating the growth medium composition or applying specific stressors can increase the production of secondary metabolites, underscoring the importance of sensitive analytical methods for studying these compounds in under-explored tissues such as callus cultures [33,34].

Gas chromatography (GC) and high-performance liquid chromatography (HPLC), particularly when coupled with mass spectrometry (MS), are powerful analytical tools for the identification and quantification of plant secondary metabolites. These techniques are widely used in pharmaceutical, ecological, food science, and forensic research [35,36,37,38,39,40,41]. GC-MS and HPLC-MS have been effectively used to analyze *Cistanche* stolons, revealing a variety of 49 compounds, including essential oils, 17 PhGs, 10 iridoids (e.g., cistanin and cistachlorin), D-mannitol, succinic acid, and steroids (β-D-sitosterol, β-D-sitosterol glucoside). Phenolic compounds include salidroside [42,43,44,45], echinacoside [46,47,48,49], tubuloside [50], verbascoside (syn.: acteoside) [51,52,53], acetylacteoside [54,55,56], cistanoside, osmanthuside, dicaffeoylacteoside, lignans (liriodendrin, O-β-D-glucopyranoside, syringin, and pinoresinol), and flavonoids [57,58]. A GC-MS analysis of essential oils extracted from stolons identified 24 volatile compounds, including aldehydes, phenols, alcohols, and ketones. The dominant component was eugenol [58,59]. Despite extensive research on PhGs in vegetative organs, studies on their occurrence and accumulation in Cistanche callus cultures are extremely limited and inadequately documented. Currently, there are no published data on the presence of PhGs in Cistanche callus cultures [60,61].

Strategies that promote the biosynthesis of valuable bioactive compounds (BACs) are of considerable interest, particularly those involving elicitor-based treatments and the application of abiotic stress factors in plant cell and tissue cultures. Elicitors, whether biological, chemical, or environmental stress agents, can activate specific metabolic pathways, significantly enhancing the production of PhGs and other bioactive molecules [62,63,64,65,66]. Applying abiotic stress factors to the callus culture of *C. deserticola* may stimulate the accumulation of secondary metabolites, the central focus of our study.

In this study, we analyzed the chemical profiles of volatile, semi-volatile, and soluble secondary metabolites using established *C. deserticola* callus cultures. We characterized their structural diversity using GC-MS and qTOF-UHPLC-MS. Studying the biosynthesis of BACs in promising medicinal plants cultivated in vitro is important for conserving endangered species and developing a stable, controllable source of phytochemicals for pharmaceutical production, especially given climate change and growing global demands.

## 2. Results

### 2.1. Identification of Secondary Metabolites in C. deserticola Callus Tissues by GC-MS

**Figure 1:**
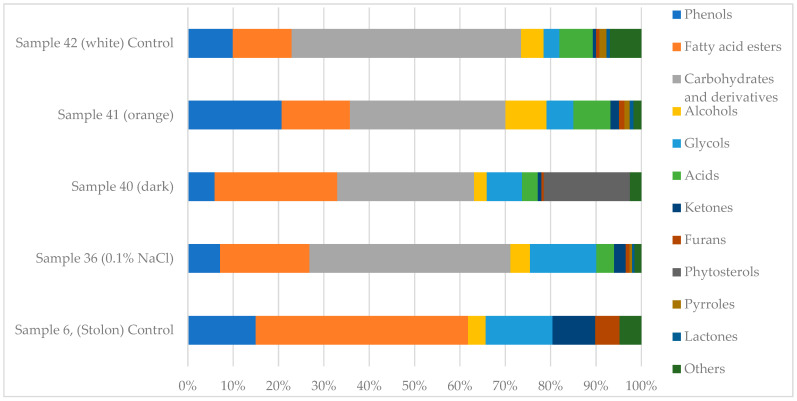
Metabolic spectra of biologically active compounds in *C. deserticola* callus samples identified by GC-MS analysis.

A comparative GC-MS analysis was performed to characterize the profiles of volatile and semi-volatile compounds in *C. deserticola* tissues (stolons; sample 6), callus tissues cultivated under moderate salt stress (sample 36), and callus tissues grown under standard culture conditions (samples 40, 41, and 42). The identified compounds and their relative abundances are summarized in Table 1. Complete chromatographic data and spectral outputs are provided in Supplementary Materials (Tables S1–S5). The qualitative composition and relative abundance of bioactive metabolites varied significantly among callus samples, as illustrated in the GC-MS-based metabolic spectra (Figure 1). The primary GC-MS data on volatile and semi-volatile secondary metabolites in *C. deserticola* callus tissues were obtained and partially characterized in our previous research [67], providing a foundation for the further investigation of compound profiles under varying cultivation conditions.

Overall, GC-MS analysis revealed a diverse range of secondary metabolites, with significant variations in compound composition and relative abundance among the analyzed tissue types. Several core metabolites, such as phenolic compounds, fatty acid methyl esters, and glycols, were common across samples, but their concentrations differed significantly. Most of the identified compounds are secondary plant metabolites known for their potential biological activity (see Supplementary Materials, Table S6).

The stolon tissue (sample 6) exhibited a high concentration of diethylene glycol monododecyl ether (13.2%), tetraethylene glycol (11.5%), methyl palmitate (11.8%), and ethanone, 1-(2-hydroxy-5-methylphenyl) (9.4%). Phenolic derivatives were also prevalent, including 2,4-bis(1,1-dimethylethyl)-phenol (8.3%) and 1,2-dimethoxy-4-ethenylbenzene (6.7%).

In contrast, callus samples 36–42 exhibited elevated concentrations of ethyl α-D-glucopyranoside, particularly sample 36 at 44.3%. They also exhibited moderate levels of methyl palmitate (4.26%), tetraethylene glycol (3.79%), and diethylene glycol monododecyl ether (0.88%). Sample 40 had a distinct metabolite profile containing 24.94% ethyl α-D-glucopyranoside, 19.01% γ-sitosterol, and 9.37% 1,4-benzenedicarboxylic acid, bis(2-ethylhexyl) ester. Sample 41 was dominated by ethyl α-D-glucopyranoside (23.71%), β-D-glucopyranose, 1,6-anhydro- (8.15%), and homovanillyl alcohol (3.91%). Sample 42 showed 41.78% ethyl α-D-glucopyranoside, 8.23% β-D-glucopyranose 1,6-anhydro, and 2.80% homovanillyl alcohol.

Fatty acid esters and phenolic compounds were consistently detected in all samples, though they were generally more abundant in stolon tissue (sample 6). For example, hexadecanoic acid, methyl ester was identified in all samples, with concentrations ranging from 4.26% in callus tissue to 11.8% in stolons. Glycol derivatives, such as diethylene glycol monododecyl ether and tetraethylene glycol, were also prevalent.

Samples 36 and 40 demonstrated notably elevated levels of ethyl α-D-glucopyranoside, a compound that may be involved in carbohydrate metabolism and glycoside biosynthesis. Additionally, samples 40 and 42 showed significant amounts of γ-sitosterol and β-D-glucopyranose, which are bioactive compounds with applications in pharmaceutical and cosmetic formulations.

### 2.2. Identification and Quantification of PhGs by qTOF UHPLC-MS

The quantitative profiling of PhG content in *C. deserticola* callus cultures subjected to various stress conditions revealed significant variation in PhG accumulation depending on external factors as well as callus color (Table 2).

Sample 38, consisting of orange calli treated with 0.1% Na_2_CO_3_ for 50 days, exhibited the highest concentration of echinacoside, reaching 13,378.9 µg/mL. Other samples exhibited notably elevated levels of echinacoside under specific stress conditions. Sample 17, which consisted of white calli treated with 0.1% Na_2_CO_3_ for 20 days, accumulated 10,937.6 µg/mL of echinacoside. Similarly, white calli exposed to 0.1% NaCl for 20 (sample 15) or 50 (sample 37) days produced 10,175.1 and 10,615.4 µg/mL of echinacoside, respectively (Table 2).

These values significantly exceeded those of the untreated control samples (samples 40–42), which had echinacoside concentrations of 408.7, 8102.0, and 5499.0 µg/mL, respectively. The orange calli (sample 41) had the highest echinacoside content among the controls. Wild-type stolon tissue (sample 6) contained only 702.5 µg/mL of echinacoside, highlighting the substantially higher accumulation levels achieved in the callus cultures under stress treatments. Cold stress (4 °C) produced only moderate echinacoside levels, with sample 50 reaching 18.8 µg/mL). Treatment with heavy metals such as CdCl_2_ and Cu(NO_3_)_2_ resulted in intermediate accumulation levels. Sample 77, for instance, yielded 1641.6 µg/mL (Table 2).

The highest concentration of acetylacteoside was observed in orange calli under standard (non-stress) conditions (sample 41: 57.8 µg/mL), followed by sample 38 (orange calli treated with 0.1% Na_2_CO_3_ for 50 days): 46.3 µg/mL. White calli exhibited moderate levels, notably sample 37 (NaCl, 50 days), with 27.1 µg/mL. In contrast, dark-pigmented callus tissues accumulated significantly lower amounts. For instance, sample 36 (dark, treated with NaCl, 50 days) contained only 5.1 µg/mL. The stolon sample (sample 6) exhibited a background concentration of 75.8 µg/mL, suggesting that callus lines cultivated under stress-free or mild-stress conditions could synthesize acetylacteoside at near-native levels. Overall, orange pigmentation and carbonate stress were positively associated with increased acetylacteoside biosynthesis.

In contrast to acetylacteoside, salidroside was most responsive to cold and heavy metal stress. The highest concentrations were observed in white callus tissues subjected to these abiotic stressors. Sample 72 (0.25 mM Cu(NO_3_)_2_, seven days) accumulated 27.0 µg/mL, while samples 65 and 50 (Cu(NO_3_)_2_, five days; cold stress, five days) contained 23.7 µg/mL and 19.0 µg/mL, respectively. Samples cultivated under standard conditions or NaCl stress accumulated much lower levels of salidroside, generally below 6.5 µg/mL. However, dark calli, such as sample 40, exhibited a slightly higher salidroside content (6.2 µg/mL) than orange-pigmented lines. These results suggest that low temperatures and moderate concentrations of heavy metals specifically promote salidroside accumulation, particularly in white callus tissues.

Although detected in fewer samples, tubuloside displayed a consistent pattern of accumulation under carbonate and salinity stress in orange and white callus types. The highest concentration was found in sample 41 (orange, no stress) at 47.9 µg/mL, followed by sample 38 (orange, 0.1% Na_2_CO_3_) at 42.6 µg/mL. Sample 37 (white, 0.1% NaCl) demonstrated notable accumulation as well (8.3 µg/mL), whereas the stolon sample contained only 3.0 µg/mL. Interestingly, no detectable tubuloside was found in cold- or copper-stressed samples. This finding further supports the hypothesis that salt-induced stress (NaCl or Na_2_CO_3_) stimulates tubuloside biosynthesis preferentially in callus lines exhibiting orange or white pigmentation.

Verbascoside exhibited the most consistent pattern among the four phenylethanoid glycosides (PhGs). The highest concentration was recorded in sample 41 (orange calli, no stress) at 84.7 µg/mL. This was followed by sample 38 (orange, Na_2_CO_3_) at 61.6 µg/mL and sample 37 (white, NaCl) at 51.8 µg/mL. These values considerably exceeded the stolon baseline of 28.1 µg/mL (sample 6). Dark calli samples, such as sample 36, demonstrated moderate accumulation (13.8 µg/mL), while values in heavy metal- and cold-stressed samples were generally below 1.0 µg/mL. These results suggest that verbascoside biosynthesis is significantly enhanced under saline or carbonate stress, particularly in orange-pigmented callus lines, while cold and heavy metal stress suppress its accumulation.

### 2.3. PhG Accumulation in Relation to Callus Color

Analyzing echinacoside concentration in differently colored callus lines revealed a clear association between color and metabolic activity (Table 2). This suggests that the callus color may serve as a phenotypic marker for secondary metabolite accumulation.

Among the evaluated samples, the orange callus (sample 38), cultivated on B5 medium supplemented with 0.1% Na_2_CO_3_ for 50 days, showed the highest concentration of echinacoside, reaching 13,378.9 µg/mL. Similarly, sample 41, an orange callus grown under standard (stress-free) conditions, accumulated 8102.0 µg/mL. This finding further supports the observation that orange pigmentation corresponds to an increased metabolic output in terms of PhG biosynthesis.

White callus tissues also exhibited substantial echinacoside accumulation under various stress conditions. For instance, sample 17, which was treated with 0.1% Na_2_CO_3_ for 20 days, accumulated 10,937.6 µg/mL, while sample 15, which was treated with 0.1% NaCl for 20 days, reached 10,175.1 µg/mL. Under standard conditions, sample 42 accumulated 5499.0 µg/mL, which is significantly higher than the content in dark-pigmented tissues. White calli also responded to cold and heavy metal stress. Sample 50 (cold stress, five days) and sample 77 (0.15 mM CdCl_2_, seven days) accumulated 18.8 µg/mL and 1641.6 µg/mL, respectively.

In contrast, dark callus tissues consistently demonstrated the lowest levels of echinacoside. Sample 36, which was treated with 0.1% NaCl for 50 days, accumulated 3851.3 µg/mL. Meanwhile, under standard conditions, sample 40 yielded only 408.7 µg/mL, which is nearly 17 times lower than that of orange calli (sample 41) under identical cultivation parameters. These results suggest that dark pigmentation may be associated with metabolic downregulation or alternative pathways not linked to PhG biosynthesis.

The stolon (sample 6), representing the wild-type vegetative organ, contained 702.5 µg/mL of echinacoside. This amount was exceeded by multiple callus lines, particularly those exhibiting white or orange pigmentation. For example, sample 38 (orange callus) accumulated nearly 19-fold more echinacoside than the stolon.

These results suggest that stress from Na_2_CO_3_ and NaCl, especially when applied over an extended period, can substantially increase the accumulation of echinacoside. Variations in callus pigmentation may reflect underlying differences in metabolic activity. Overall, these findings highlight the combined impact of stress type, exposure duration, and tissue pigmentation on PhG biosynthesis in *C. deserticola*. Callus pigmentation may serve as a simple, effective marker to identify high-yielding cell lines suitable for efficient PhG production.

## 3. Discussion

### 3.1. Compounds of Pharmaceutical Interest

The GC-MS analysis of *C. deserticola* callus and stolon samples revealed a variety of secondary metabolites with recognized pharmaceutical relevance. These metabolites play an essential role in plant physiology by contributing to signal transduction, stress adaptation, and defense responses. Many of the detected compounds possess bioactive properties, making them valuable for pharmaceutical, cosmetic, and nutraceutical applications (see Supplementary Materials S9). One prominent constituent is hexadecanoic acid, methyl ester (methyl palmitate), which was abundant in both stolon (11.8%) and callus (4.26% to 4.86%) tissues. Methyl palmitate is a well-known antibacterial agent with documented efficacy against multidrug-resistant bacterial strains [68]. It is also commonly used as an emollient in cosmetic products due to its skin-conditioning properties [69]. Interestingly, diethylene glycol monododecyl ether and tetraethylene glycol, which are typically classified as synthetic or industrial surfactants, were also detected in significant quantities. Diethylene glycol monododecyl ether was present at levels of up to 13.2% in stolons and 6.73% in callus tissue (sample 36), while tetraethylene glycol was present at levels of 11.5% in stolons (sample 6) and ranged from 1.69% to 3.79% across callus samples. The presence of these compounds may indicate environmental contamination or degradation products of naturally occurring polyethyleneglycol derivatives. Further investigation is required to clarify their origin and biological relevance in plant tissues. Another notable metabolite, γ-sitosterol, was detected at 19.01% in dark callus tissue (sample 40). This phytosterol contributes to membrane stability and lipid metabolism in plants. It is also known for its hypolipidemic, anti-inflammatory, and moisturizing properties in humans [70,71].

The most pharmacologically relevant group of compounds in *C. deserticola* is PhGs, including salidroside, echinacoside, and tubuloside [23,32,42,43,44,45]. These naturally occurring glycosides are characterized structurally by hydroxy- and methoxy-substituted phenylethyl and cinnamoyl moieties that are glycosidically and ester-linked to glucose units [21,32]. PhGs possess a wide range of bioactivities, including antibacterial, antioxidant, anti-inflammatory, antiviral, anticancer, antidiabetic, and neuroprotective effects [54]. In plants, PhGs are involved in stress response mechanisms and developmental processes, which underscores their physiological importance [22,31]. Some compounds identified by GC-MS may be biosynthetic precursors or byproducts related to PhG biosynthesis. For instance, phenolic derivatives, such as 2,4-bis(1,1-dimethylethyl) phenol, which is abundant in stolons (8.3%) and present at 1.44% to 2.27% in callus tissues, may participate in phenylpropanoid metabolism, which underlies PhG biosynthesis. In addition to their potential role as metabolic intermediates, these phenols are bioactive themselves and widely used as antioxidants in pharmaceutical and cosmetic formulations [56]. The presence of phenylpropanoids and benzoate derivatives in *C. deserticola* callus culture lines indicates active metabolic flux through the phenylalanine and tyrosine pathways. These pathways are known to be precursors for PhG biosynthesis (see Supplementary Materials S9). These findings support the presence of functional secondary metabolism in callus tissues, particularly in the phenylpropanoid pathway, which is a central route in the biosynthesis of a wide array of bioactive aromatic compounds, including PhGs. Additionally, the presence of fatty acids and aromatic alcohols, including homovanillyl alcohol (3.9% in stolons, 3.91% in yellow callus, and 2.80% in white callus), suggests their potential role in ester and glycoside biosynthesis. Homovanillyl alcohol, a metabolite derived from catecholamine degradation, exhibits antioxidant activity and has applications in cosmetics and pharmaceuticals [72,73].

The presence of carbohydrates and glycosides, such as ethyl α-D-glucopyranoside (23.71% to 44.30%) and sucrose (1.82% in sample 41), highlights their potential role as structural or regulatory intermediates in PhG biosynthesis. Ethyl α-D-glucopyranoside is commonly used industrially as a sweetening and moisturizing agent in industrial applications. Sucrose, on the other hand, is a key molecule in plant carbon metabolism that contributes to energy transport, signaling, and metabolic regulation [74]. Together, these results emphasize the intricate relationship between primary and secondary metabolisms in *C. deserticola* callus cultures and highlight the potential of tissue culture systems for studying and improving the production of pharmacologically relevant compounds.

### 3.2. PhG Accumulation in Callus Tissues Depends on Cultivation Conditions

The results reveal significant differences in the composition of secondary metabolites between the stolons (modified wild-type shoots) and callus tissues of *C. deserticola*. Callus samples exhibited notably higher concentrations of specific compounds associated with a response to stress factors that play a pivotal role in PhG biosynthesis (Supplementary Materials S9). *C. deserticola* is well known for its pharmacological potential. However, research on its in vitro cell and tissue culture systems is limited and underrepresented in the scientific literature [58,59].

Salt stress, particularly treatment with Na_2_CO_3_, significantly enhanced the accumulation of echinacoside, with the highest levels detected after 50 days of exposure to 0.1% Na_2_CO_3_. These results suggest that the osmotic imbalance and ion toxicity induced by carbonate ions act as potent elicitors that potentially upregulate genes involved in the PhG biosynthetic pathway [73]. In contrast, NaCl treatments resulted in comparatively lower PhG accumulation, indicating that carbonate-specific signaling plays a more decisive role in modulating secondary metabolism than sodium chloride alone.

Cold stress (4 °C) moderately increased salidroside concentrations, with maximal accumulation occurring after five days of treatment. This response is consistent with previous findings that low-temperature exposure activates stress-responsive metabolic pathways, particularly those associated with the biosynthesis of protective secondary metabolites [60,73]. Interestingly, the minimal impact on echinacoside under the same conditions suggests that cold stress preferentially enhances salidroside biosynthesis, possibly through the selective activation of different enzymatic pathways or transcriptional regulators. Exposure to heavy metal salts, such as Cu(NO_3_)_2_ and CdCl_2_, also resulted in increased PhG accumulation. Cadmium had a more pronounced effect on echinacoside production. This aligns with previous reports that heavy metal-induced oxidative stress can trigger defense-related metabolic networks in plant tissues [74]. Furthermore, the dose-dependent increase in PhGs under Cu(NO_3_)_2_ treatment indicates a correlation between the intensity of oxidative stress and the extent of biosynthetic activation.

Among all stress factors evaluated, the most substantial accumulation of echinacoside (13,378.9 µg/mL, HPLC-MS) occurred after 50 days of exposure (to 0.1% Na_2_CO_3_), highlighting the effectiveness of carbonate-induced stress in stimulating the biosynthesis of secondary metabolites. Salidroside concentrations consistently remained lower than those of echinacoside across treatments; however, notable increases were recorded in response to cold and heavy metal stress. These differential patterns of metabolite accumulation reflect the selective activation of biosynthetic pathways by specific stress factors. They also demonstrate the potential to enhance the in vitro production of pharmacologically important compounds by strategically manipulating the culture conditions.

### 3.3. Callus Color Correlates with PhG Accumulation

The variation observed in PhG content among the callus types of different colors suggests a strong metabolic association between tissue coloration and secondary metabolite biosynthesis. White- and orange-colored calli consistently demonstrated elevated levels of echinacoside, likely reflecting an enhanced activation of stress-responsive biosynthetic pathways. In contrast, dark calli exhibited markedly lower concentrations of PhGs, indicating the suppression of complex secondary metabolism or a metabolic shift toward producing simpler protective compounds, such as phenols and antioxidants. These substances may contribute to the dark color, as reported in previous studies [4,66].

Phenolic compounds, including chlorinated and methoxylated derivatives such as 2,4-bis(1,1-dimethylethyl)-phenol, 2,6-dimethoxy-; 2-methoxy-4-vinylphenol, are known to undergo oxidative polymerization, forming color structures such as flavonoids and melanin. These pathways are integral components of plants defense machinery under various stress conditions [75,76,77].

Salt stress induced by Na_2_CO_3_ had the most pronounced impact on echinacoside accumulation in orange calli among stress treatments. Conversely, cold stress at 4 °C was particularly effective in enhancing salidroside synthesis in predominantly white tissues. Heavy metal stress (Cu(NO_3_)_2_, CdCl_2_) also promoted PhG accumulation, particularly in orange calli, possibly through the activation of the signaling pathways related to oxidative stress.

## 4. Materials and Methods

### 4.1. Research Objects and Plant Collection Sites

**Figure 2:**
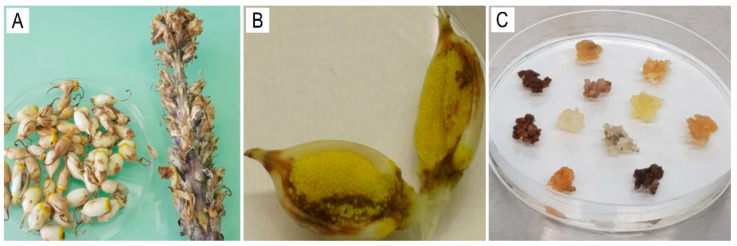
*C. deserticola* (**A**) seed capsule; (**B**) longitudinal section of a capsule; (**C**) calli induced from seeds, varying in color.

Shoots (stolons, Figure 2A) and seed capsules (Figure 2B) of *Cistanche deserticola* were collected in the Betpak-Dala desert (South Kazakhstan region; coordinates 46°02′00″ N, 70°12′00″ E). Figure 2C illustrates the colors of the induced callus clusters from the seeds after six months of cultivation.

### 4.2. In Vitro Cell and Tissue Culture

In our study, we used the established callus lines of *Cistanche deserticola* that were derived from seeds. The callus was cultured in Petri dishes containing Gamborg B5 medium [78], supplemented with 1.0 mg/L of 2,4-D, 0.5 mg/L of BAP, 10.0 mg/L of GA_3_, 1.0 mg/L of ascorbic acid, and 800 mg/L of casein hydrolysate. Phytogel was used as a gelling agent at a concentration of 2.0 g/L. Prior to sterilization, the pH of the medium was adjusted to 5.7–5.8. Subcultures were performed every four weeks. The Petri dishes were maintained in a growth chamber in the dark at 25 ± 2 °C with 70% relative humidity [79].

**Figure 3:**
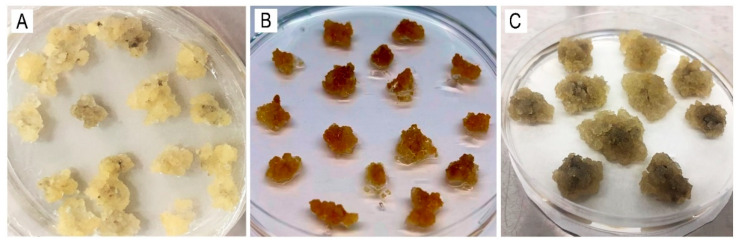
The *C. deserticola* callus lines: (**A**)—white; (**B**)—orange; (**C**)—dark.

The established *C. deserticola* callus lines exhibited distinct pigmentation phenotypes: A—white (Figure 3А), B—orange (Figure 3B), and C—dark calli (Figure 3C). To induce osmotic stress, sodium chloride (NaCl) and sodium carbonate (Na_2_CO_3_) were added to the nutrient medium at concentrations of 0.025%, 0.05%, and 0.1%. The calli were cultured for 20 and 50 days [62]. Cold stress was induced by transferring the callus cultures to a low temperature (+4 °C) for three, five, or seven days [80]. To induce oxidative stress, copper nitrate (Cu(NO_3_)_2_) was added to the nutrient medium at concentrations of 0.25, 0.5, and 1.0 millimolar (mM), and cadmium chloride (CdCl_2_) was added at concentrations of 0.02, 0.05, 0.15, and 0.2 mM. The calli were then incubated under standard temperature conditions for three, five, and seven days as a control [81,82,83]. Following the treatments, calli were harvested for metabolite analysis in accordance with established protocols and prior studies.

### 4.3. Lyophilization

The callus tissues were freeze-dried according to the protocol described by Elnaker et al. [84]. The samples were initially frozen at −20 °C and then transferred to −80 °C for 48 h. Finally, the samples were lyophilized using a LyoQuest freeze dryer (Telstar, Terrassa, Spain) for an additional 48 h. The resulting dried material was homogenized into a fine powder for further analysis.

### 4.4. Extraction of Secondary Metabolites

Secondary metabolites were extracted from 25 mg of lyophilized callus tissue by homogenizing it in 1000 μL of 80% methanol containing stainless steel beads in a TissueLyzer system (QIAGEN, Hilden, Germany) for 15 min. The homogenates were then centrifuged at 6000 rpm for five minutes, and the supernatant was collected. The remaining pellet was re-extracted with 500 μL of 80% methanol under the same conditions. The resulting supernatants from both extraction steps were pooled and filtered through 0.45 μm membrane filters (Millipore, Darmstadt, Germany) prior to analysis.

### 4.5. GC-MS Analysis

Volatile and semi-volatile metabolites were analyzed using gas chromatography–mass spectrometry (GC-MS) on an Agilent 7890A/5975C system (Agilent Technologies, Santa Clara, CA, USA). One microliter of the sample was injected in splitless mode at an inlet temperature of 250 °C. Separation was performed using a DB-WaxExt capillary column (30 m × 0.25 mm i.d., 0.25 μm film thickness; Agilent Technologies, Santa Clara, CA, USA) with helium as the carrier gas at a constant flow rate of 1 mL/min. The oven temperature was programmed as follows: an initial temperature of 40 °C increased at a rate of 10 °C/min to 260 °C and was held for 10 min. The total run time was 32 min.

Mass spectrometric detection was performed in SCAN mode over a mass-to-charge ratio (*m*/*z*) of 34–750. The GC-MS system control was conducted using Agilent MSD ChemStation software (v1701EA). Chromatographic peaks were analyzed based on retention time and peak area. Compounds were identified by comparing the obtained mass spectra with those in the Wiley 7th edition and NIST’11 libraries, which contain over 550,000 spectra.

Representative GC-MS chromatograms are provided in the Supplementary Materials (S8), with the retention time (min) plotted on the x-axis and relative abundance plotted on the y-axis.

### 4.6. UHPLC-MS Analysis of PhGs

The quantitative profiling of PhGs was carried out using a high-resolution quadrupole time-of-flight ultra-high-performance liquid chromatography–mass spectrometry (UHPLC-MS-qTOF) system (Impact II VIP, Bruker Daltonics, Bremen, Germany). Sixteen callus samples were tested against the high-purity commercial standards of PhGs, including salidroside, echinacoside, tubuloside, verbascoside, and acetylacteoside (all from Sigma-Aldrich, St. Louis, MO, USA), at a concentration of 0.001 mg/mL each. Representative UHPLC-MS chromatograms and calibration curves are provided in the Supplementary Materials (S9). The list of the reference standards of PhGs used for UHPLC-MS analysis is provided in the Supplementary Materials (Table S7).

The chromatographic conditions were as follows: separation was achieved using a C18 column, 100 × 2.1 mm, with a pore size of 100 Å (INT20051, Intensity Solo 2, Bruker Daltonics, Bremen, Germany), which was maintained at 30 °C. The injection volume was 2 μL, and the flow rate was set at 0.3 mL/min. The mobile phase consisted of A: ultrapure water containing 0.1% formic acid and B: MS-grade acetonitrile containing 0.1% formic acid. The elution gradient used for the separation is presented in Table 3.

The mass spectrometer operated in positive ion mode with SCAN acquisition over a mass-to-charge (*m*/*z*) range of 100–1000. Compound identification was based on accurate mass measurements, isotope pattern matching, and MS/MS fragmentation profiles.

Quantification was performed using calibration curves constructed for each PhG standard by serially diluting a 0.25 mg/mL stock solution across a defined concentration range, following the methodology of De Hoffmann and Stroobant [85]. The peak area (y) was plotted against the known concentration (x) of each standard, and a quadratic regression model was applied. This model enabled the accurate quantification of PhGs in the experimental samples. The regression models demonstrated high reliability, with all compounds showing coefficients of determination (R^2^) greater than 0.99, confirming strong linearity [86].

## 5. Conclusions

This study presents the first comprehensive, comparative analysis of the secondary metabolite profiles of *C. deserticola* calli that were established de novo under in vitro conditions and benchmarked against wild-type stolon samples. A diverse set of abiotic stress factors, including salt stress (NaCl and Na_2_CO_3_), cold exposure, and heavy metal salts (Cu(NO_3_)_2_, CdCl_2_), was used to evaluate their impact on the biosynthesis of pharmacologically important relevant compounds, particularly PhGs. The results clearly demonstrate the robust biosynthetic potential of *C. deserticola* calli, which, in many cases, surpassed the metabolite concentrations found in natural stolons.

The quantitative profiling of phenylethanoid glycosides (PhGs) in *Cistanche deserticola* callus cultures revealed that abiotic stress conditions and callus pigmentation significantly influence metabolite accumulation. Treatments involving salinity (NaCl) and carbonate (Na_2_CO_3_) markedly increased the production of echinacoside, acetylacteoside, tubuloside, and verbascoside, especially in orange and white callus lines. Several samples exceeded native stolon levels. Conversely, salidroside accumulation was primarily stimulated by cold and heavy metal stress, particularly in white calli. Orange pigmentation and carbonate stress were strongly associated with increased PhG biosynthesis. This highlights the importance of physiological and environmental factors in optimizing secondary metabolite production in vitro.

In conclusion, this study establishes an effective platform for the controlled production of bioactive PhGs in *C. deserticola* callus cultures. Strategically manipulating culture conditions and using visible pigmentation as a selection criterion allows for the optimization of secondary metabolite yields while conserving natural plant resources. These findings highlight the biotechnological potential of in vitro culture systems as a scalable and ethical alternative for producing medicinal compounds derived from plants, with promising applications in the pharmaceutical, nutraceutical, and functional cosmetic industries.

## Figures and Tables

**Table 1:** GC-MS-based identification and relative content (%) of biologically active compounds in callus and stolon samples of *C. deserticola* identified by GC-MS analysis.

Identified Compounds	Percentage Content, %				
	No. 6, Stolon, Control	No. 36, B5, 0.1% NaCl, 50 days (Dark)	No. 40, B5, 50 days (Dark)	No. 41, B5, 50 days (Orange)	No. 42, B5, 50 days (White) Control
Phenols					
Phenol	-	0.77	0.69	0.82	0.97
Phenol, 4-ethyl-2-methoxy-	-	-	-	0.36	0.59
p-Cresol	-	-	-	1.46	0.63
Benzene, 4-ethenyl-1,2-dimethoxy-	6.7	-	-	-	-
Phenol, 2,4-dichloro-	-	-	0.48	0.55	0.53
2-Methoxy-4-vinylphenol	-	-	-	4.55	2.14
Phenol, 2,6-dimethoxy-	-	0.99	0.59	2.7	1.08
Phenol, 2,4-bis(1,1-dimethylethyl)-	8.3	2.27	1.7	1.88	1.44
Phenol, 2,6-dimethoxy-4-(2-propenyl)-	-	1.51	1.02	7.33	1.43
Benzeneethanol, 4-hydroxy-	-	1.59	1.44	1.04	0.71
Hydroquinone	-	-	-	-	0.42
Ethers					
Dodecyl acrylate	-	0.6	0.66	0.42	0.38
Glutaric acid, butyl undecyl ester	2.9	-	-	-	-
Hexadecanoic acid, methyl ester	11.8	4.26	4.37	4.42	4.86
Hexadecanoic acid, ethyl ester	8.5	-	-	-	-
Heptadecanoic acid, 16-methyl-, methyl ester	10.5	2.34	1.86	1.41	1.15
13-Octadecenoic acid, methyl ester	-	2.11	1.79	1.52	-
11-Octadecenoic acid, methyl ester, (Z)-	-	-	-	-	1.45
12-Octadecenoic acid, methyl ester	-	-	-	0.55	-
9,12-Octadecadienoic acid (Z,Z)-, methyl ester	-	4.12	0.24	-	4.29
Methyl 9-cis,11-trans-octadecadienoate	-	-	-	3.6	-
9,12-Octadecadienoic acid (Z,Z)-, methyl ester	-	-	-	-	-
Propanoic acid, 3-mercapto-, dodecyl ester	-	0.58	0.59	-	-
9,12,15-Octadecatrienoic acid, methyl ester, (Z,Z,Z)-	-	0.86	0.59	0.83	0.83
Acetic acid n-octadecyl ester	-	0.64	-	-	-
Diethylene glycol monododecyl ether	13.2	0.88	1.92	0.36	-
Triethylene glycol monododecyl ether	-	2.77	-	-	-
Heptaethylene glycol monododecyl ether	-	-	2.42	1.95	-
Eicosanoic acid, methyl ester	-	-	0.62	-	-
Phthalic acid, butyl hept-4-yl ester	-	-	0.45	-	-
Dibutyl phthalate	-	0.53	-	-	-
Di-n-octyl phthalate	-	-	2.14	-	-
1,4-Benzenedicarboxylic acid, bis(2-ethylhexyl) ester	-	-	9.37	-	-
Glucosides					
1,4:3,6-Dianhydro-α-d-glucopyranose	-	-	-	0.58	0.59
β-D-Glucopyranose, 1,6-anhydro-	-	-	5.25	8.15	8.23
Ethyl α-d-glucopyranoside	-	44.3	24.94	23.71	41.78
Sucrose	-	-	-	1.82	-
Alcohols					
Ethanol, 2,2′-oxybis-	-	0.86	0.66	-	-
1-Undecanol	-	1.37	-	1.16	-
5-Thiazoleethanol, 4-methyl-	-	-	-	3.38	2.16
1-Hexadecanol	-	0.9	1.24	0.6	-
Homovanillyl alcohol	3.9	1.23	0.9	3.91	2.8
Glycols					
Triethylene glycol	3.3	-	1.81	-	-
Tetraethylene glycol	11.5	3.79	2.1	2.19	1.69
Tri(propylene glycol) propyl ether	-	0.62	-	-	-
Triethylene glycol monododecyl ether	-	6.73	3.88	1.72	-
Pentaethylene glycol	-	-	-	2.04	1.8
Hexaethylene glycol	-	3.46	-	-	-
Acids					
Hexadecanoic acid	-	0.44	3.48	4.55	3.34
9,12-Octadecadienoic acid (Z,Z)-	-	3.5	-	2.15	2.98
Niacinamide	-	-	-	1.47	1.05
Ketones					
Ethanone, 1-(2-hydroxy-5-methylphenyl)-	9.4	1.65	0.8	-	-
3′,5′-Dimethoxyacetophenone	-	-	-	1.88	0.71
Ethanone, 1-(3,4-dimethoxyphenyl)-	-	0.92	-	-	-
Furans					
Benzofuran, 2,3-dihydro-	5.4	0.82	0.5	1.2	0.74
Phytosterols					
γ-Sitosterol	-	-	19.01	-	-
Pyrroles					
3-Methyl-4-phenyl-1H-pyrrole	-	0.57	-	-	0.59
1H-Pyrrole, 2-phenyl-	-	-	-	1.15	0.97
Lactones					
Pantolactone	-	0.54	-	0.85	0.75
Others					
Octadecanal, 2-bromo-	4.8	-	-	-	-
2-(3,4-Dimethoxyphenyl)-6-methyl-3,4-chromanediol	-	-	0.63	-	-
3,7,11,14,18-Pentaoxa-2,19-disilaeicosane, 2,2,19,19-tetramethyl-	-	1.49	-	-	-
Succinimide	-	-	-	-	0.45
Pyrrolo [1,2-a]pyrazine-1,4-dione, hexahydro-3-(2-methylpropyl)-	-	-	1.88	1.17	1.61
2-Piperidinone, 1-(3,4,5,6-tetrahydro-2-pyridinyl)-	-	-	-	-	4.58
Desaspidinol	-	-	-	0.54	0.27

**Table 2:** Concentration of PhGs in qTOF HPLC-MS fractions of the studied *C. deserticola* callus samples under different cultivation conditions.

No.	Sample No.	Growing Conditions					PhG Concentration (µg/mL)				
		Stress	Culture Medium	Conditions	Tissue Color	Days of Cultivation	Acetylacteoside	Echinacoside	Salidroside	Tubuloside	Verbascoside
1.	6	Control	Stolon	-	-	-	75.8	702.5	0.6	3.0	28.1
2.	15	NaCl	В5	0.1%	White	20	20.3	10,175.1	4.1	0	0
3.	17	Na_2_CO_3_	В5	0.1%	White	20	10.5	10,937.6	5.4	0	0
4.	36	NaCl	В5	0.1%	Dark	50	5.1	3851.3	2.7	0.9	13.8
5.	37	NaCl	В5	0.1%	White	50	27.1	10,615.4	1.8	8.3	51.8
6.	38	Na_2_CO_3_	В5	0.1%	Orange	50	46.3	13,378.9	2.5	42.6	61.6
7.	40	No stress	В5	Standard	Dark	50	2.0	408.7	6.2	0	0
8.	41	No stress	В5	Standard	Orange	50	57.8	8102.0	1.6	47.9	84.7
9.	42	No stress	В5	Standard	White	50	7.9	5499.0	1.4	2.0	32.0
10.	45	Cold	В5	+4 °C	White	3	0	10.9	13.5	0	0.1
11.	50	Cold	В5	+4 °C	White	5	0	18.8	19.0	0	0.1
12.	55	Cold	В5	+4 °C	White	7	0	12.9	13.3	0	0.1
13.	65	Cu(NO_3_)_2_	В5	0.25 mM	White	5	0	17.8	23.7	0	0.1
14.	70	CdCl_2_	В5	0.15 mM	White	5	0.3	144.6	18.5	0.3	0.8
15.	72	Cu(NO_3_)_2_	В5	0.25 mM	White	7	0	0.1	27.0	0	0.1
16.	77	CdCl_2_	В5	0.15 mM	White	7	2.3	1641.6	9.6	3.7	9.2

**Table 3:** UHPLC-MS gradient protocol.

Time (min)	A (%)	B (%)
0	95	5
2	95	5
4	85	15
6	80	20
10	65	35
18	65	35
18.1	95	5

## Data Availability

The raw data, including GC-MS and HPLC-MS chromatograms, are available in the electronic Supplementary Materials (S8 and S9).

## Supplementary Materials

The following supporting information can be downloaded at https://www.mdpi.com/article/10.3390/ijms26136091/s1.
