# Supplementary material for: Stress-Induced Secondary Metabolite Profiling in Cistanche deserticola Callus Cultures: Insights from GC-MS and HPLC-MS Analysis
Source: Int J Mol Sci. 2025 Jun 25;26(13):6091. doi: 10.3390/ijms26136091 (PMC12250269; doi:10.3390/ijms26136091)

File :C:\msdchem\1\data\Mereke\2024\June\270624_liq\s_06.D
Operator : IMV-HP\admin
Acquired : 27 Jun 2024 20:18 using AcqMethod liq_2023.M
Instrument : 7890
Sample Name: s_06
Misc Info :
Vial Number: 2

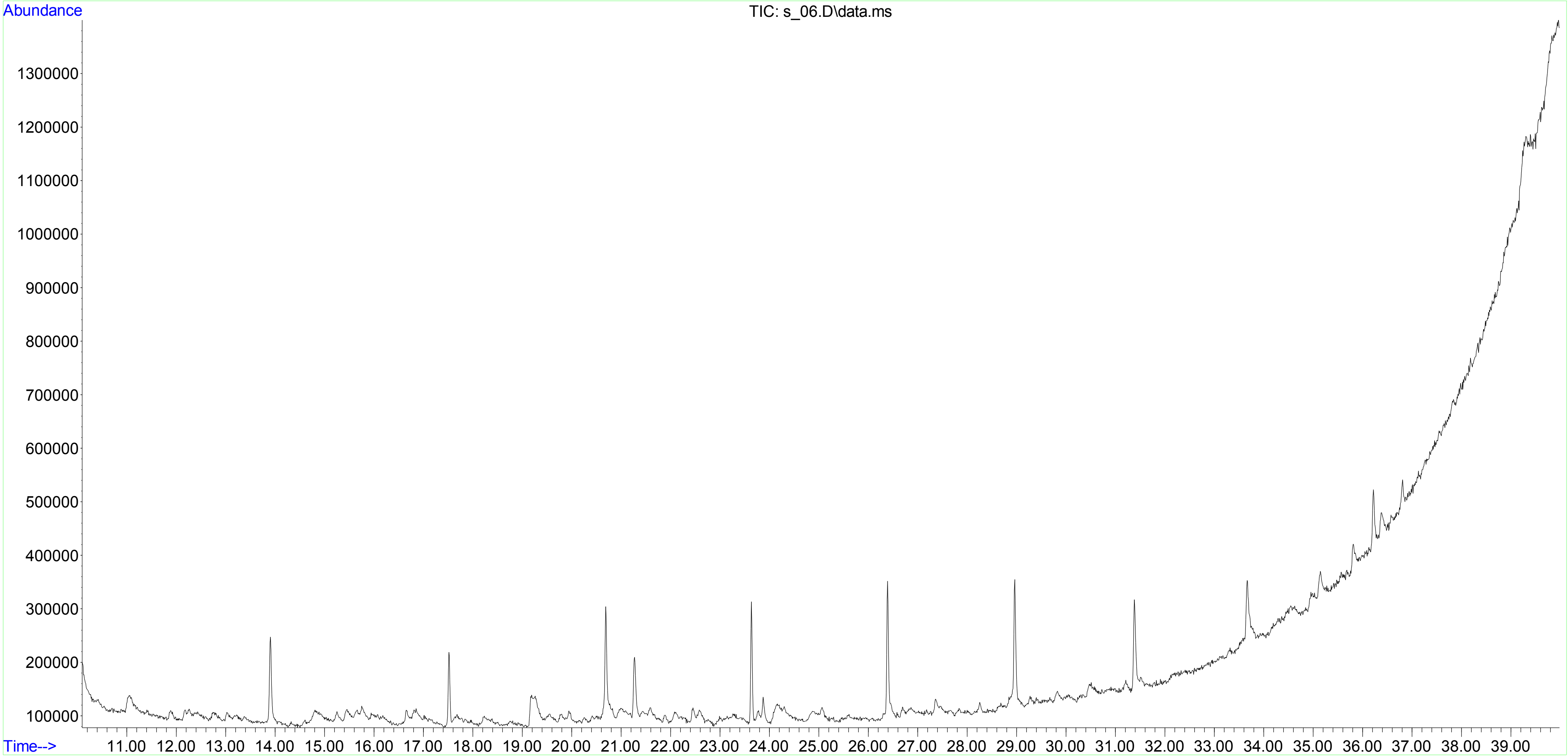

Supplement: Supplementary file 1 [file ijms-26-06091-s001.zip › Supplementary materials S8_GC-MS_Data/GC_6.pdf]
