# Supplementary material for: Stress-Induced Secondary Metabolite Profiling in Cistanche deserticola Callus Cultures: Insights from GC-MS and HPLC-MS Analysis
Source: Int J Mol Sci. 2025 Jun 25;26(13):6091. doi: 10.3390/ijms26136091 (PMC12250269; doi:10.3390/ijms26136091)

# 28.04.2025 – Calibration Functions

| | | | |
|---|---|---|---|
| Operator | Demo User | Station Name | impact_II-10436 |
| Instrument Name | impact II | Instrument SN | 1825265.10436 |
| TASQ Method | PhGs (28) | | |

## Calibration Functions

### Acetylacteoside

C31H38O16 M-nH *m/z* = 665.209 9.70 min

$y = -5240x^2 + 252060x - 5396$

| | |
|---|---|
| $R^2$ | 0.99976 |
| RSD RF | 6.541 |
| Creation Date | 2025-05-12 19:12:14 |
| Internal Standard | |
| Regression | quadratic |
| Weighting | 1/x |
| Origin | IGNOREZERO |
| Signal | AREA |
| Min Conc | 0.10000 ppm |
| Max Conc | 10.000 ppm |

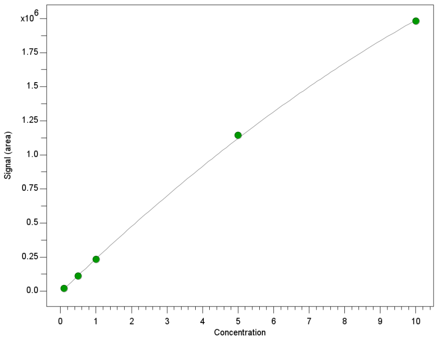

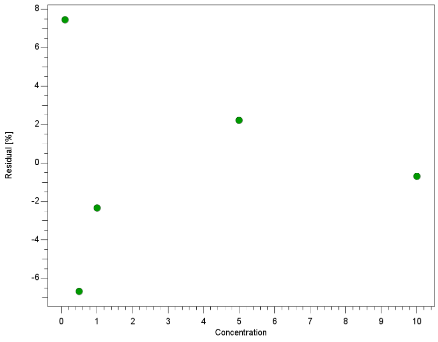


### Echinacoside

C35H46O20 M-nH *m/z* = 785.251 7.20 min

$y = -10.56x^2 + 7307x - 1420$

| | |
|---|---|
| $R^2$ | 1.00000 |
| RSD RF | 20.21 |
| Creation Date | 2025-05-12 19:12:23 |
| Internal Standard | |
| Regression | quadratic |
| Weighting | 1/x |
| Origin | IGNOREZERO |
| Signal | AREA |
| Min Conc | 0.5000 ppm |
| Max Conc | 10.000 ppm |

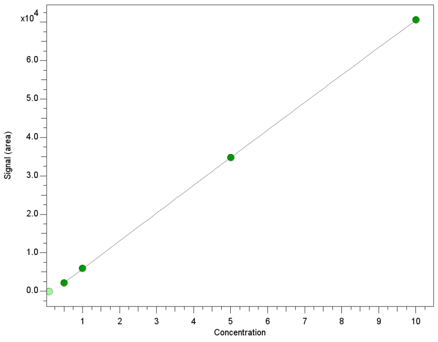

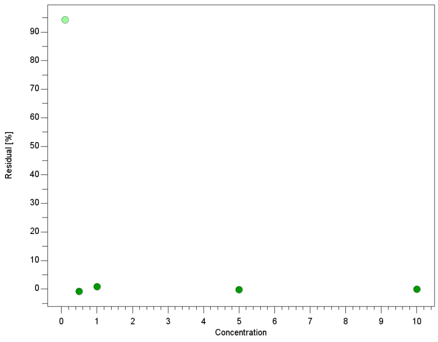


### Salidroside

C14H20O7 M-nH *m/z* = 299.114 5.50 min

$y = -2404x^2 + 115848x + 596.8$

| | |
|---|---|
| $R^2$ | 0.99997 |
| RSD RF | 10.61 |
| Creation Date | 2025-05-12 19:12:26 |
| Internal Standard | |
| Regression | quadratic |
| Weighting | 1/x |
| Origin | IGNOREZERO |
| Signal | AREA |
| Min Conc | 0.10000 ppm |
| Max Conc | 10.000 ppm |

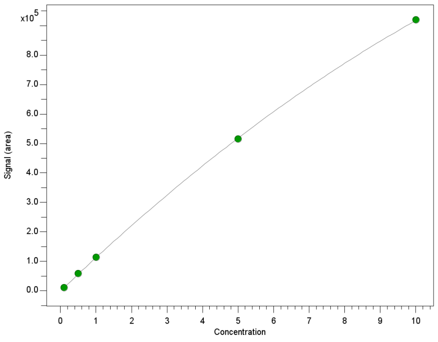

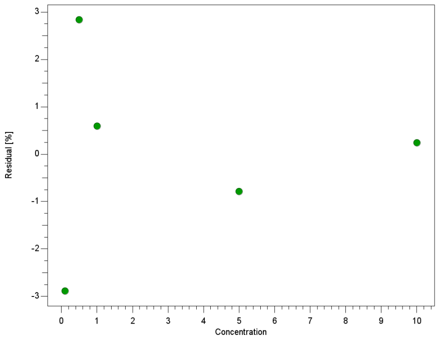

**Tubuloside** C37H48O21 M-nH *m/z* = 827.262 8.40 min

$y = -11124x^2 + 407731x - 3957$

| | |
|---|---|
| $R^2$ | 0.99991 |
| RSD RF | 10.68 |
| Creation Date | 2025-05-12 19:12:31 |
| Internal Standard | |
| Regression | quadratic |
| Weighting | 1/x |
| Origin | IGNOREZERO |
| Signal | AREA |
| Min Conc | 0.10000 ppm |
| Max Conc | 10.000 ppm |

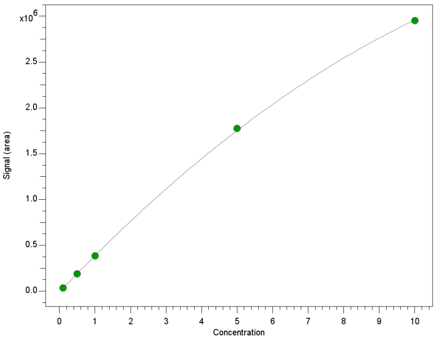


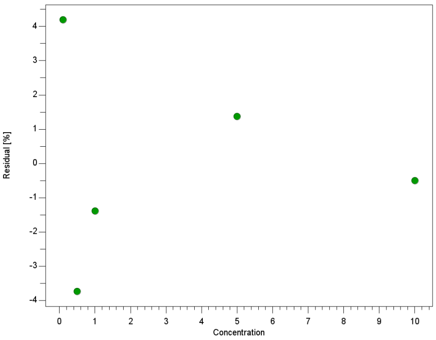


**Verbascoside** C29H36O15 M-nH *m/z* = 623.198 8.60 min

$y = -19680x^2 + 616938x + 2742$

| | |
|---|---|
| $R^2$ | 0.99984 |
| RSD RF | 16.07 |
| Creation Date | 2025-05-12 19:12:34 |
| Internal Standard | |
| Regression | quadratic |
| Weighting | 1/x |
| Origin | IGNOREZERO |
| Signal | AREA |
| Min Conc | 0.10000 ppm |
| Max Conc | 10.000 ppm |

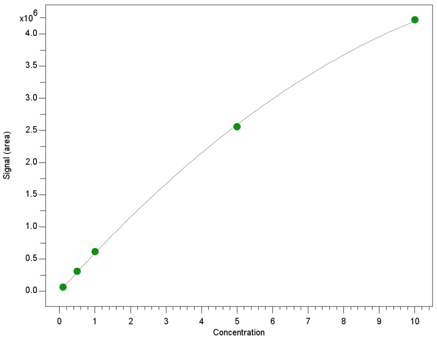


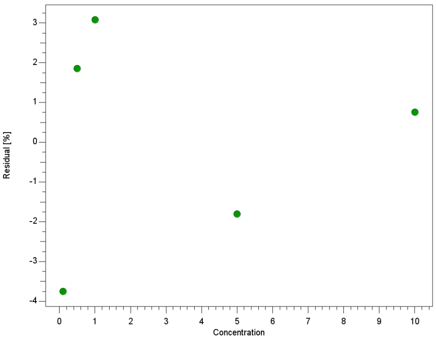

Supplement: Supplementary file 1 [file ijms-26-06091-s001.zip › Supplementary materials S9_qTOF_UHPLC-MS_Data/Application_1 – Calibration Functions.pdf]
