# Supplementary material for: Stress-Induced Secondary Metabolite Profiling in Cistanche deserticola Callus Cultures: Insights from GC-MS and HPLC-MS Analysis
Source: Int J Mol Sci. 2025 Jun 25;26(13):6091. doi: 10.3390/ijms26136091 (PMC12250269; doi:10.3390/ijms26136091)
Supplement: Supplementary file 1 [file ijms-26-06091-s001.zip › Supplementary materials S9_qTOF_UHPLC-MS_Data/Application_2 – Samples Screening and Quantitation.pdf]

Operator	Demo User	Station Name	impact_II-10436
Instrument Name	impact II	Instrument SN	1825265.10436
TASQ Method	PhGs (28)		

Analysis: blank_1_1_1197

Creation Date	2025-04-29 21:27	Sample Type	Blank
Method	PhGs (28)	Mass Calib. Date	
Station Name	impact_II-10436	Operator	Demo User
Instrument	impact II	Instrument SN	1825265.10436
Data Path	D:\Data\Zhanpeis_Issayev\PhGs\28.04.2025\blank_1_1_1197.d		

Screening Results

Analyte	Chromatogram

Analysis: Sample_41_3_1_1070

Creation Date	2025-03-15 22:14	Sample Type	Sample
Method	PhGs (28)	Mass Calib. Date	
Station Name	impact_II-10436	Operator	Demo User
Instrument	impact II	Instrument SN	1825265.10436
Data Path	D:\Data\Zhanpeis_Issayev\PhGs\Sample_41_3_1_1070.d		

Screening Results

Analyte	m/z exp.	Δ m/z [ppm]	Δ RT [min]	Mand Ions	Quantity	RT theo [min]	m/z Score	RT Score	Ions Score	R^2	Chromatogram
Acetylacteoside	665.2087	1.34	-0.03	2/2	57.8 ppm	9.70	●●●	●●●	●●●	0.9919	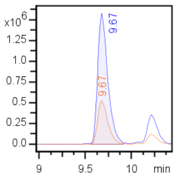
Echinacoside	785.2510	1.09	-0.03	2/2	8102.0 ppm	7.20	●●●	●●●	●●●	1.0000	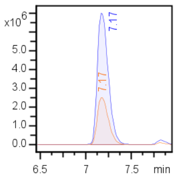
Salidroside	299.1136	1.04	0.02	2/2	1.6 ppm	5.50	●●●	●●●	●●●	0.9945	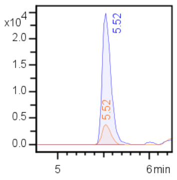
Tubuloside	827.2615	1.52	-0.03	2/2	47.9 ppm	8.40	●●●	●●●	●●●	0.9859	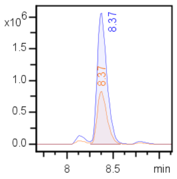
Verbascoside	623.1981	1.21	-0.04	2/2	84.7 ppm	8.60	●●●	●●●	●●●	0.9828	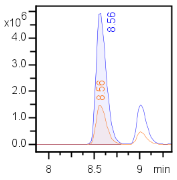

Analysis: Sample_6_4_1_1071

Creation Date	2025-03-17 16:00	Sample Type	Sample
Method	PhGs (28)	Mass Calib. Date	
Station Name	impact_II-10436	Operator	Demo User
Instrument	impact II	Instrument SN	1825265.10436
Data Path	D:\Data\Zhanpeis_Issayev\PhGs\Sample_6_4_1_1071.d		

Screening Results

Analyte	m/z exp.	Δ m/z [ppm]	Δ RT [min]	Mand Ions	Quantity	RT theo [min]	m/z Score	RT Score	Ions Score	R^2	Chromatogram
Acetylacteoside	665.2087	1.14	-0.03	2/2	75.8 ppm	9.70	●●●	●●●	●●●	0.9919	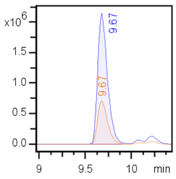
Echinacoside	785.2510	0.62	-0.05	2/2	702.5 ppm	7.20	●●●	●●●	●●●	1.0000	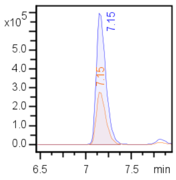
Salidroside	299.1136	0.88	0.04	2/2	0.6 ppm	5.50	●●●	●●●	●●●	0.9945	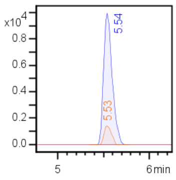
Tubuloside	827.2615	1.01	-0.03	2/2	3.0 ppm	8.40	●●●	●●●	●●●	0.9859	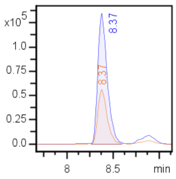
Verbascoside	623.1981	0.58	-0.04	2/2	28.1 ppm	8.60	●●●	●●●	●●●	0.9828	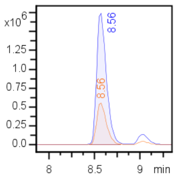

Analysis: Sample_42_8_1_1075

Creation Date	2025-03-17 17:55	Sample Type	Sample
Method	PhGs (28)	Mass Calib. Date	
Station Name	impact_II-10436	Operator	Demo User
Instrument	impact II	Instrument SN	1825265.10436
Data Path	D:\Data\Zhanpeis_Issayev\PhGs\Sample_42_8_1_1075.d		

Screening Results

Analyte	m/z exp.	Δ m/z [ppm]	Δ RT [min]	Mand Ions	Quantity	RT theo [min]	m/z Score	RT Score	Ions Score	R^2	Chromatogram
Acetylacteoside	665.2087	-2.75	0.03	2/2	7.9 ppm	9.70	●●●	●●●	●●●	0.9919	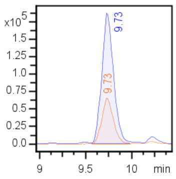
Echinacoside	785.2510	-2.51	-0.03	2/2	5499.0 ppm	7.20	●●●	●●●	●●●	1.0000	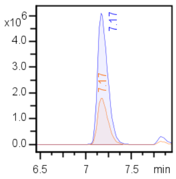
Salidroside	299.1136	-1.63	0.03	2/2	1.4 ppm	5.50	●●●	●●●	●●●	0.9945	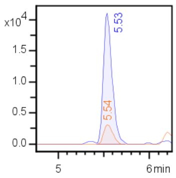
Tubuloside	827.2615	-2.30	-0.02	2/2	2.0 ppm	8.40	●●●	●●●	●●●	0.9859	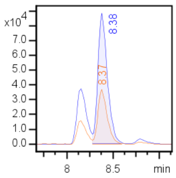
Verbascoside	623.1981	-2.78	-0.04	2/2	32.0 ppm	8.60	●●●	●●●	●●●	0.9828	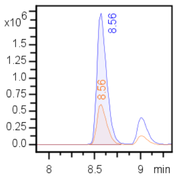

Analysis: Sample_36_9_1_1076

Creation Date	2025-03-17 18:23	Sample Type	Sample
Method	PhGs (28)	Mass Calib. Date	
Station Name	impact_II-10436	Operator	Demo User
Instrument	impact II	Instrument SN	1825265.10436
Data Path	D:\Data\Zhanpeis_Issayev\PhGs\Sample_36_9_1_1076.d		

Screening Results

Analyte	m/z exp.	Δ m/z [ppm]	Δ RT [min]	Mand Ions	Quantity	RT theo [min]	m/z Score	RT Score	Ions Score	R²	Chromatogram
Acetylacteoside	665.2087	-2.69	0.04	2/2	5.1 ppm	9.70	●●●	●●●	●●●	0.9919	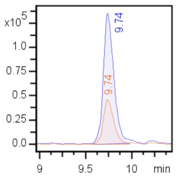
Echinacoside	785.2510	-2.51	-0.05	2/2	3851.3 ppm	7.20	●●●	●●●	●●●	1.0000	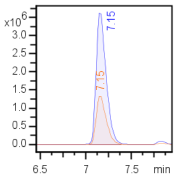
Salidroside	299.1136	-1.26	0.02	2/2	2.7 ppm	5.50	●●●	●●●	●●●	0.9945	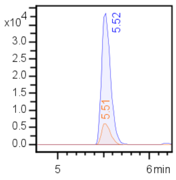
Tubuloside	827.2615	-2.40	-0.03	2/2	0.9 ppm	8.40	●●●	●●●	●●●	0.9859	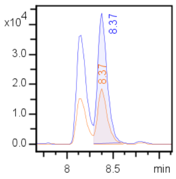
Verbascoside	623.1981	-2.66	-0.04	2/2	13.8 ppm	8.60	●●●	●●●	●●●	0.9828	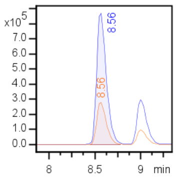

Analysis: Sample_37_10_1_1077

Creation Date	2025-03-17 18:52	Sample Type	Sample
Method	PhGs (28)	Mass Calib. Date	
Station Name	impact_II-10436	Operator	Demo User
Instrument	impact II	Instrument SN	1825265.10436
Data Path	D:\Data\Zhanpeis_Issayev\PhGs\Sample_37_10_1_1077.d		

Screening Results

Analyte	m/z exp.	Δ m/z [ppm]	Δ RT [min]	Mand Ions	Quantity	RT theo [min]	m/z Score	RT Score	Ions Score	R²	Chromatogram
Acetylacteoside	665.2087	-3.44	0.02	2/2	27.1 ppm	9.70	●●●	●●●	●●●	0.9919	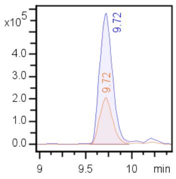
Echinacoside	785.2510	-2.55	-0.06	2/2	10615.4 ppm	7.20	●●●	●●●	●●●	1.0000	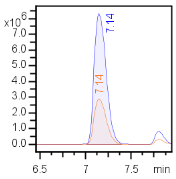
Salidroside	299.1136	-1.37	0.03	2/2	1.8 ppm	5.50	●●●	●●●	●●●	0.9945	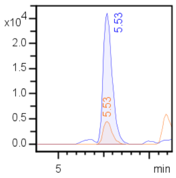
Tubuloside	827.2615	-3.14	-0.05	2/2	8.3 ppm	8.40	●●●	●●●	●●●	0.9859	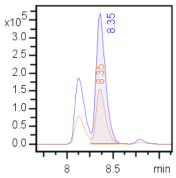
Verbascoside	623.1981	-3.27	-0.05	2/2	51.8 ppm	8.60	●●●	●●●	●●●	0.9828	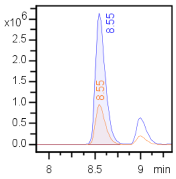

Analysis: Sample_38_11_1_1078

Creation Date	2025-03-17 19:21	Sample Type	Sample
Method	PhGs (28)	Mass Calib. Date	
Station Name	impact_II-10436	Operator	Demo User
Instrument	impact II	Instrument SN	1825265.10436
Data Path	D:\Data\Zhanpeis_Issayev\PhGs\Sample_38_11_1_1078.d		

Screening Results

Analyte	m/z exp.	Δ m/z [ppm]	Δ RT [min]	Mand Ions	Quantity	RT theo [min]	m/z Score	RT Score	Ions Score	R²	Chromatogram
Acetylacteoside	665.2087	-2.65	-0.02	2/2	46.3 ppm	9.70	●●●	●●●	●●●	0.9919	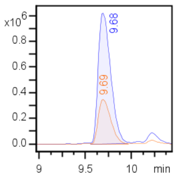
Echinacoside	785.2510	-2.67	-0.03	2/2	13378.9 ppm	7.20	●●●	●●●	●●●	1.0000	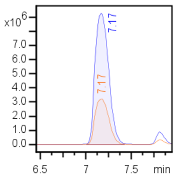
Salidroside	299.1136	-2.81	0.02	2/2	2.5 ppm	5.50	●●●	●●●	●●●	0.9945	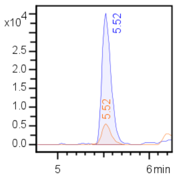
Tubuloside	827.2615	-2.74	-0.03	2/2	42.6 ppm	8.40	●●●	●●●	●●●	0.9859	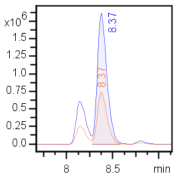
Verbascoside	623.1981	-2.84	-0.04	2/2	61.6 ppm	8.60	●●●	●●●	●●●	0.9828	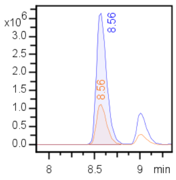

Analysis: Sample_65_12_1_1079

Creation Date	2025-03-17 19:54	Sample Type	Sample
Method	PhGs (28)	Mass Calib. Date	
Station Name	impact_II-10436	Operator	Demo User
Instrument	impact II	Instrument SN	1825265.10436
Data Path	D:\Data\Zhanpeis_Issayev\PhGs\Sample_65_12_1_1079.d		

Screening Results

Analyte						Chromatogram
	m/z exp.	Δ m/z [ppm]	Δ RT [min]	Mand Ions	Quantity	
Echinacoside	785.2510	-0.79	-0.09	2/2	17.8 ppm	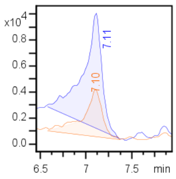
	RT theo [min]	m/z Score	RT Score	Ions Score	R^2	
	7.20	●●●	●●●	●●●	1.0000	
	m/z exp.	Δ m/z [ppm]	Δ RT [min]	Mand Ions	Quantity	
Salidroside	299.1136	1.25	0.01	2/2	23.7 ppm	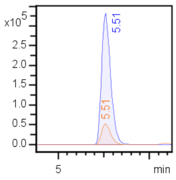
	RT theo [min]	m/z Score	RT Score	Ions Score	R^2	
	5.50	●●●	●●●	●●●	0.9945	
	m/z exp.	Δ m/z [ppm]	Δ RT [min]	Mand Ions	Quantity	
Verbascoside	623.1981	0.21	-0.07	2/2	0.1 ppm	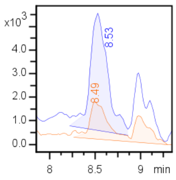
	RT theo [min]	m/z Score	RT Score	Ions Score	R^2	
	8.60	●●●	●●●	●●●	0.9828	

Analysis: Sample_72_13_1_1080

Creation Date	2025-03-17 20:23	Sample Type	Sample
Method	PhGs (28)	Mass Calib. Date	
Station Name	impact_II-10436	Operator	Demo User
Instrument	impact II	Instrument SN	1825265.10436
Data Path	D:\Data\Zhanpeis_Issayev\PhGs\Sample_72_13_1_1080.d		

Screening Results

Analyte						Chromatogram
	m/z exp.	Δ m/z [ppm]	Δ RT [min]	Mand Ions	Quantity	
Salidroside	299.1136	-2.08	0.01	2/2	27.0 ppm	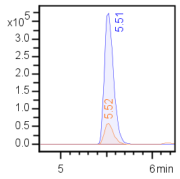
	RT theo [min]	m/z Score	RT Score	Ions Score	R^2	
	5.50	●●●	●●●	●●●	0.9945	
	m/z exp.	Δ m/z [ppm]	Δ RT [min]	Mand Ions	Quantity	
Verbascoside	623.1981	-2.30	-0.04	2/2	0.1 ppm	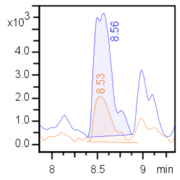
	RT theo [min]	m/z Score	RT Score	Ions Score	R^2	
	8.60	●●●	●●●	●●●	0.9828	

Analysis: Sample_45_14_1_1082

Creation Date	2025-03-17 21:21	Sample Type	Sample
Method	PhGs (28)	Mass Calib. Date	
Station Name	impact_II-10436	Operator	Demo User
Instrument	impact II	Instrument SN	1825265.10436
Data Path	D:\Data\Zhanpeis_Issayev\PhGs\Sample_45_14_1_1082.d		

Screening Results

Analyte	m/z exp.	Δ m/z [ppm]	Δ RT [min]	Mand Ions	Quantity	RT theo [min]	m/z Score	RT Score	Ions Score	R^2	Chromatogram
Echinacoside ✍	785.2510	3.76	-0.03	2/2	10.9 ppm	7.20	●●●	●●●	●●●	1.0000	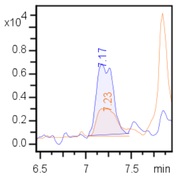
Salidroside	299.1136	-1.30	0.01	2/2	13.5 ppm	5.50	●●●	●●●	●●●	0.9945	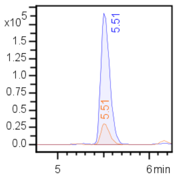
Verbascoside ✍	623.1981	0.46	0.00	2/2	0.0 ppm	8.60	●●●	●●●	●●●	0.9828	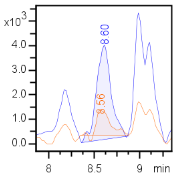

Analysis: Sample_50_15_1_1083

Creation Date	2025-03-17 21:49	Sample Type	Sample
Method	PhGs (28)	Mass Calib. Date	
Station Name	impact_II-10436	Operator	Demo User
Instrument	impact II	Instrument SN	1825265.10436
Data Path	D:\Data\Zhanpeis_Issayev\PhGs\Sample_50_15_1_1083.d		

Screening Results

Analyte	m/z exp. / RT theo [min]	Δ m/z [ppm] / m/z Score	Δ RT [min] / RT Score	Mand Ions / Ions Score	Quantity / R^2	Chromatogram
Echinacoside	785.2510	-2.73	-0.05	2/2	18.8 ppm	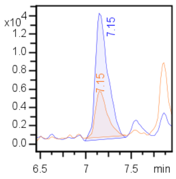
	7.20	●●●	●●●	●●●	1.0000	
Salidroside	299.1136	-1.31	0.01	2/2	19.0 ppm	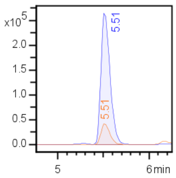
	5.50	●●●	●●●	●●●	0.9945	
Verbascoside	623.1981	0.84	-0.03	2/2	0.1 ppm	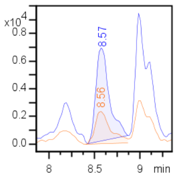
	8.60	●●●	●●●	●●●	0.9828	

Analysis: Sample_55_16_1_1085

Creation Date	2025-03-17 22:47	Sample Type	Sample
Method	PhGs (28)	Mass Calib. Date	
Station Name	impact_II-10436	Operator	Demo User
Instrument	impact II	Instrument SN	1825265.10436
Data Path	D:\Data\Zhanpeis_Issayev\PhGs\Sample_55_16_1_1085.d		

Screening Results

Analyte	m/z exp.	Δ m/z [ppm]	Δ RT [min]	Mand Ions	Quantity	RT theo [min]	m/z Score	RT Score	Ions Score	R^2	Chromatogram
Echinacoside	785.2510	-1.23	-0.01	2/2	12.9 ppm	7.20	●●●	●●●	●●●	1.0000	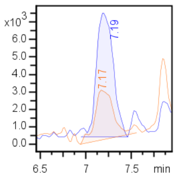
Salidroside	299.1136	-0.83	0.01	2/2	13.3 ppm	5.50	●●●	●●●	●●●	0.9945	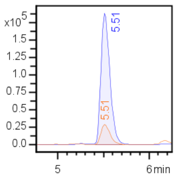
Verbascoside	623.1981	5.32	-0.03	2/2	0.1 ppm	8.60	●●●	●●●	●●●	0.9828	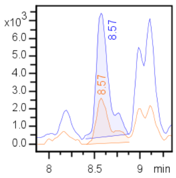

Analysis: Sample_70_17_1_1086

Creation Date	2025-03-17 23:16	Sample Type	Sample
Method	PhGs (28)	Mass Calib. Date	
Station Name	impact_II-10436	Operator	Demo User
Instrument	impact II	Instrument SN	1825265.10436
Data Path	D:\Data\Zhanpeis_Issayev\PhGs\Sample_70_17_1_1086.d		

Screening Results

Analyte	m/z exp.	Δ m/z [ppm]	Δ RT [min]	Mand Ions	Quantity	RT theo [min]	m/z Score	RT Score	Ions Score	R²	Chromatogram
Acetylacteoside ✍	665.2087	-3.54	-0.04	2/2	0.3 ppm	9.70	●●●	●●●	●●●	0.9919	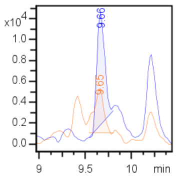
Echinacoside	785.2510	-2.35	-0.05	2/2	144.6 ppm	7.20	●●●	●●●	●●●	1.0000	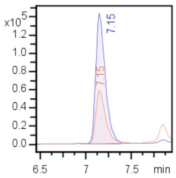
Salidroside	299.1136	-1.87	0.01	2/2	18.5 ppm	5.50	●●●	●●●	●●●	0.9945	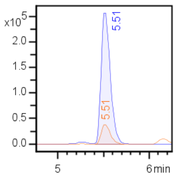
Tubuloside ✍	827.2615	-3.72	-0.04	2/2	0.3 ppm	8.40	●●●	●●●	●●●	0.9859	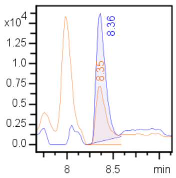
Verbascoside	623.1981	-1.39	-0.05	2/2	0.8 ppm	8.60	●●●	●●●	●●●	0.9828	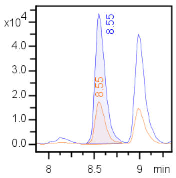

Analysis: Sample_77_18_1_1087

Creation Date	2025-03-17 23:44	Sample Type	Sample
Method	PhGs (28)	Mass Calib. Date	
Station Name	impact_II-10436	Operator	Demo User
Instrument	impact II	Instrument SN	1825265.10436
Data Path	D:\Data\Zhanpeis_Issayev\PhGs\Sample_77_18_1_1087.d		

Screening Results

Analyte	m/z exp.	Δ m/z [ppm]	Δ RT [min]	Mand Ions	Quantity	RT theo [min]	m/z Score	RT Score	Ions Score	R²	Chromatogram
Acetylacteoside	665.2087	-1.43	-0.05	2/2	2.3 ppm	9.70	●●●	●●●	●●●	0.9919	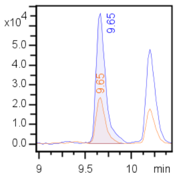
Echinacoside	785.2510	-1.58	-0.07	2/2	1641.6 ppm	7.20	●●●	●●●	●●●	1.0000	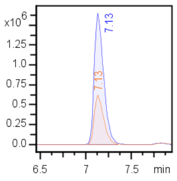
Salidroside	299.1136	-0.40	0.01	2/2	9.6 ppm	5.50	●●●	●●●	●●●	0.9945	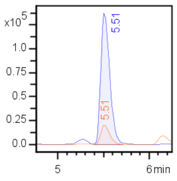
Tubuloside	827.2615	-1.52	-0.05	2/2	3.7 ppm	8.40	●●●	●●●	●●●	0.9859	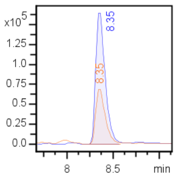
Verbascoside	623.1981	-1.57	-0.06	2/2	9.2 ppm	8.60	●●●	●●●	●●●	0.9828	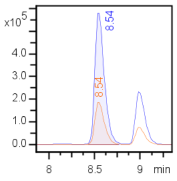

Analysis: Sample_15_2_1_1226

Creation Date	2025-05-14 12:44	Sample Type	Sample
Method	PhGs (28)	Mass Calib. Date	
Station Name	impact_II-10436	Operator	Demo User
Instrument	impact II	Instrument SN	1825265.10436
Data Path	D:\Data\Zhanpeis_Issayev\PhGs\28.04.2025\Sample_15_2_1_1226.d		

Screening Results

Analyte	m/z exp.	Δ m/z [ppm]	Δ RT [min]	Mand Ions	Quantity	RT theo [min]	m/z Score	RT Score	Ions Score	R^2	Chromatogram
Acetylacteoside ✍	665.2087	0.02	-0.12	2/2	20.3 ppm	9.70	●●●	●●●	●●●	0.9919	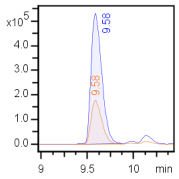
Echinacoside	785.2510	7.18	-0.16	2/2	10175.1 ppm	7.20	●●●	●●●	●●●	1.0000	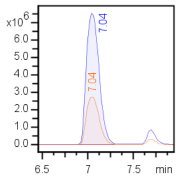
Salidroside	299.1136	54.46	-0.14	2/2	4.1 ppm	5.50	●●●	●●●	●●●	0.9945	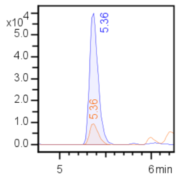

Analysis: Sample_17_3_1_1227

Creation Date	2025-05-14 13:12	Sample Type	Sample
Method	PhGs (28)	Mass Calib. Date	
Station Name	impact_II-10436	Operator	Demo User
Instrument	impact II	Instrument SN	1825265.10436
Data Path	D:\Data\Zhanpeis_Issayev\PhGs\28.04.2025\Sample_17_3_1_1227.d		

Screening Results

Analyte	m/z exp. / RT theo [min]	Δ m/z [ppm] / m/z Score	Δ RT [min] / RT Score	Mand Ions / Ions Score	Quantity / R^2	Chromatogram
Acetylacteoside ✍	665.2087	52.51	-0.11	2/2	10.5 ppm	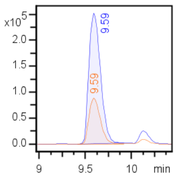
	9.70	●●●	●●●	●●●	0.9919	
Echinacoside ✍	785.2510	7.68	-0.16	2/2	10937.6 ppm	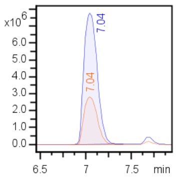
	7.20	●●●	●●●	●●●	1.0000	
Salidroside ✍	299.1136	55.29	-0.14	2/2	5.4 ppm	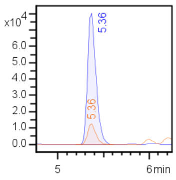
	5.50	●●●	●●●	●●●	0.9945	

Analysis: Sample_40_4_1_1228

Creation Date	2025-05-14 13:40	Sample Type	Sample
Method	PhGs (28)	Mass Calib. Date	
Station Name	impact_II-10436	Operator	Demo User
Instrument	impact II	Instrument SN	1825265.10436
Data Path	D:\Data\Zhanpeis_Issayev\PhGs\28.04.2025\Sample_40_4_1_1228.d		

Screening Results

Analyte	m/z exp.	Δ m/z [ppm]	Δ RT [min]	Mand Ions	Quantity	RT theo [min]	m/z Score	RT Score	Ions Score	R^2	Chromatogram
Acetylacteoside ✍	665.2087	-2.22	-0.07	2/2	2.0 ppm	9.70	●●●	●●●	●●●	0.9919	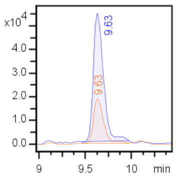
Echinacoside ✍	785.2510	51.34	-0.17	2/2	408.7 ppm	7.20	●●●	●●●	●●●	1.0000	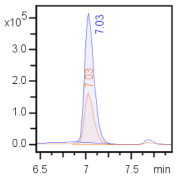
Salidroside ✍	299.1136	54.22	-0.15	2/2	6.2 ppm	5.50	●●●	●●●	●●●	0.9945	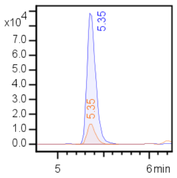
