# Supplementary material for: Stress-Induced Secondary Metabolite Profiling in Cistanche deserticola Callus Cultures: Insights from GC-MS and HPLC-MS Analysis
Source: Int J Mol Sci. 2025 Jun 25;26(13):6091. doi: 10.3390/ijms26136091 (PMC12250269; doi:10.3390/ijms26136091)
Supplement: Supplementary file 1 [file ijms-26-06091-s001.zip › Supplementary materials S9_qTOF_UHPLC-MS_Data/qTOF_UHPLC-MS_Results/Sample_40.pdf]

AOE "Nazarbayev University"
Office of Research Core Facilities and HPC
Address: 53, Kabanbay Batyr ave.,
Astana, 010000, Republic of Kazakhstan

+7 (7172) 70 64 78
provost.cf@nu.edu.kz

| | | | |
|---|---|---|---|
| Analysis Name | D:\Data\Sample_40_7_1_1074.d | Acquisition Date | 3/17/2025 1:26:19 PM |
| Method | PhGs.m | Operator | Demo User |
| Sample Name | Sample_40 | Instrument | impact II |
| Comment | | | |

**Acquisition Parameter**

| | | | | | |
|---|---|---|---|---|---|
| Source Type | ESI | Ion Polarity | Negative | Set Nebulizer | 3.0 Bar |
| Focus | Active | | | Set Dry Heater | 200 °C |
| Scan Begin | 50 m/z | Set Capillary | 3000 V | Set Dry Gas | 7.0 l/min |
| Scan End | 1300 m/z | Set End Plate Offset | -500 V | Set Divert Valve | Source |

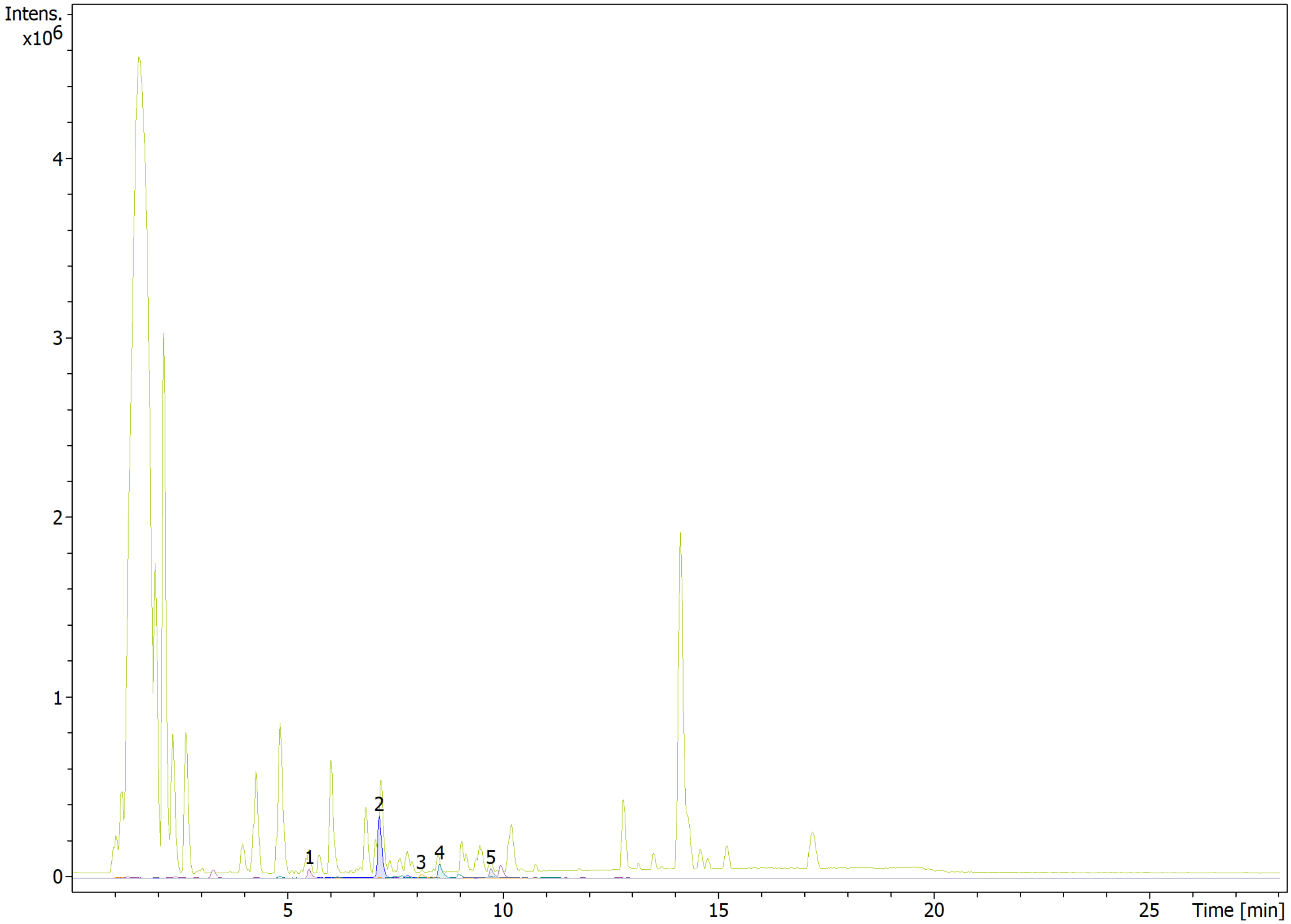


The results of the analyses conducted in our laboratory are intended solely for scientific research purposes. The laboratory is not responsible for the use of this data in legal matters.

AOE "Nazarbayev University"
Office of Research Core Facilities and HPC
Address: 53, Kabanbay Batyr ave.,
Astana, 010000, Republic of Kazakhstan

+7 (7172) 70 64 78
provost.cf@nu.edu.kz

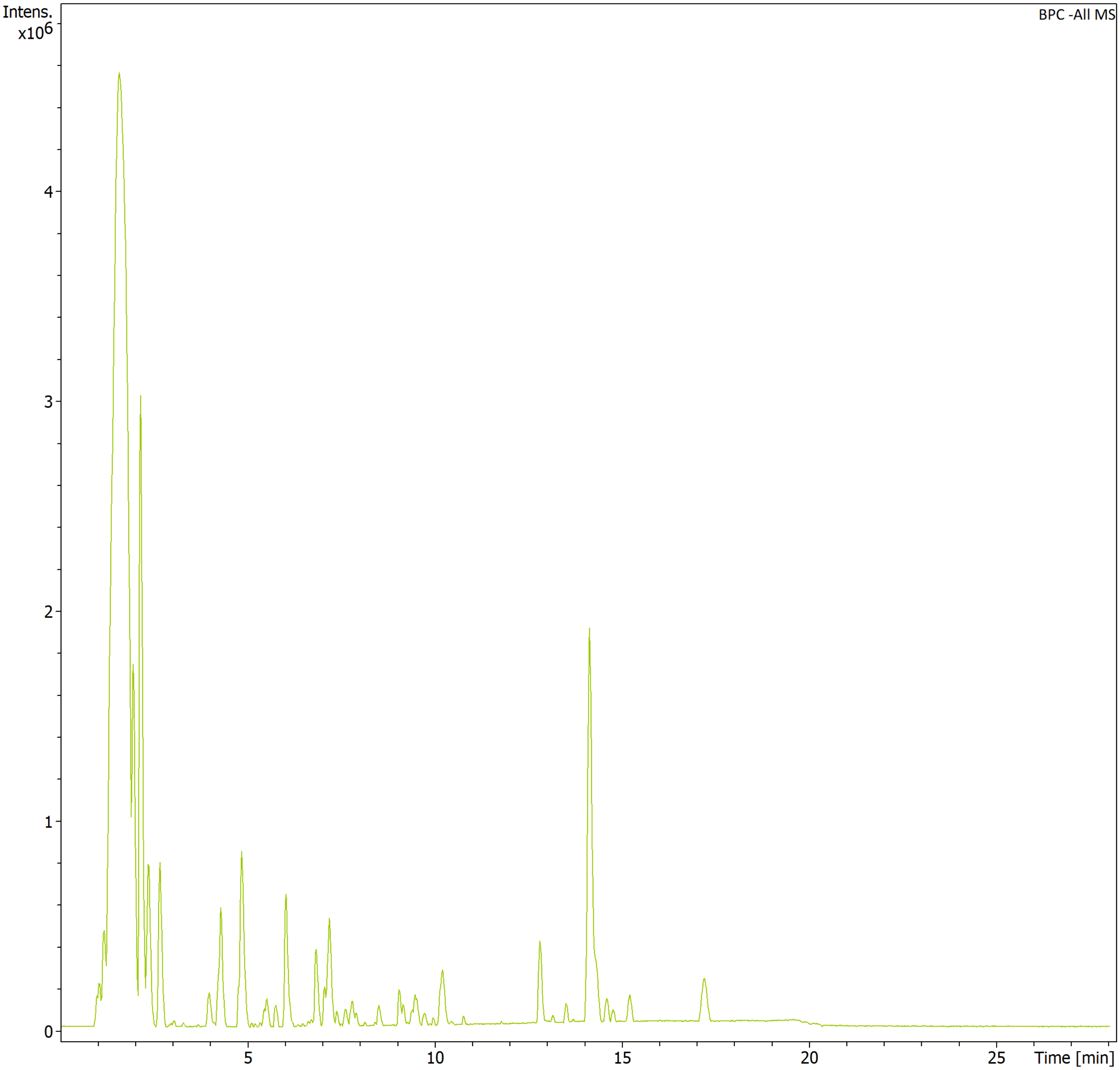


The results of the analyses conducted in our laboratory are intended solely for scientific research purposes. The laboratory is not responsible for the use of this data in legal matters.

AOE "Nazarbayev University"
Office of Research Core Facilities and HPC
Address: 53, Kabanbay Batyr ave.,
Astana, 010000, Republic of Kazakhstan

+7 (7172) 70 64 78
provost.cf@nu.edu.kz

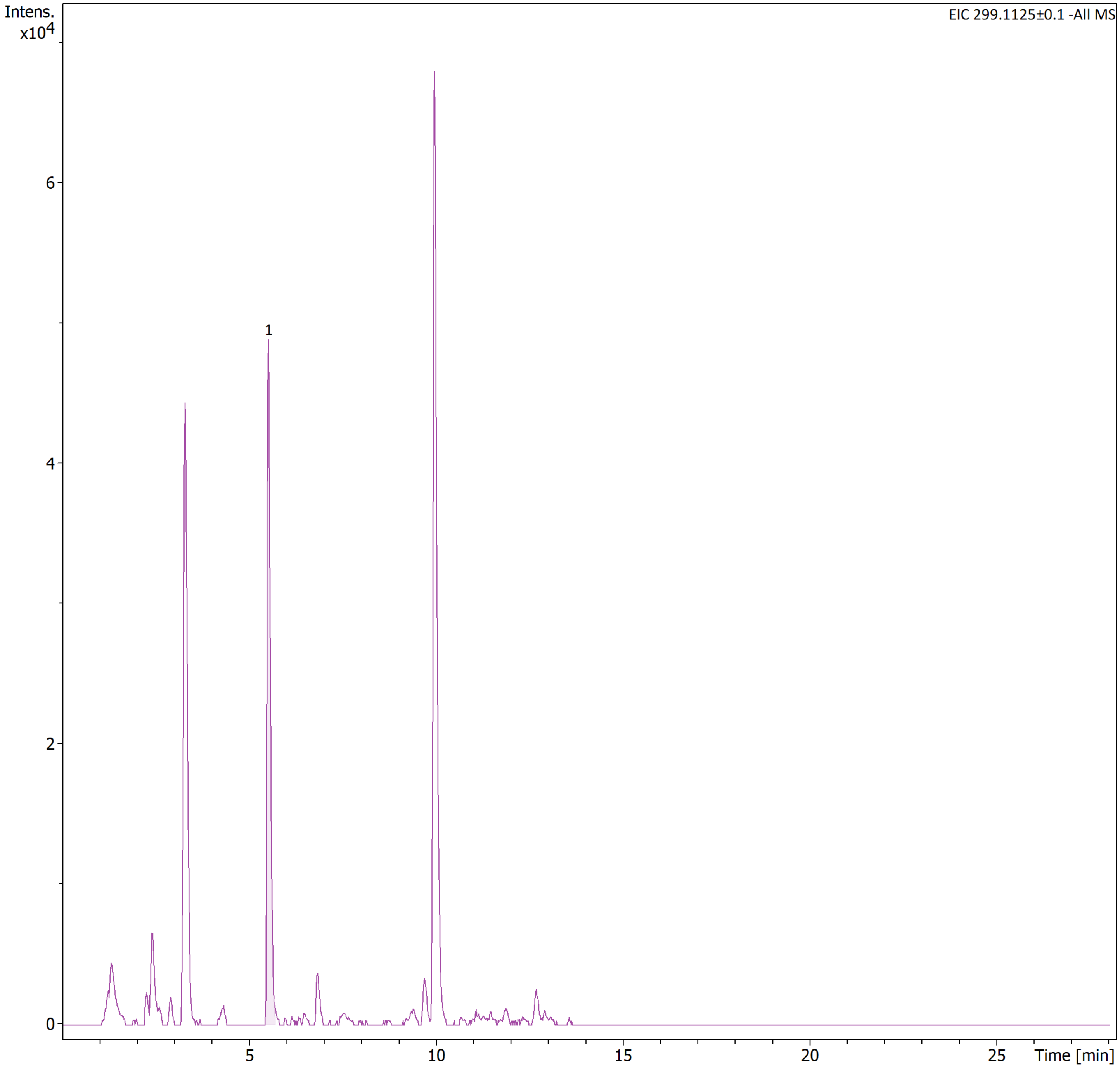


The results of the analyses conducted in our laboratory are intended solely for scientific research purposes. The laboratory is not responsible for the use of this data in legal matters.

AOE "Nazarbayev University"
Office of Research Core Facilities and HPC
Address: 53, Kabanbay Batyr ave.,
Astana, 010000, Republic of Kazakhstan

+7 (7172) 70 64 78
provost.cf@nu.edu.kz

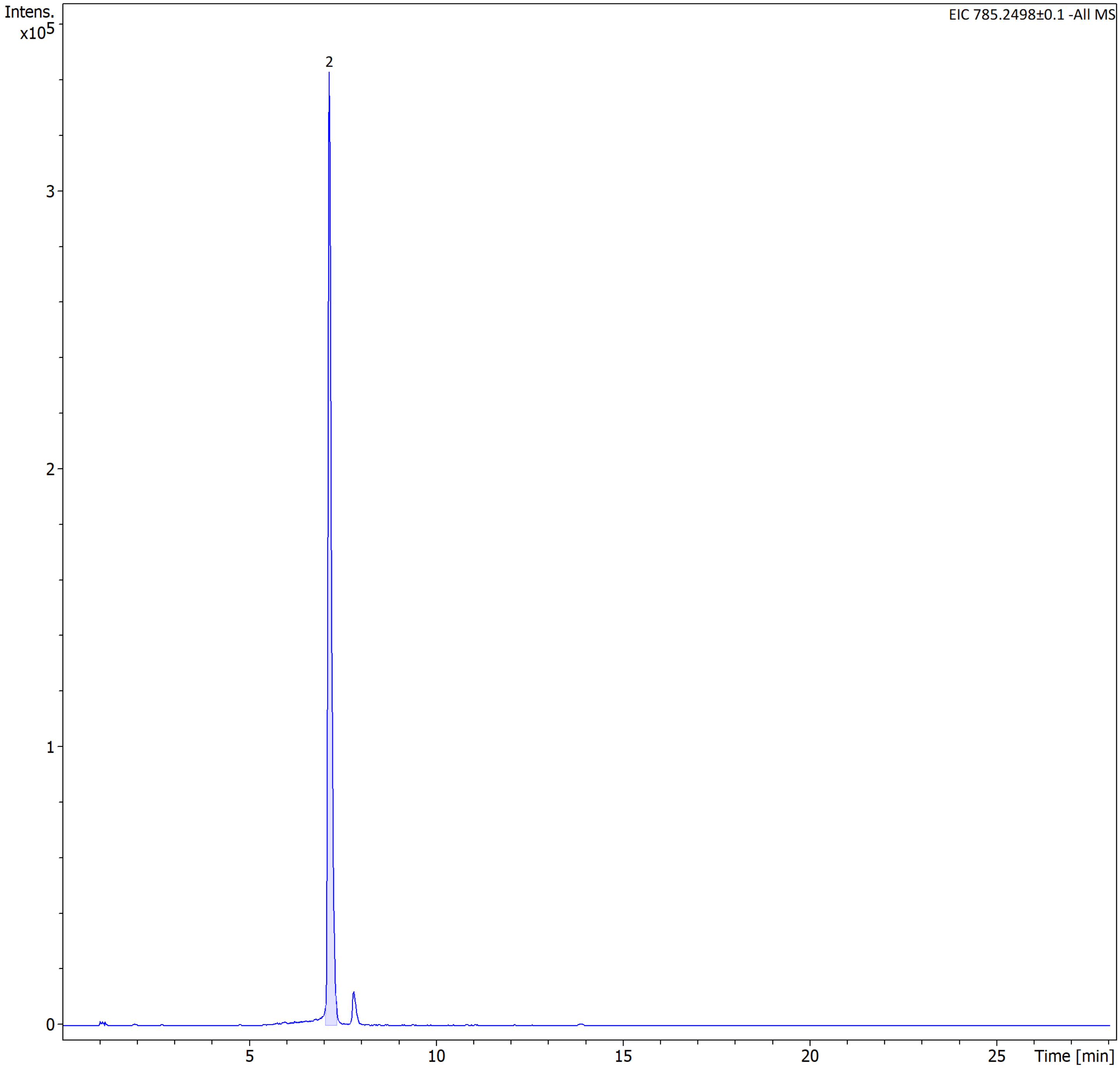


The results of the analyses conducted in our laboratory are intended solely for scientific research purposes. The laboratory is not responsible for the use of this data in legal matters.

AOE "Nazarbayev University"
Office of Research Core Facilities and HPC
Address: 53, Kabanbay Batyr ave.,
Astana, 010000, Republic of Kazakhstan

+7 (7172) 70 64 78
provost.cf@nu.edu.kz

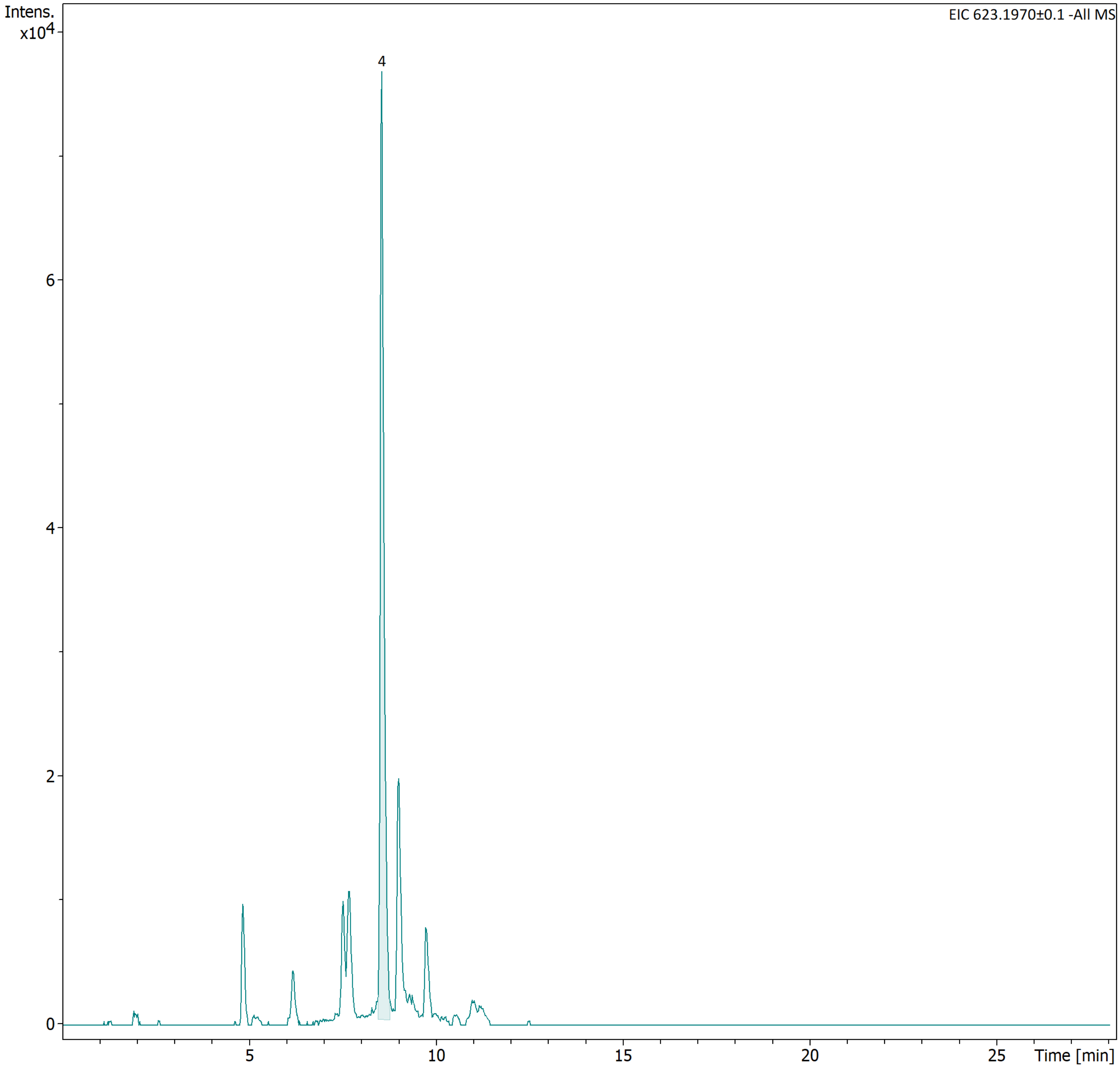


The results of the analyses conducted in our laboratory are intended solely for scientific research purposes. The laboratory is not responsible for the use of this data in legal matters.

AOE "Nazarbayev University"
Office of Research Core Facilities and HPC
Address: 53, Kabanbay Batyr ave.,
Astana, 010000, Republic of Kazakhstan

+7 (7172) 70 64 78
provost.cf@nu.edu.kz

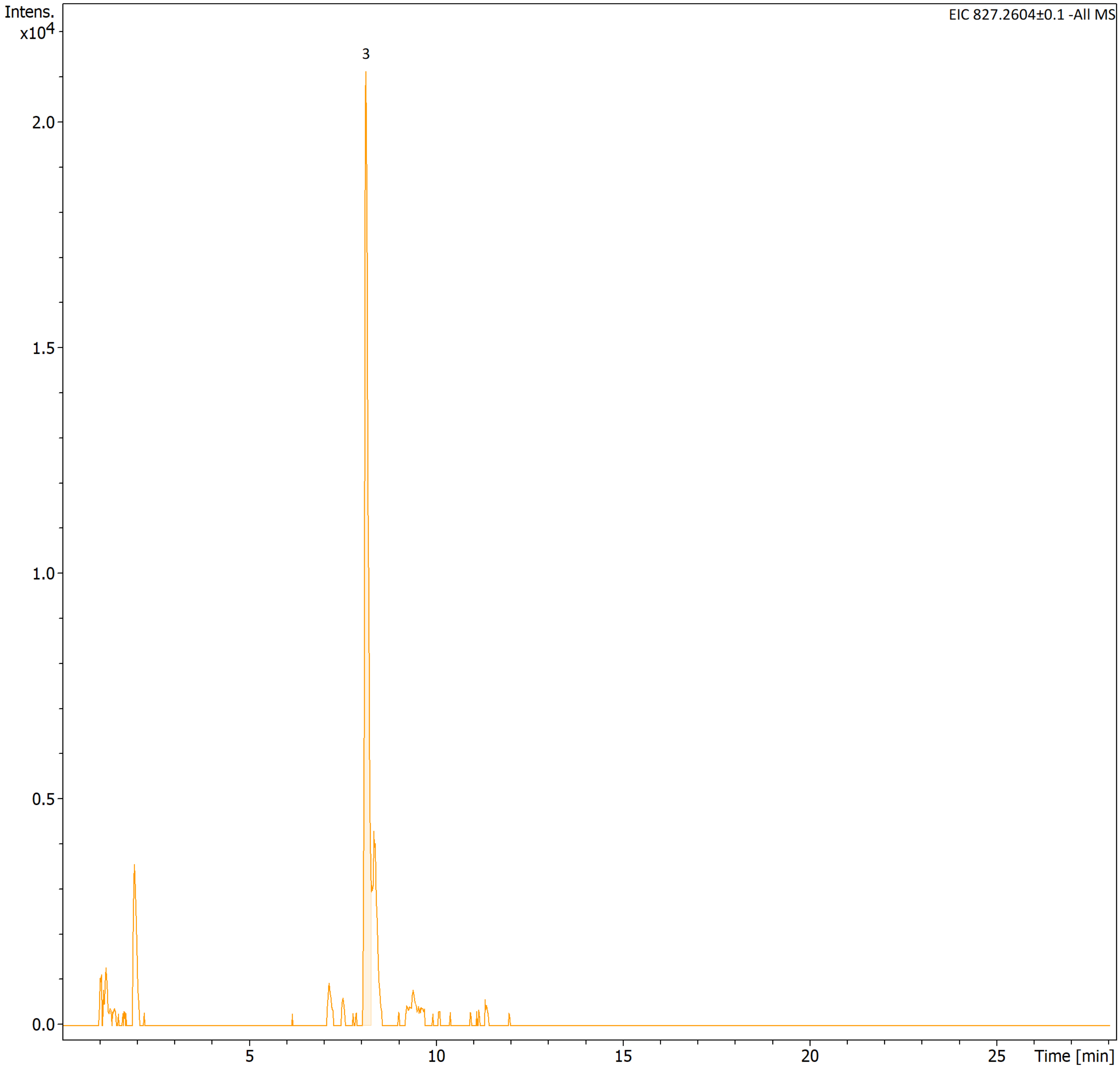


The results of the analyses conducted in our laboratory are intended solely for scientific research purposes. The laboratory is not responsible for the use of this data in legal matters.

AOE "Nazarbayev University"
Office of Research Core Facilities and HPC
Address: 53, Kabanbay Batyr ave.,
Astana, 010000, Republic of Kazakhstan

+7 (7172) 70 64 78
provost.cf@nu.edu.kz

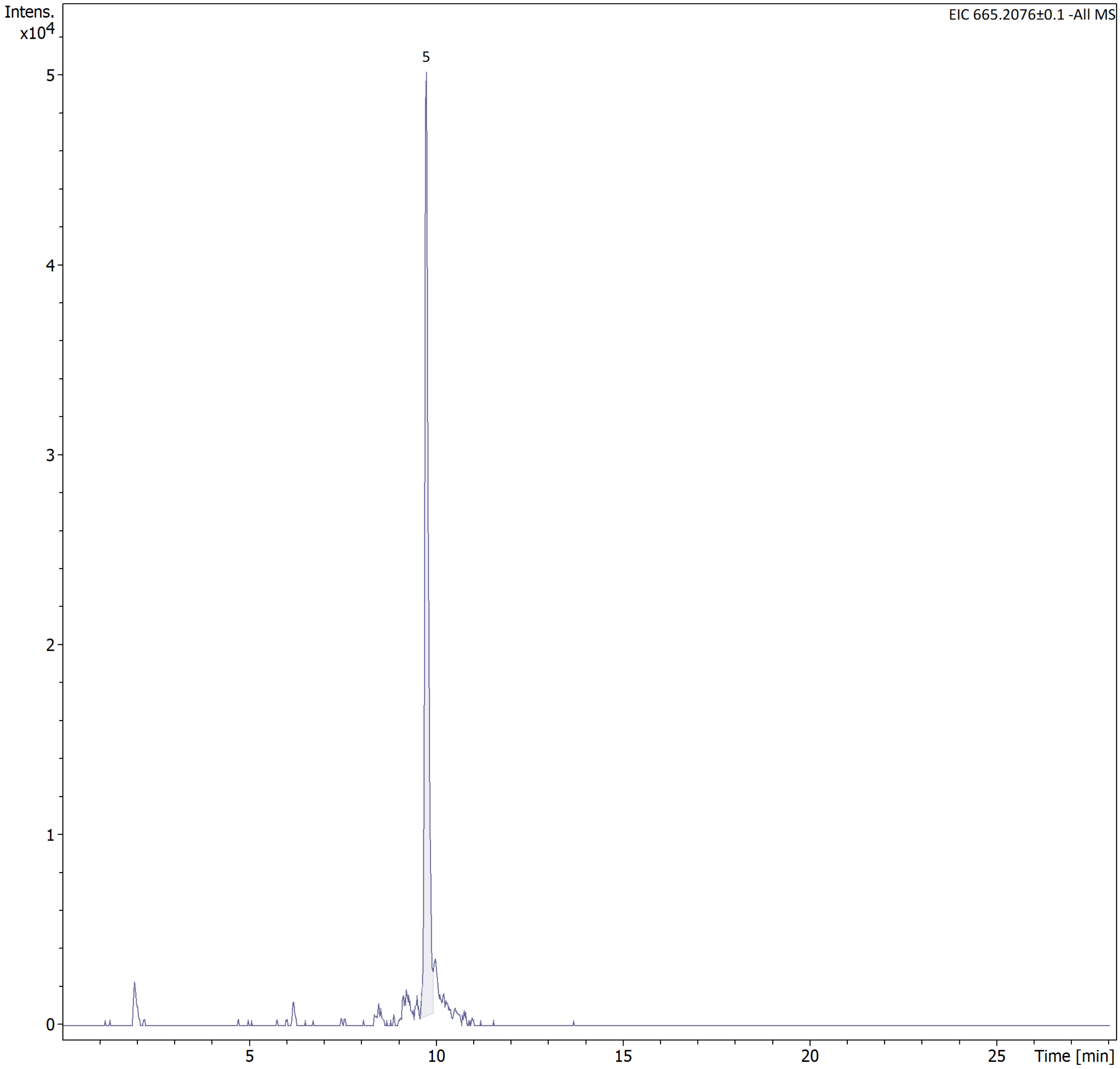


The results of the analyses conducted in our laboratory are intended solely for scientific research purposes. The laboratory is not responsible for the use of this data in legal matters.

AOE "Nazarbayev University"
Office of Research Core Facilities and HPC
Address: 53, Kabanbay Batyr ave.,
Astana, 010000, Republic of Kazakhstan

+7 (7172) 70 64 78
provost.cf@nu.edu.kz

NAZARBAYEV UNIVERSITY

| # | RT [min] | Area | Int. Type | I | S/N | Trace | Max. m/z | FWHM [min] |
|---|---|---|---|---|---|---|---|---|
| 1 | 5.5 | 302968 | Chromatogram | 48838 | 126.2 | EIC 299.1125±0.1 -All MS | 345.1193 | 0.1 |
| 2 | 7.1 | 2245369 | Chromatogram | 342921 | 1205.4 | EIC 785.2498±0.1 -All MS | 121.0297 | 0.1 |
| 3 | 8.1 | 146727 | Chromatogram | 21292 | 60.7 | EIC 827.2604±0.1 -All MS | 307.1401 | 0.1 |
| 4 | 8.5 | 535020 | Chromatogram | 76864 | 152.5 | EIC 623.1970±0.1 -All MS | 623.1972 | 0.1 |
| 5 | 9.7 | 345660 | Chromatogram | 50457 | 101.0 | EIC 665.2076±0.1 -All MS | 621.2180 | 0.1 |

**Cmpd 1, 5.5 min**

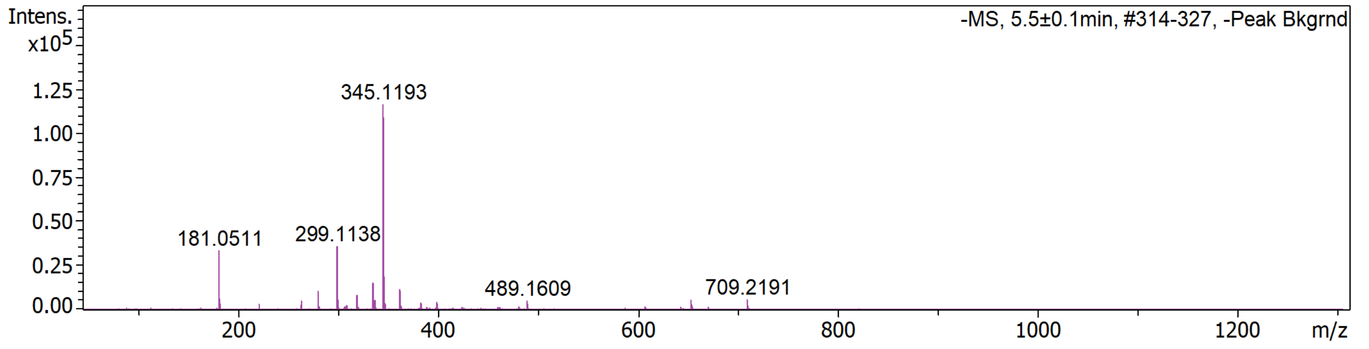


**Cmpd 2, 7.1 min**

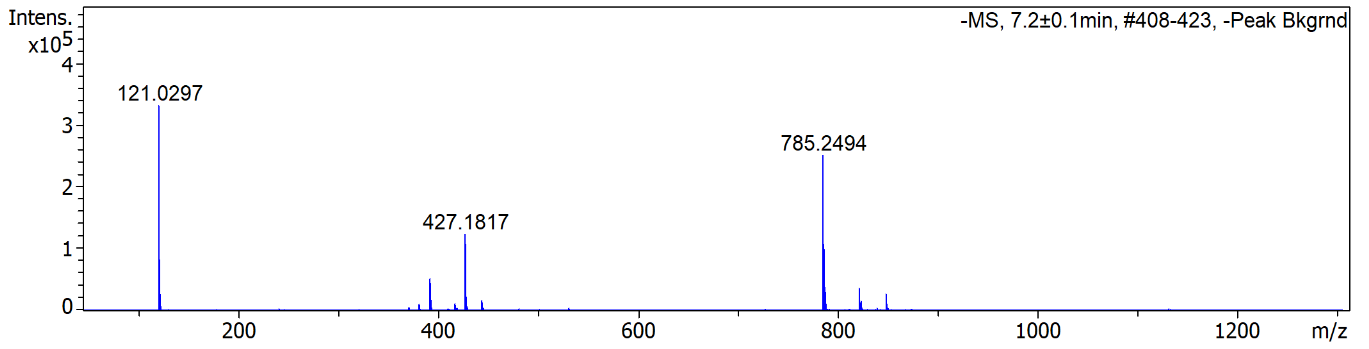

AOE "Nazarbayev University"
Office of Research Core Facilities and HPC
Address: 53, Kabanbay Batyr ave.,
Astana, 010000, Republic of Kazakhstan

+7 (7172) 70 64 78
provost.cf@nu.edu.kz

**Cmpd 3, 8.1 min**

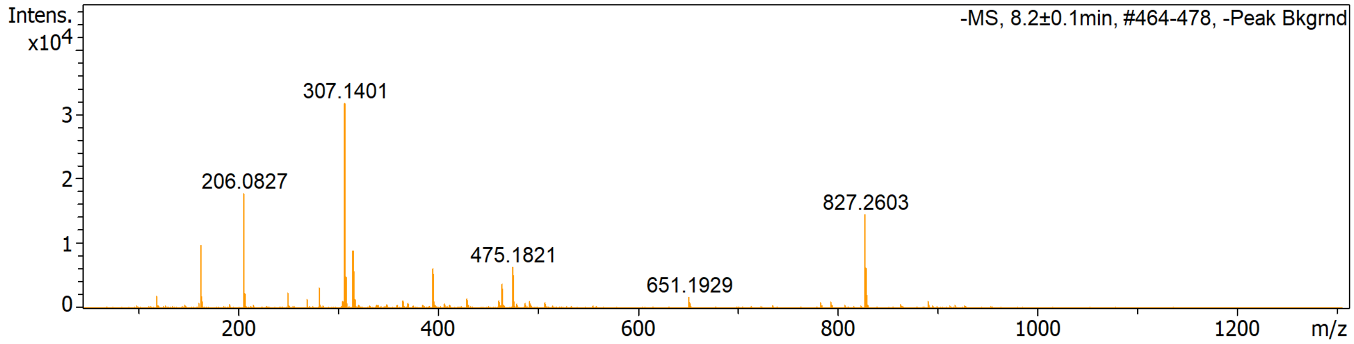


**Cmpd 4, 8.5 min**

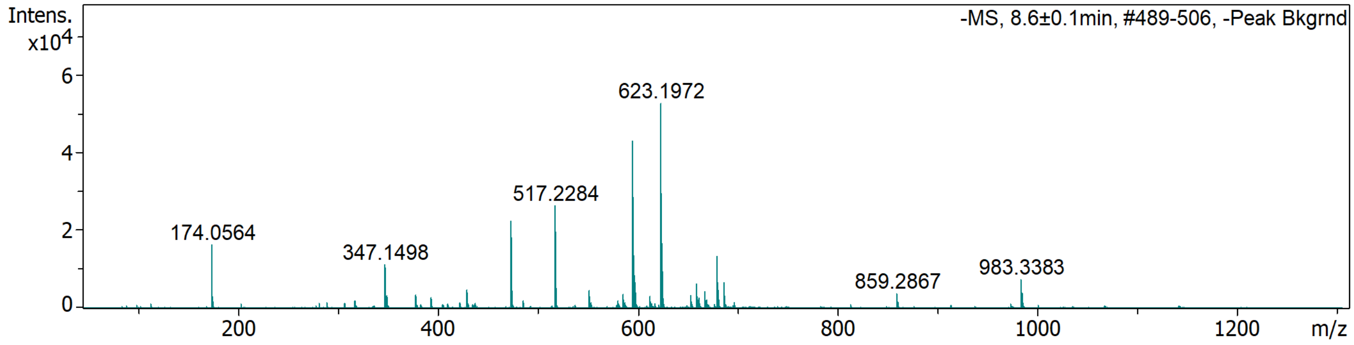


**Cmpd 5, 9.7 min**

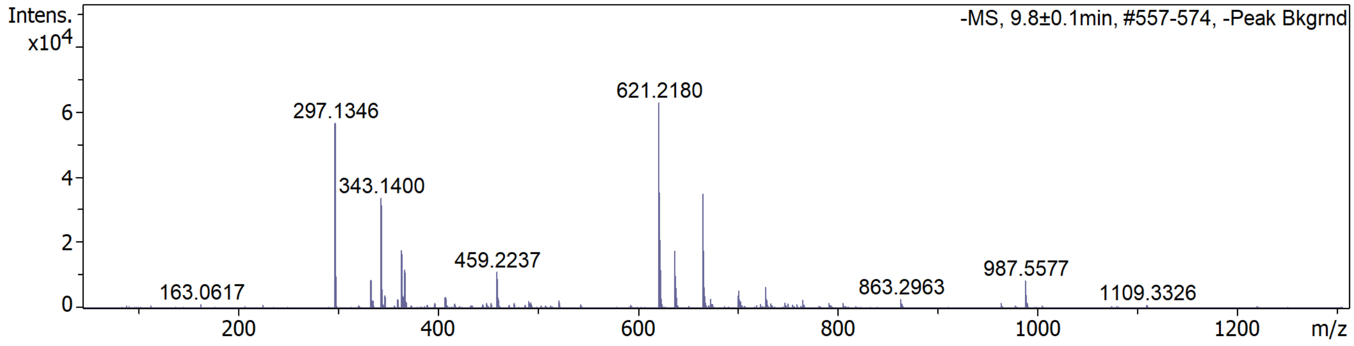


The results of the analyses conducted in our laboratory are intended solely for scientific research purposes. The laboratory is not responsible for the use of this data in legal matters.
