# Supplementary material for: Stress-Induced Secondary Metabolite Profiling in Cistanche deserticola Callus Cultures: Insights from GC-MS and HPLC-MS Analysis
Source: Int J Mol Sci. 2025 Jun 25;26(13):6091. doi: 10.3390/ijms26136091 (PMC12250269; doi:10.3390/ijms26136091)
Supplement: Supplementary file 1 [file ijms-26-06091-s001.zip › Supplementary materials S9_qTOF_UHPLC-MS_Data/qTOF_UHPLC-MS_Results/Sample_42.pdf]

AOE "Nazarbayev University"
Office of Research Core Facilities and HPC
Address: 53, Kabanbay Batyr ave.,
Astana, 010000, Republic of Kazakhstan

+7 (7172) 70 64 78
provost.cf@nu.edu.kz

Analysis Name	D:\Data\Zhanpeis_Issayev\PhGs\Sample_42_8_1_1075.d	Acquisition Date	3/17/2025 1:55:04 PM
Method	PhGs.m	Operator	Demo User
Sample Name	Sample_42	Instrument	impact II
Comment			

Acquisition Parameter

Source Type	ESI	Ion Polarity	Negative	Set Nebulizer	3.0 Bar
Focus	Active			Set Dry Heater	200 °C
Scan Begin	50 m/z	Set Capillary	3000 V	Set Dry Gas	7.0 l/min
Scan End	1300 m/z	Set End Plate Offset	-500 V	Set Divert Valve	Source

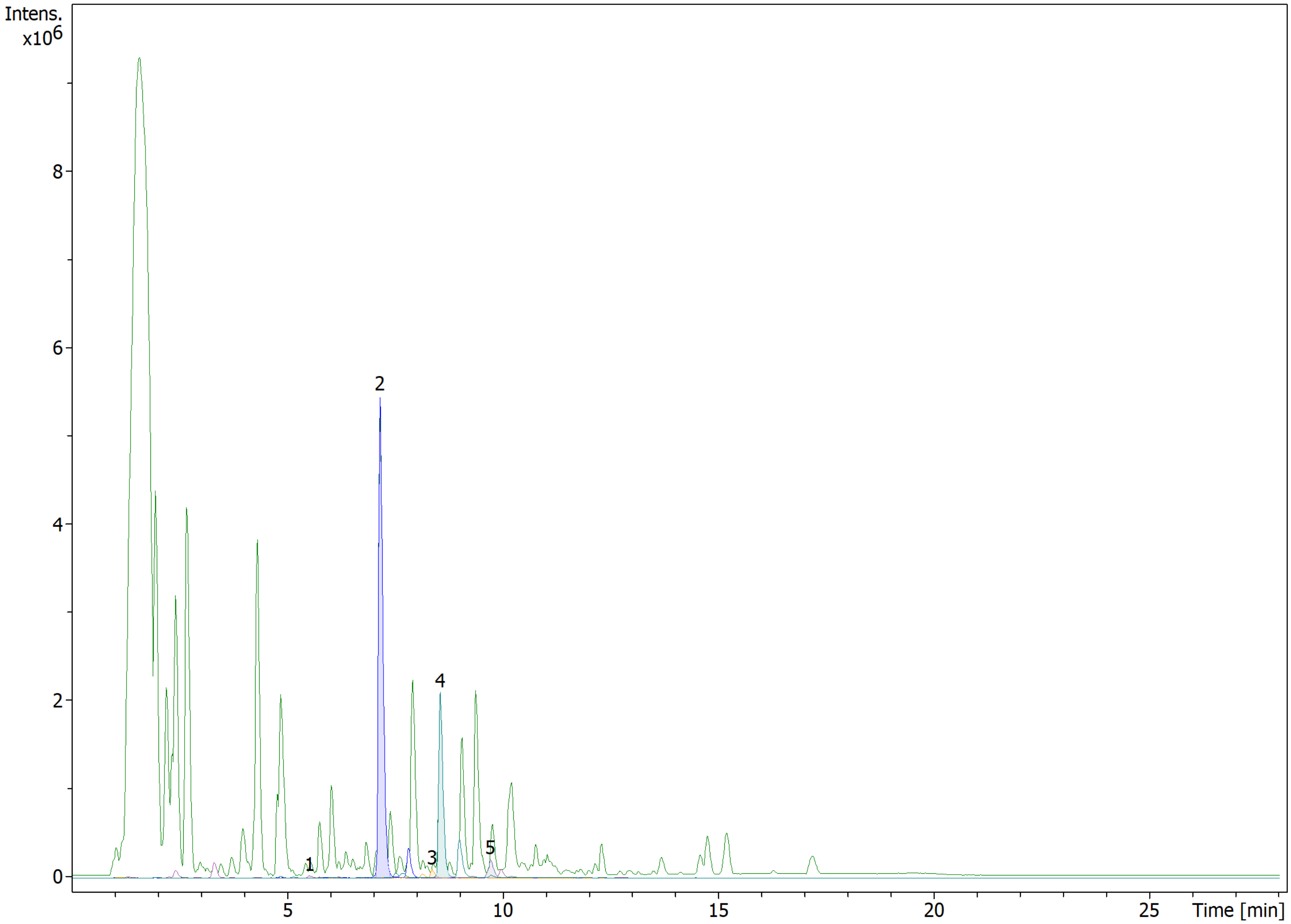

The results of the analyses conducted in our laboratory are intended solely for scientific research purposes. The laboratory is not responsible for the use of this data in legal matters.

AOE "Nazarbayev University"
Office of Research Core Facilities and HPC
Address: 53, Kabanbay Batyr ave.,
Astana, 010000, Republic of Kazakhstan

+7 (7172) 70 64 78
provost.cf@nu.edu.kz

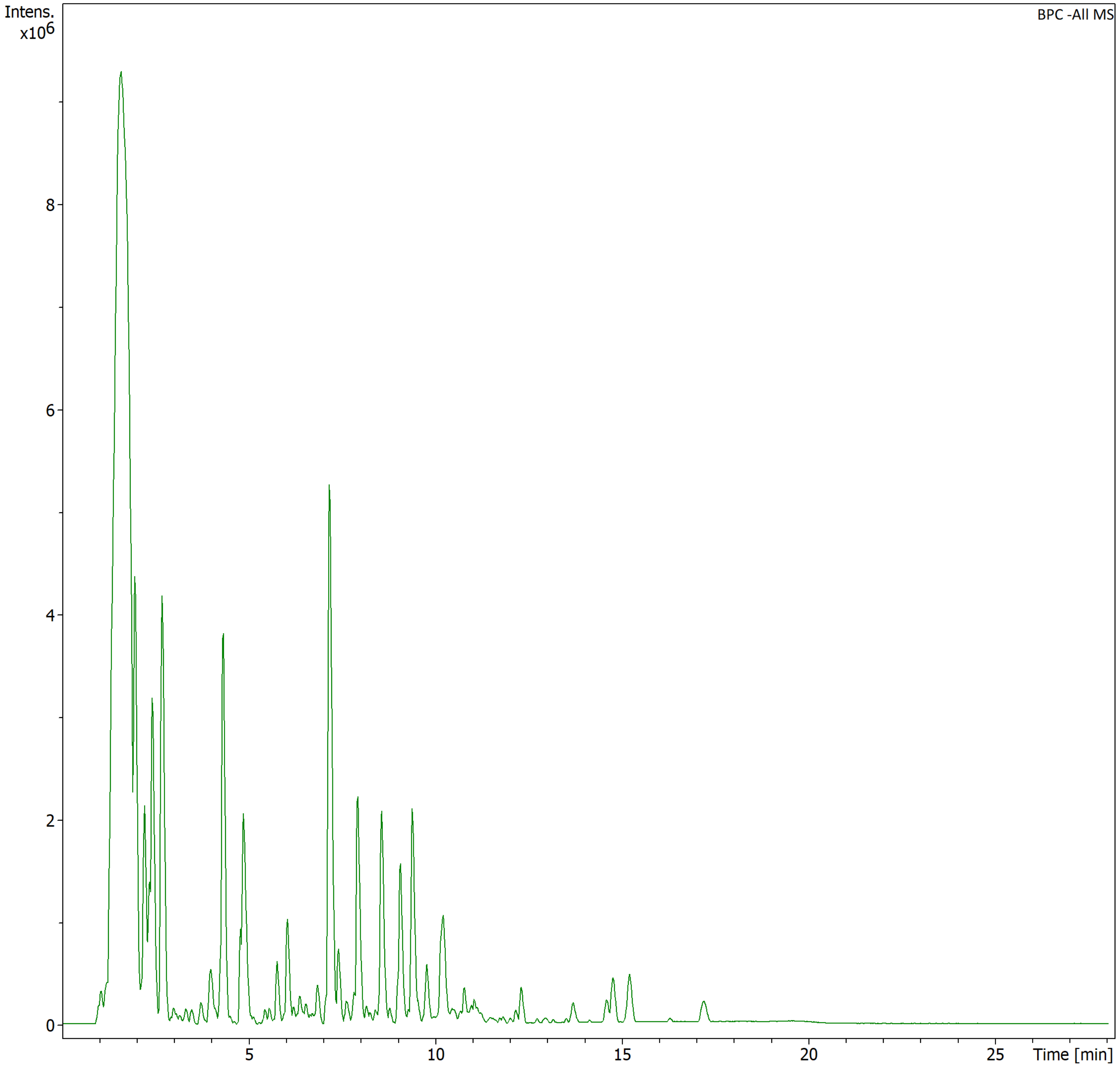

The results of the analyses conducted in our laboratory are intended solely for scientific research purposes. The laboratory is not responsible for the use of this data in legal matters.

AOE "Nazarbayev University"
Office of Research Core Facilities and HPC
Address: 53, Kabanbay Batyr ave.,
Astana, 010000, Republic of Kazakhstan

+7 (7172) 70 64 78
provost.cf@nu.edu.kz

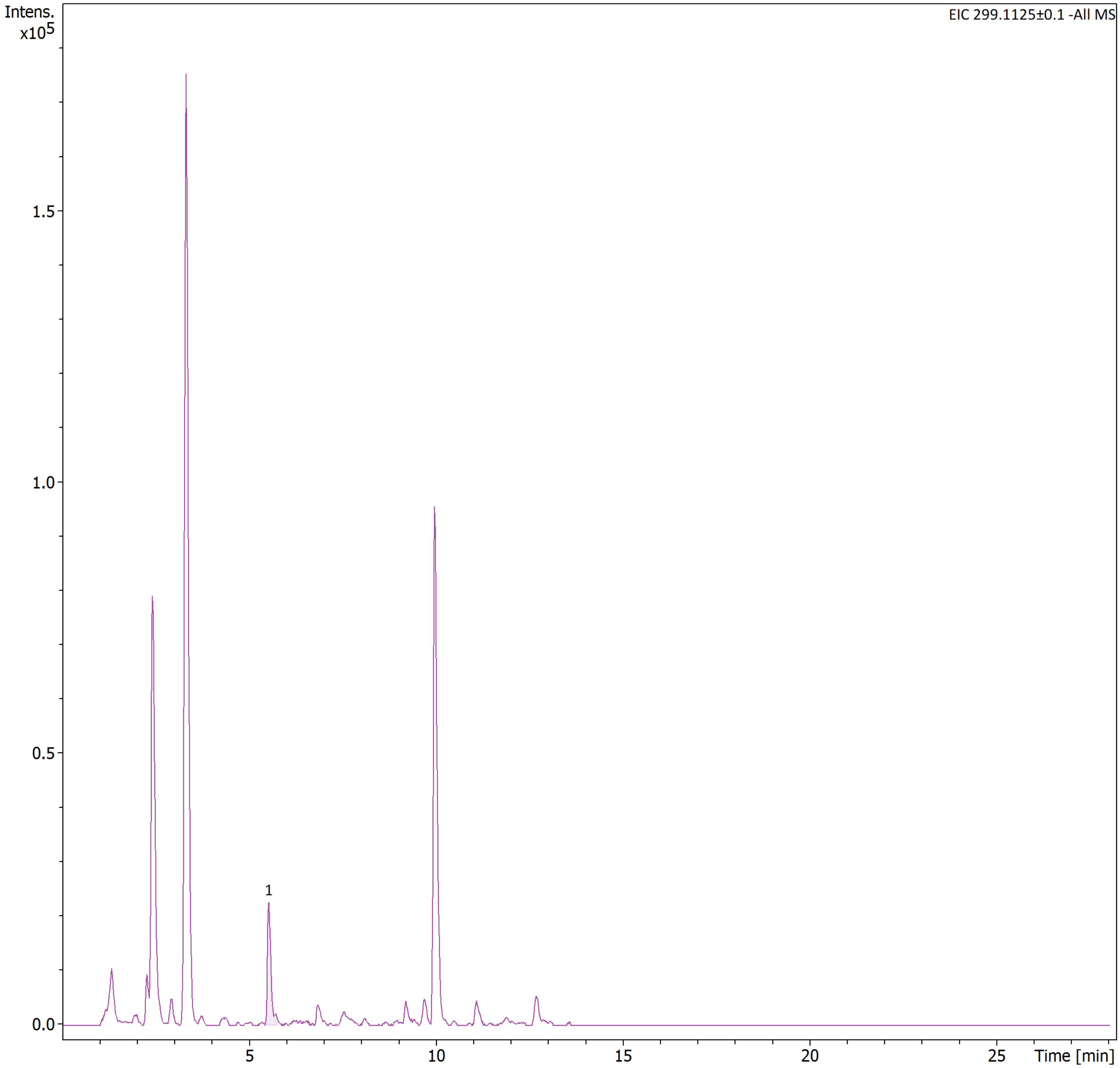

The results of the analyses conducted in our laboratory are intended solely for scientific research purposes. The laboratory is not responsible for the use of this data in legal matters.

NAZARBAYEV UNIVERSITY

AOE "Nazarbayev University"
Office of Research Core Facilities and HPC
Address: 53, Kabanbay Batyr ave.,
Astana, 010000, Republic of Kazakhstan

+7 (7172) 70 64 78
provost.cf@nu.edu.kz

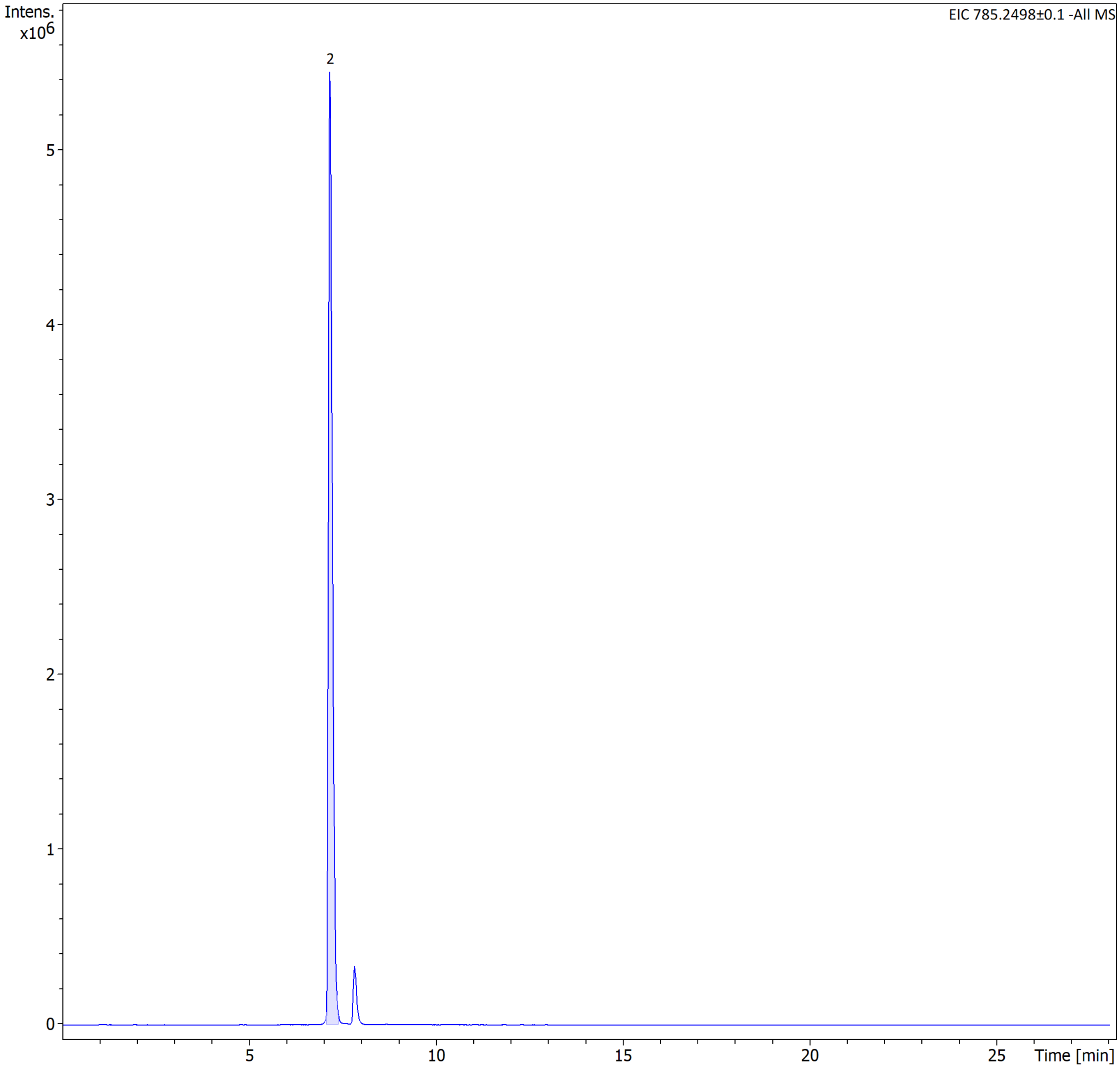

The results of the analyses conducted in our laboratory are intended solely for scientific research purposes. The laboratory is not responsible for the use of this data in legal matters.

AOE "Nazarbayev University"
Office of Research Core Facilities and HPC
Address: 53, Kabanbay Batyr ave.,
Astana, 010000, Republic of Kazakhstan

+7 (7172) 70 64 78
provost.cf@nu.edu.kz

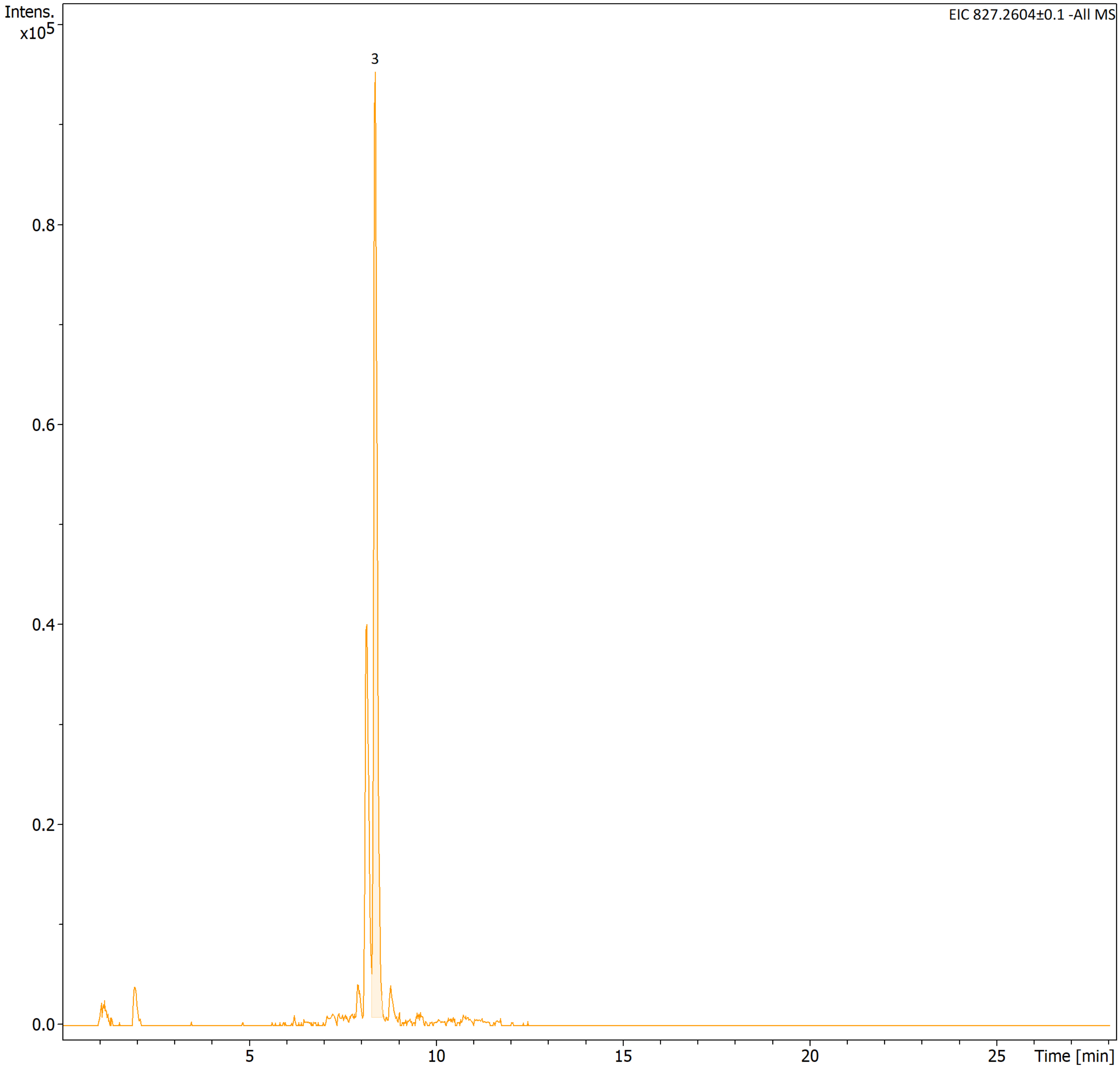

The results of the analyses conducted in our laboratory are intended solely for scientific research purposes. The laboratory is not responsible for the use of this data in legal matters.

AOE "Nazarbayev University"
Office of Research Core Facilities and HPC
Address: 53, Kabanbay Batyr ave.,
Astana, 010000, Republic of Kazakhstan

+7 (7172) 70 64 78
provost.cf@nu.edu.kz

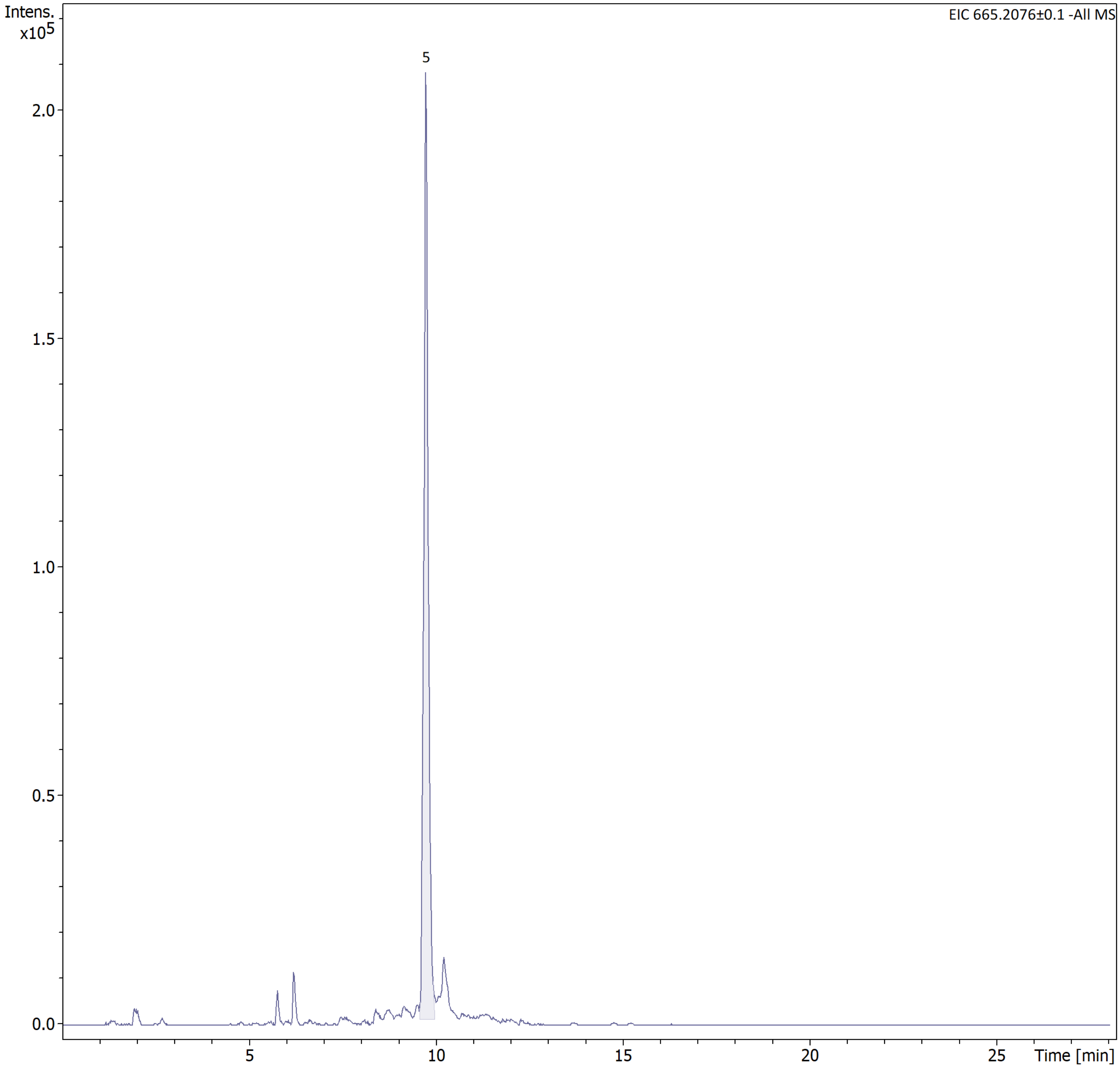

The results of the analyses conducted in our laboratory are intended solely for scientific research purposes. The laboratory is not responsible for the use of this data in legal matters.

AOE "Nazarbayev University"
Office of Research Core Facilities and HPC
Address: 53, Kabanbay Batyr ave.,
Astana, 010000, Republic of Kazakhstan

+7 (7172) 70 64 78
provost.cf@nu.edu.kz

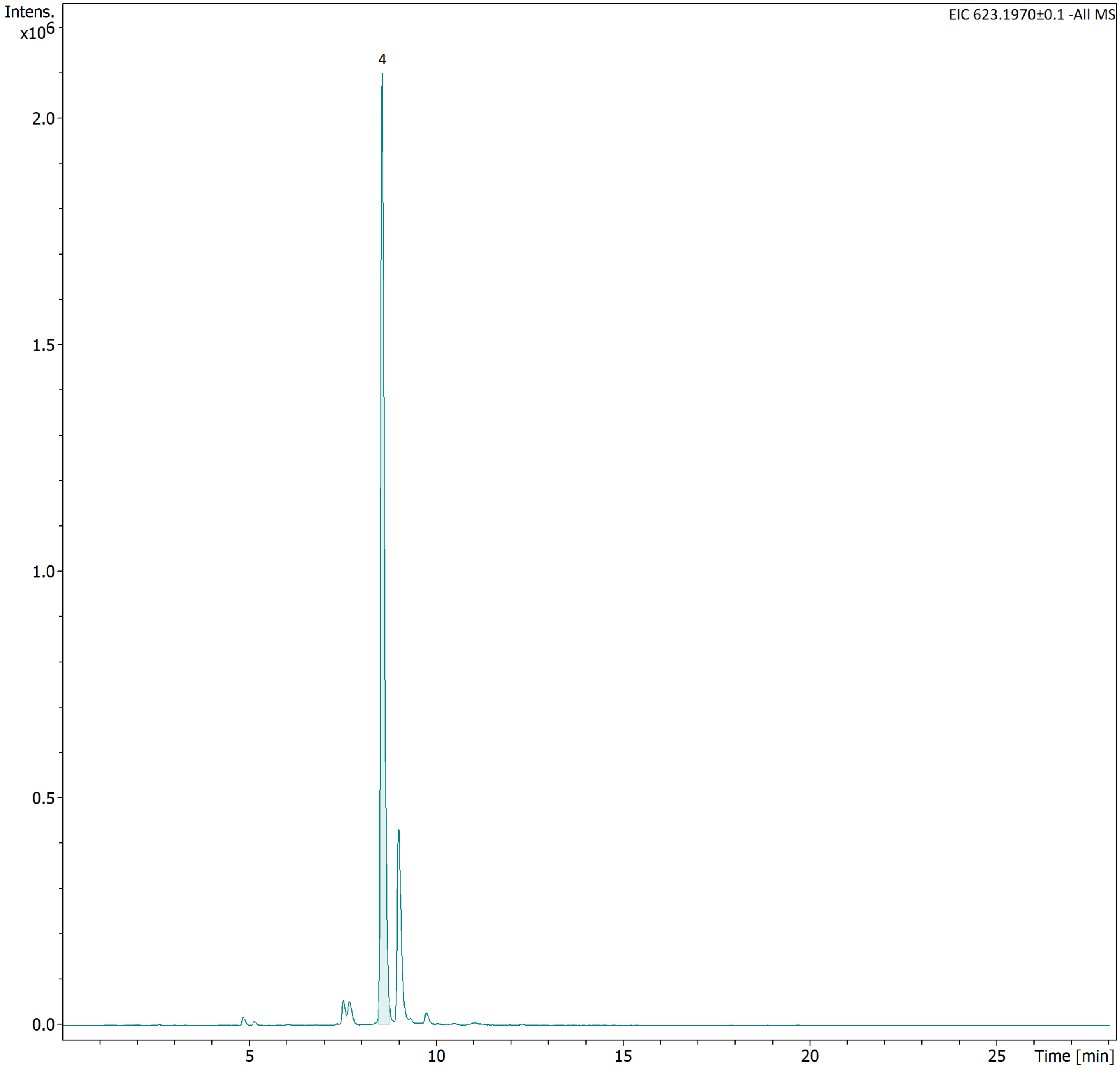

The results of the analyses conducted in our laboratory are intended solely for scientific research purposes. The laboratory is not responsible for the use of this data in legal matters.

AOE "Nazarbayev University"
Office of Research Core Facilities and HPC
Address: 53, Kabanbay Batyr ave.,
Astana, 010000, Republic of Kazakhstan

+7 (7172) 70 64 78
provost.cf@nu.edu.kz

#	RT [min]	Area	Int. Type	I	S/N	Trace	Max. m/z	FWHM [min]
1	5.5	144609	Chromatogram	22998	40.4	EIC 299.1125±0.1 -All MS	383.1917	0.1
2	7.2	39650020	Chromatogram	5464814	15502.8	EIC 785.2498±0.1 -All MS	785.2490	0.1
3	8.4	626881	Chromatogram	95612	192.5	EIC 827.2604±0.1 -All MS	565.1916	0.1
4	8.6	14651398	Chromatogram	2108498	2908.0	EIC 623.1970±0.1 -All MS	623.1964	0.1
5	9.7	1719952	Chromatogram	209375	321.3	EIC 665.2076±0.1 -All MS	637.2119	0.1

Cmpd 1, 5.5 min

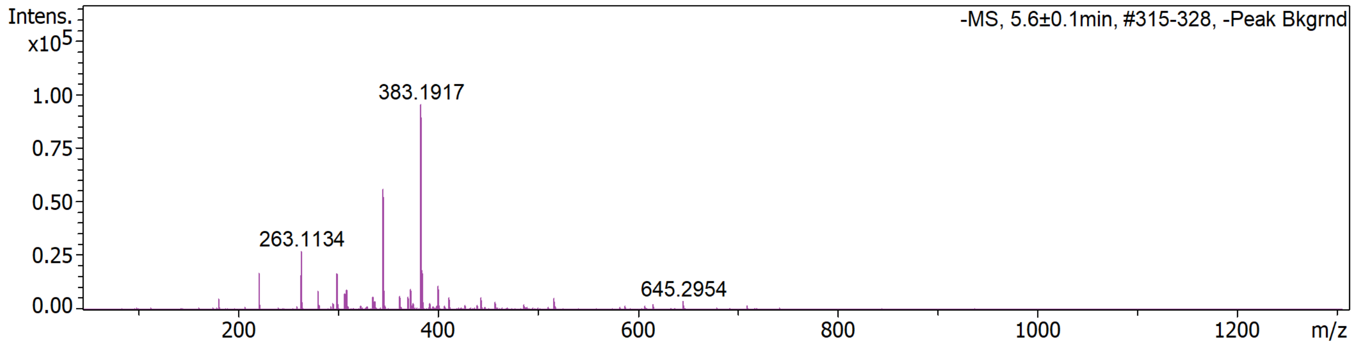

Cmpd 2, 7.2 min

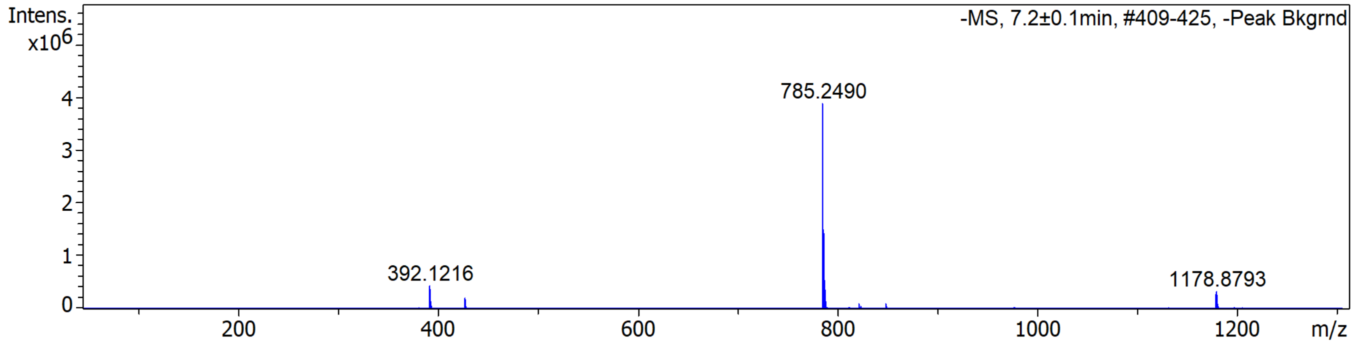

The results of the analyses conducted in our laboratory are intended solely for scientific research purposes. The laboratory is not responsible for the use of this data in legal matters.

AOE "Nazarbayev University"
Office of Research Core Facilities and HPC
Address: 53, Kabanbay Batyr ave.,
Astana, 010000, Republic of Kazakhstan

+7 (7172) 70 64 78
provost.cf@nu.edu.kz

Cmpd 3, 8.4 min

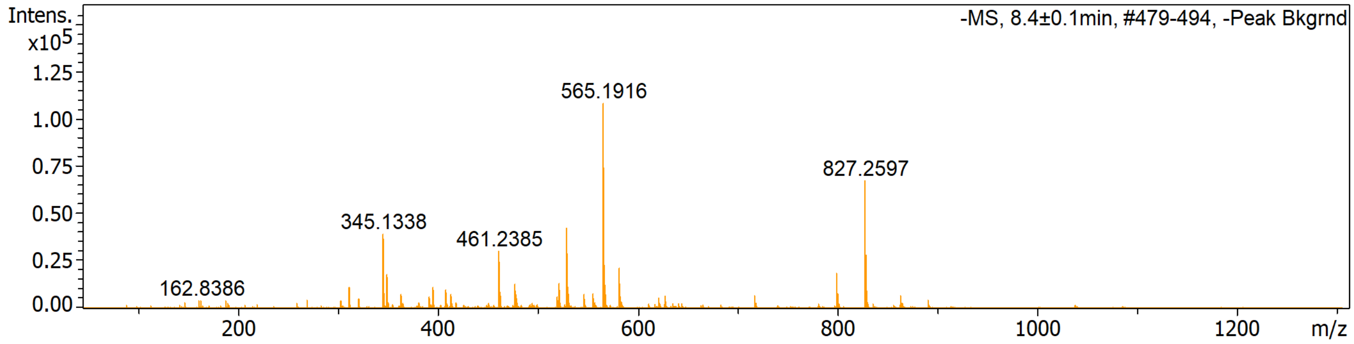

Cmpd 4, 8.6 min

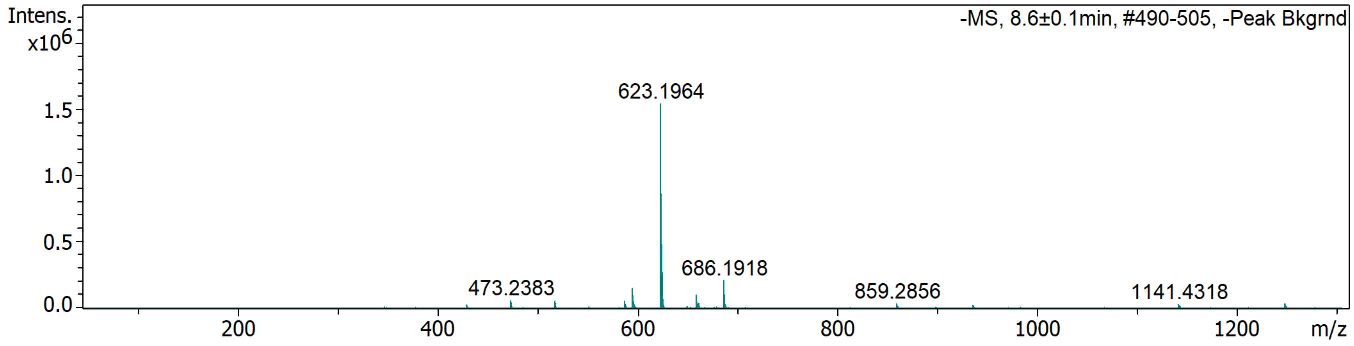

Cmpd 5, 9.7 min

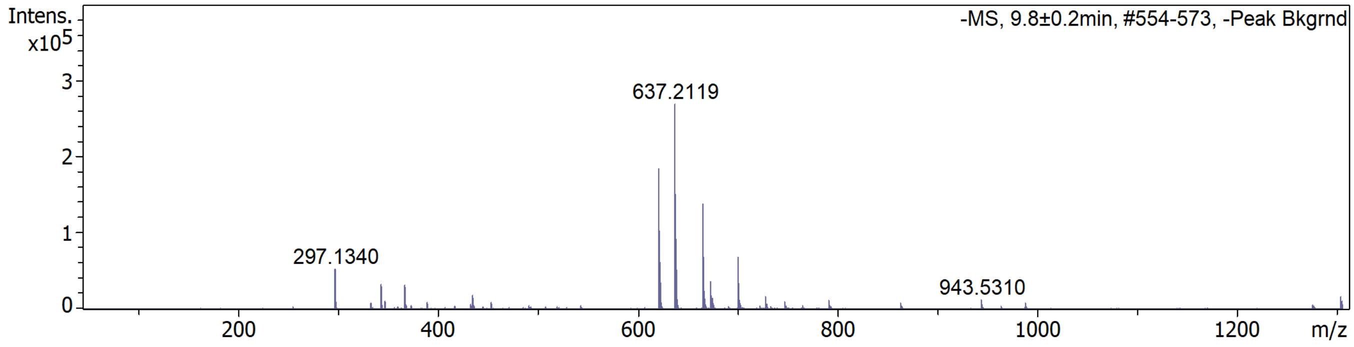

The results of the analyses conducted in our laboratory are intended solely for scientific research purposes. The laboratory is not responsible for the use of this data in legal matters.
