# Supplementary material for: Stress-Induced Secondary Metabolite Profiling in Cistanche deserticola Callus Cultures: Insights from GC-MS and HPLC-MS Analysis
Source: Int J Mol Sci. 2025 Jun 25;26(13):6091. doi: 10.3390/ijms26136091 (PMC12250269; doi:10.3390/ijms26136091)
Supplement: Supplementary file 1 [file ijms-26-06091-s001.zip › Supplementary materials S9_qTOF_UHPLC-MS_Data/qTOF_UHPLC-MS_Results/Sample_50.pdf]

AOE "Nazarbayev University"
Office of Research Core Facilities and HPC
Address: 53, Kabanbay Batyr ave.,
Astana, 010000, Republic of Kazakhstan

+7 (7172) 70 64 78
provost.cf@nu.edu.kz

Analysis Name	D:\Data\Zhanpeis_Issayev\PhGs\Sample_50_15_1_1083.d	Acquisition Date	3/17/2025 5:49:50 PM
Method	PhGs.m	Operator	Demo User
Sample Name	Sample_50	Instrument	impact II
Comment			

Acquisition Parameter

Source Type	ESI	Ion Polarity	Negative	Set Nebulizer	3.0 Bar
Focus	Active			Set Dry Heater	200 °C
Scan Begin	50 m/z	Set Capillary	3000 V	Set Dry Gas	7.0 l/min
Scan End	1300 m/z	Set End Plate Offset	-500 V	Set Divert Valve	Source

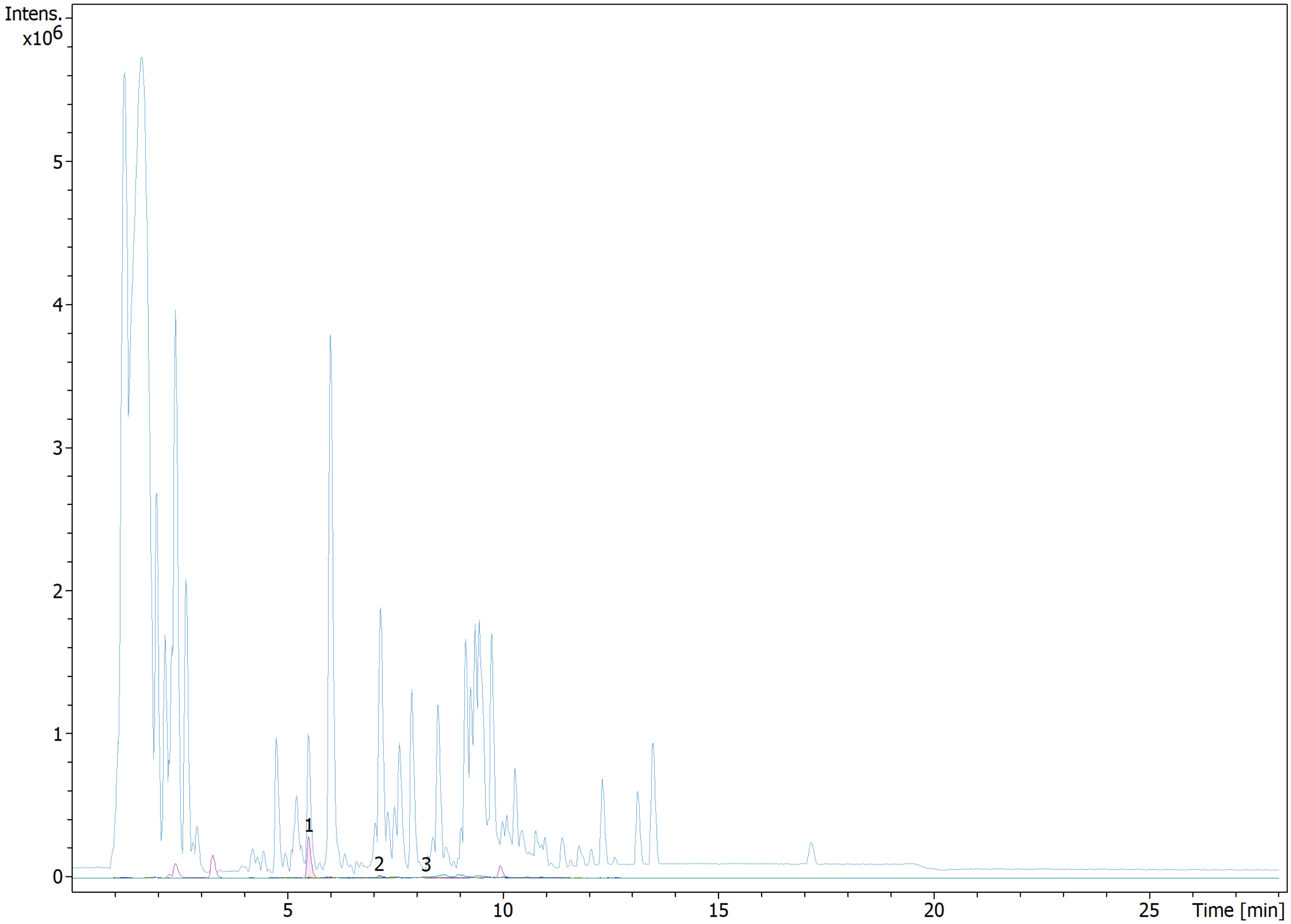

The results of the analyses conducted in our laboratory are intended solely for scientific research purposes. The laboratory is not responsible for the use of this data in legal matters.

AOE "Nazarbayev University"
Office of Research Core Facilities and HPC
Address: 53, Kabanbay Batyr ave.,
Astana, 010000, Republic of Kazakhstan

+7 (7172) 70 64 78
provost.cf@nu.edu.kz

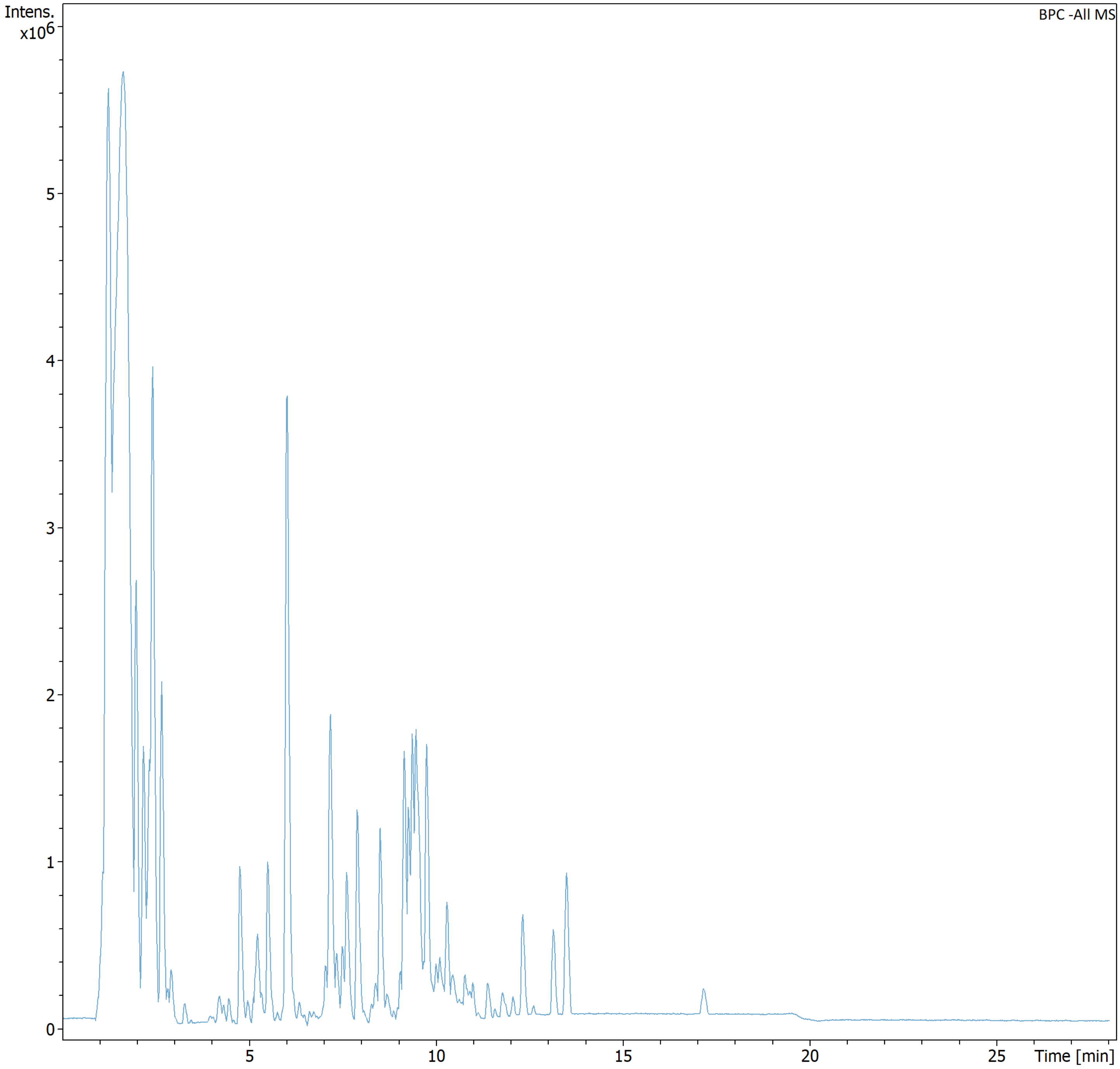

The results of the analyses conducted in our laboratory are intended solely for scientific research purposes. The laboratory is not responsible for the use of this data in legal matters.

NAZARBAYEV
UNIVERSITY

AOE "Nazarbayev University"
Office of Research Core Facilities and HPC
Address: 53, Kabanbay Batyr ave.,
Astana, 010000, Republic of Kazakhstan

+7 (7172) 70 64 78
provost.cf@nu.edu.kz

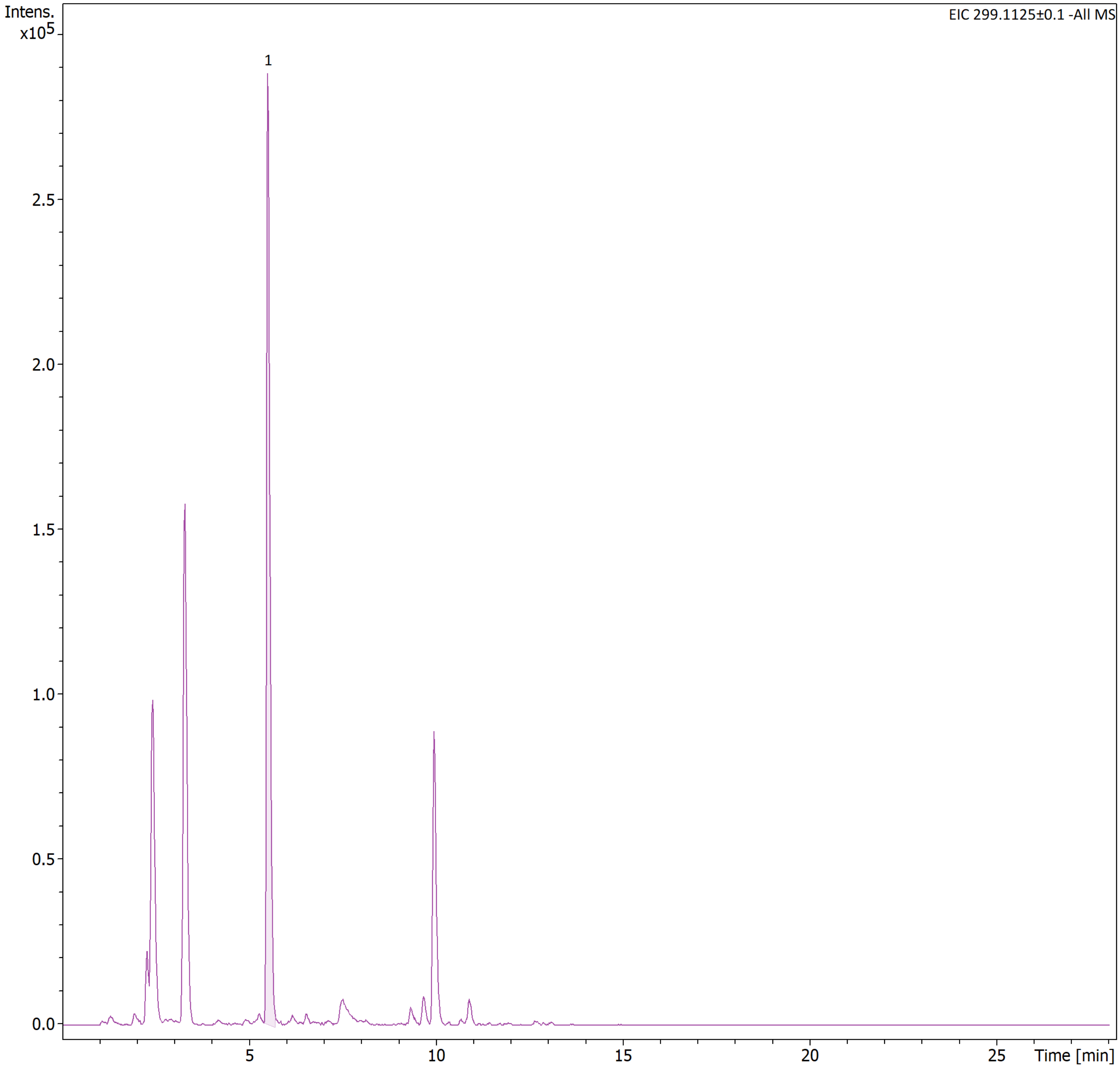

The results of the analyses conducted in our laboratory are intended solely for scientific research purposes. The laboratory is not responsible for the use of this data in legal matters.

AOE "Nazarbayev University"
Office of Research Core Facilities and HPC
Address: 53, Kabanbay Batyr ave.,
Astana, 010000, Republic of Kazakhstan

+7 (7172) 70 64 78
provost.cf@nu.edu.kz

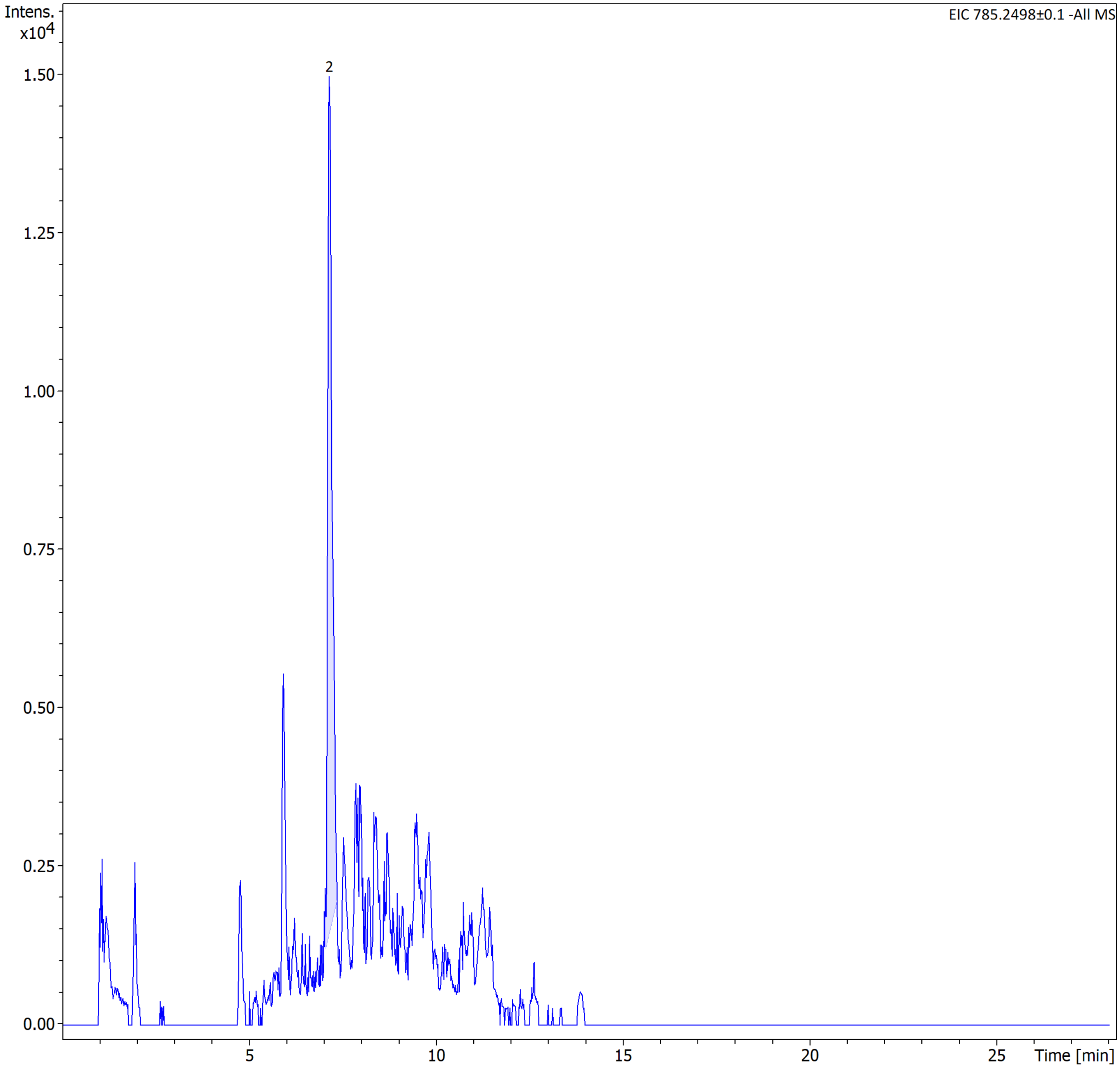

The results of the analyses conducted in our laboratory are intended solely for scientific research purposes. The laboratory is not responsible for the use of this data in legal matters.

AOE "Nazarbayev University"
Office of Research Core Facilities and HPC
Address: 53, Kabanbay Batyr ave.,
Astana, 010000, Republic of Kazakhstan

+7 (7172) 70 64 78
provost.cf@nu.edu.kz

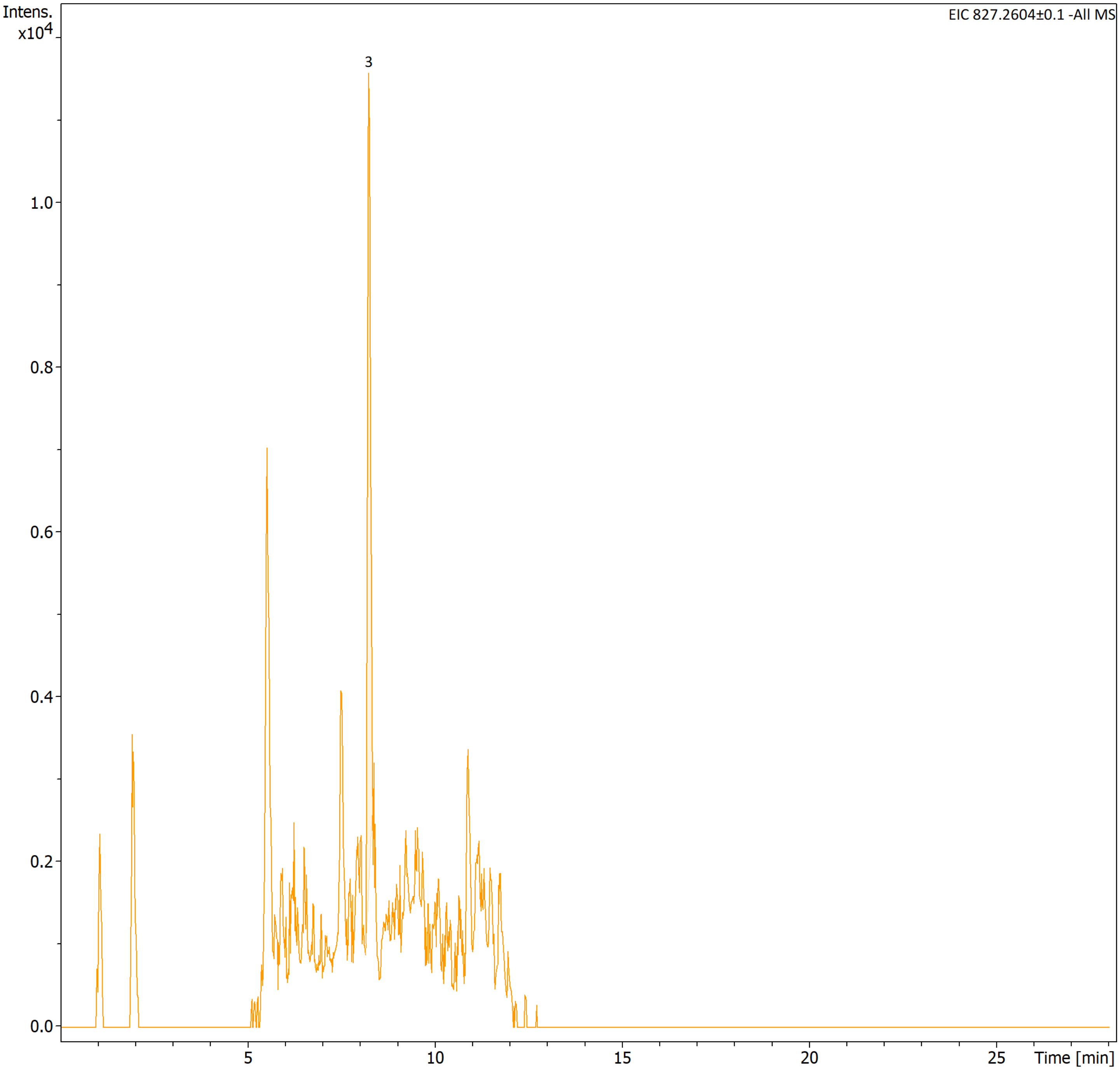

The results of the analyses conducted in our laboratory are intended solely for scientific research purposes. The laboratory is not responsible for the use of this data in legal matters.

AOE "Nazarbayev University"
Office of Research Core Facilities and HPC
Address: 53, Kabanbay Batyr ave.,
Astana, 010000, Republic of Kazakhstan

+7 (7172) 70 64 78
provost.cf@nu.edu.kz

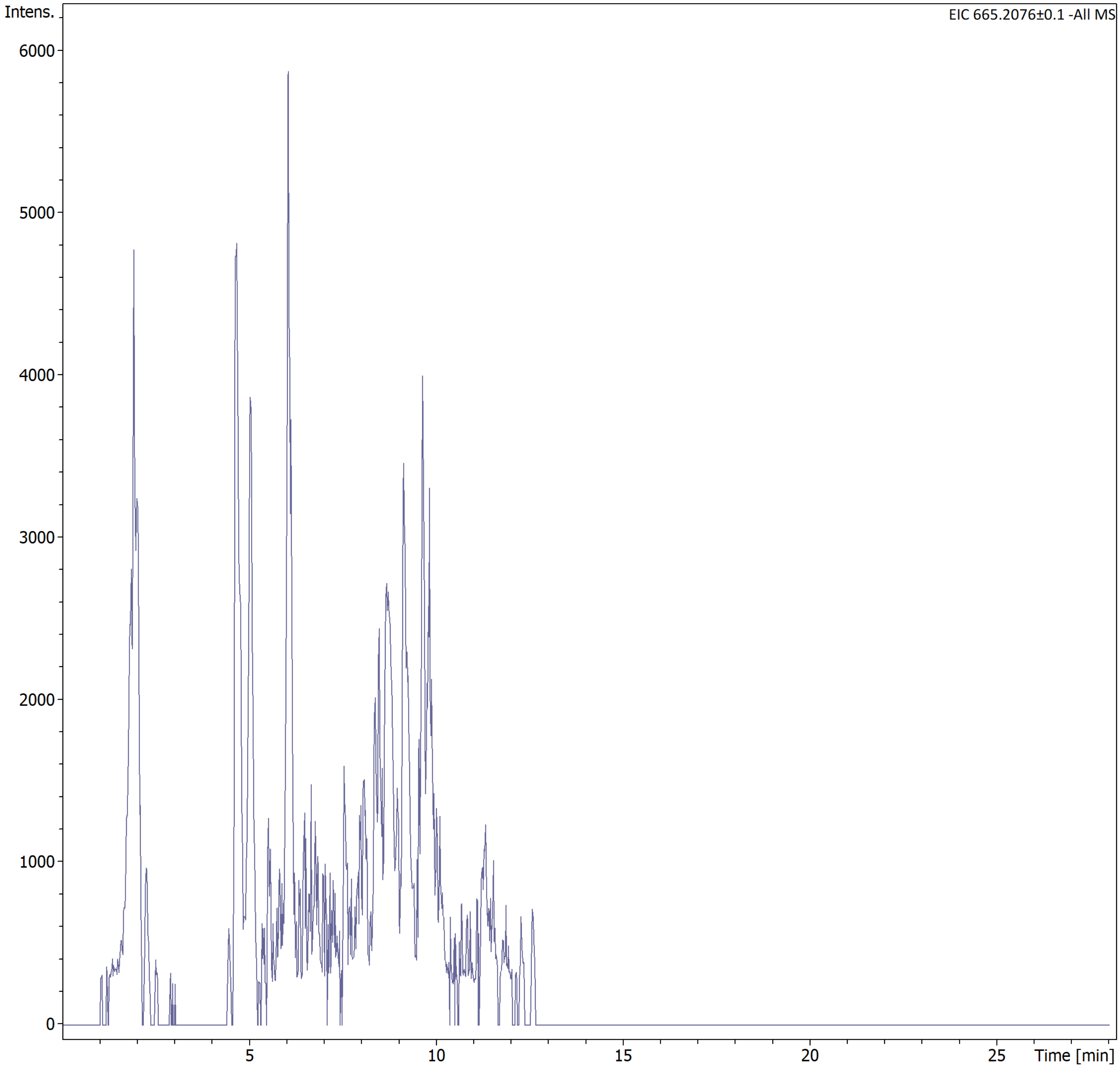

The results of the analyses conducted in our laboratory are intended solely for scientific research purposes. The laboratory is not responsible for the use of this data in legal matters.

AOE "Nazarbayev University"
Office of Research Core Facilities and HPC
Address: 53, Kabanbay Batyr ave.,
Astana, 010000, Republic of Kazakhstan

+7 (7172) 70 64 78
provost.cf@nu.edu.kz

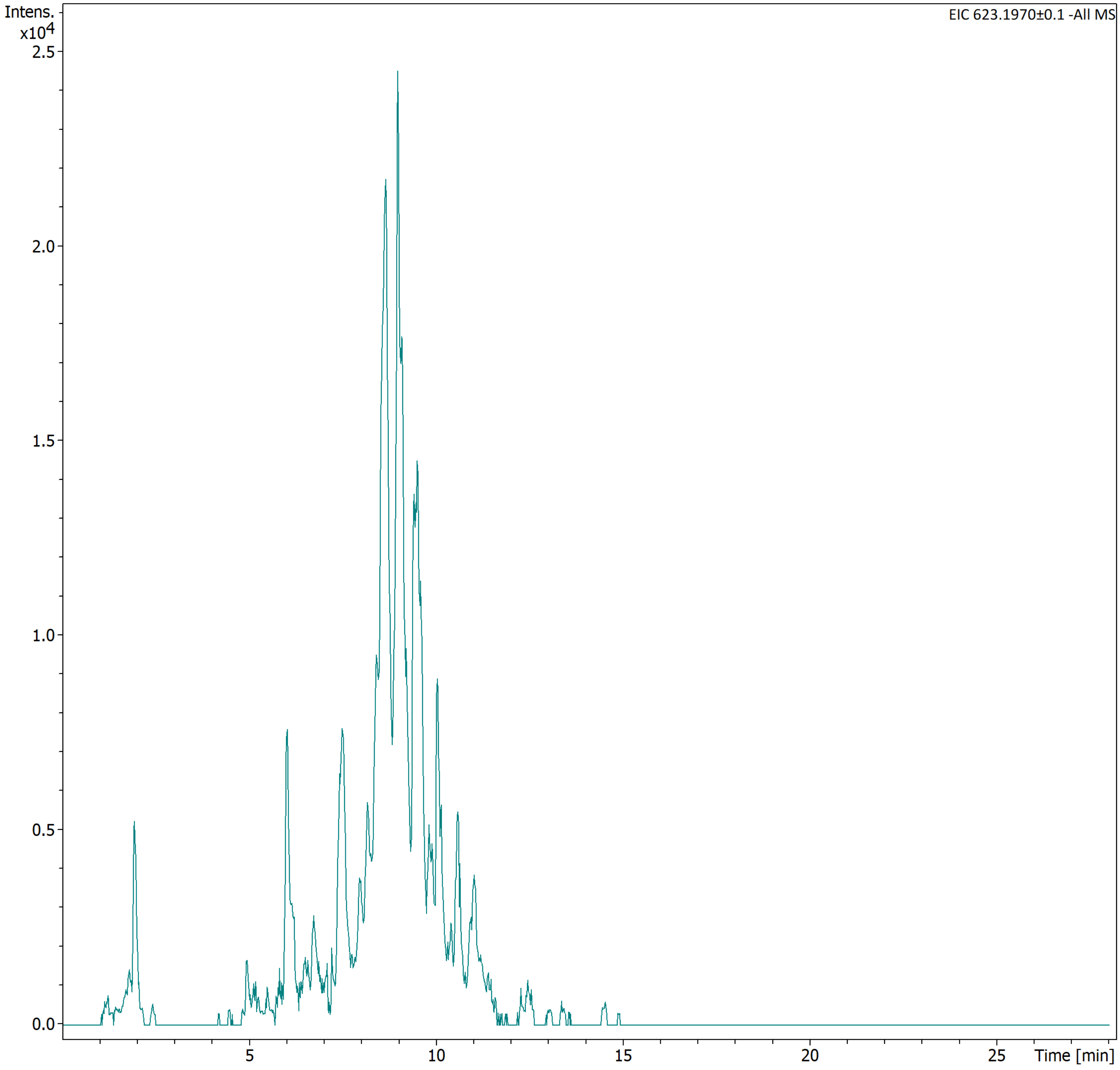

The results of the analyses conducted in our laboratory are intended solely for scientific research purposes. The laboratory is not responsible for the use of this data in legal matters.

AOE "Nazarbayev University"
Office of Research Core Facilities and HPC
Address: 53, Kabanbay Batyr ave.,
Astana, 010000, Republic of Kazakhstan

+7 (7172) 70 64 78
provost.cf@nu.edu.kz

#	RT [min]	Area	Int. Type	I	S/N	Trace	Max. m/z	FWHM [min]
1	5.5	1838166	Manual	289168	443.7	EIC 299.1125±0.1 -All MS	345.1186	0.1
2	7.1	111531	Manual	14969	26.8	EIC 785.2498±0.1 -All MS	121.0292	0.1
3	8.2	54244	Manual	11591	16.1	EIC 827.2604±0.1 -All MS	653.2069	0.1

Cmpd 1, 5.5 min

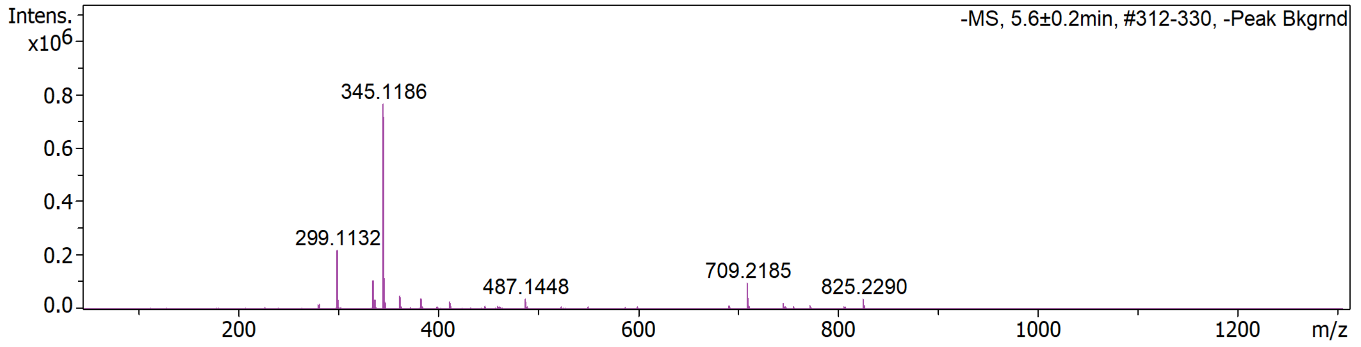

Cmpd 2, 7.1 min

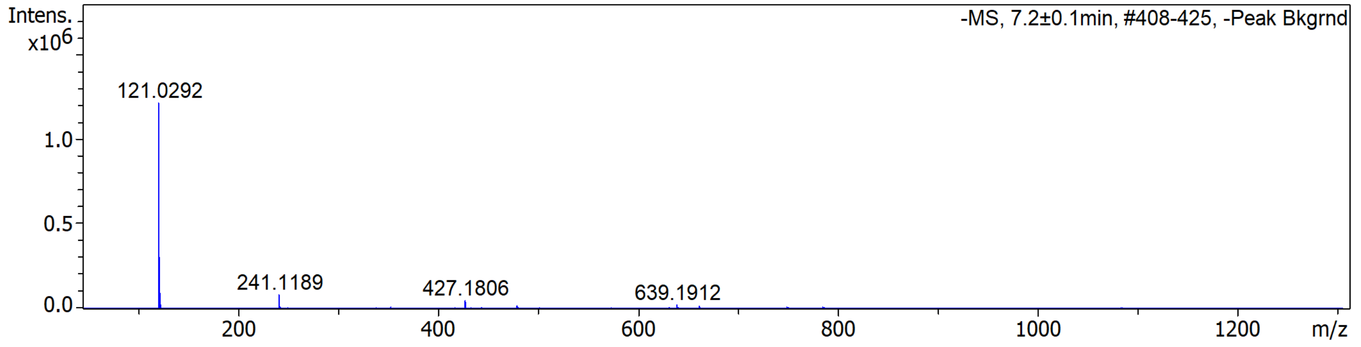

The results of the analyses conducted in our laboratory are intended solely for scientific research purposes. The laboratory is not responsible for the use of this data in legal matters.

AOE "Nazarbayev University"
Office of Research Core Facilities and HPC
Address: 53, Kabanbay Batyr ave.,
Astana, 010000, Republic of Kazakhstan

+7 (7172) 70 64 78
provost.cf@nu.edu.kz

Cmpd 3, 8.2 min

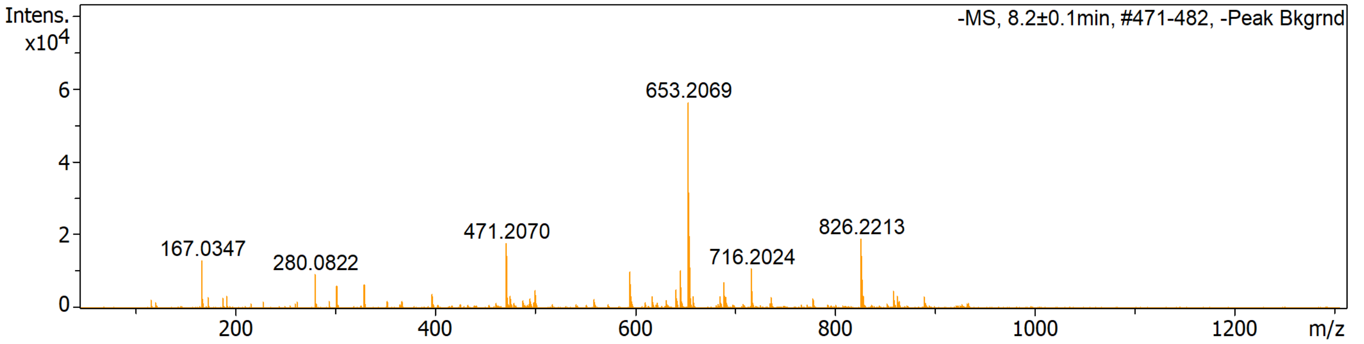

The results of the analyses conducted in our laboratory are intended solely for scientific research purposes. The laboratory is not responsible for the use of this data in legal matters.
