# Supplementary material for: Stress-Induced Secondary Metabolite Profiling in Cistanche deserticola Callus Cultures: Insights from GC-MS and HPLC-MS Analysis
Source: Int J Mol Sci. 2025 Jun 25;26(13):6091. doi: 10.3390/ijms26136091 (PMC12250269; doi:10.3390/ijms26136091)
Supplement: Supplementary file 1 [file ijms-26-06091-s001.zip › Supplementary materials S9_qTOF_UHPLC-MS_Data/qTOF_UHPLC-MS_Results/Sample_6.pdf]

AOE "Nazarbayev University"
Office of Research Core Facilities and HPC
Address: 53, Kabanbay Batyr ave.,
Astana, 010000, Republic of Kazakhstan

+7 (7172) 70 64 78
provost.cf@nu.edu.kz

Analysis Name	D:\Data\Zhanpeis_Issayev\PhGs\Sample_6_4_1_1071.d	Acquisition Date	3/17/2025 12:00:08 PM
Method	PhGs.m	Operator	Demo User
Sample Name	Sample_6	Instrument	impact II
Comment			

Acquisition Parameter

Source Type	ESI	Ion Polarity	Negative	Set Nebulizer	3.0 Bar
Focus	Active			Set Dry Heater	200 °C
Scan Begin	50 m/z	Set Capillary	3000 V	Set Dry Gas	7.0 l/min
Scan End	1300 m/z	Set End Plate Offset	-500 V	Set Divert Valve	Source

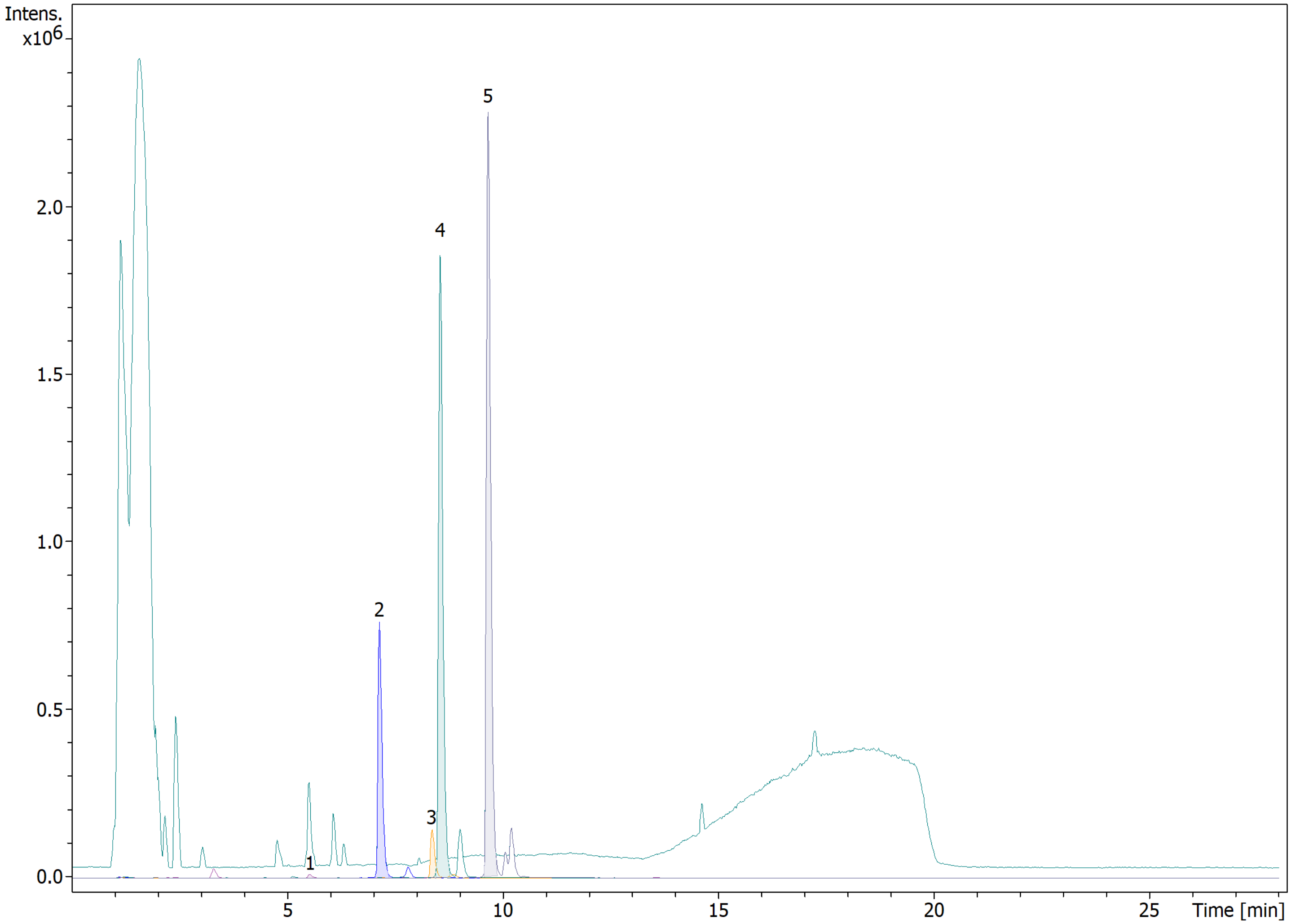

The results of the analyses conducted in our laboratory are intended solely for scientific research purposes. The laboratory is not responsible for the use of this data in legal matters.

AOE "Nazarbayev University"
Office of Research Core Facilities and HPC
Address: 53, Kabanbay Batyr ave.,
Astana, 010000, Republic of Kazakhstan

+7 (7172) 70 64 78
provost.cf@nu.edu.kz

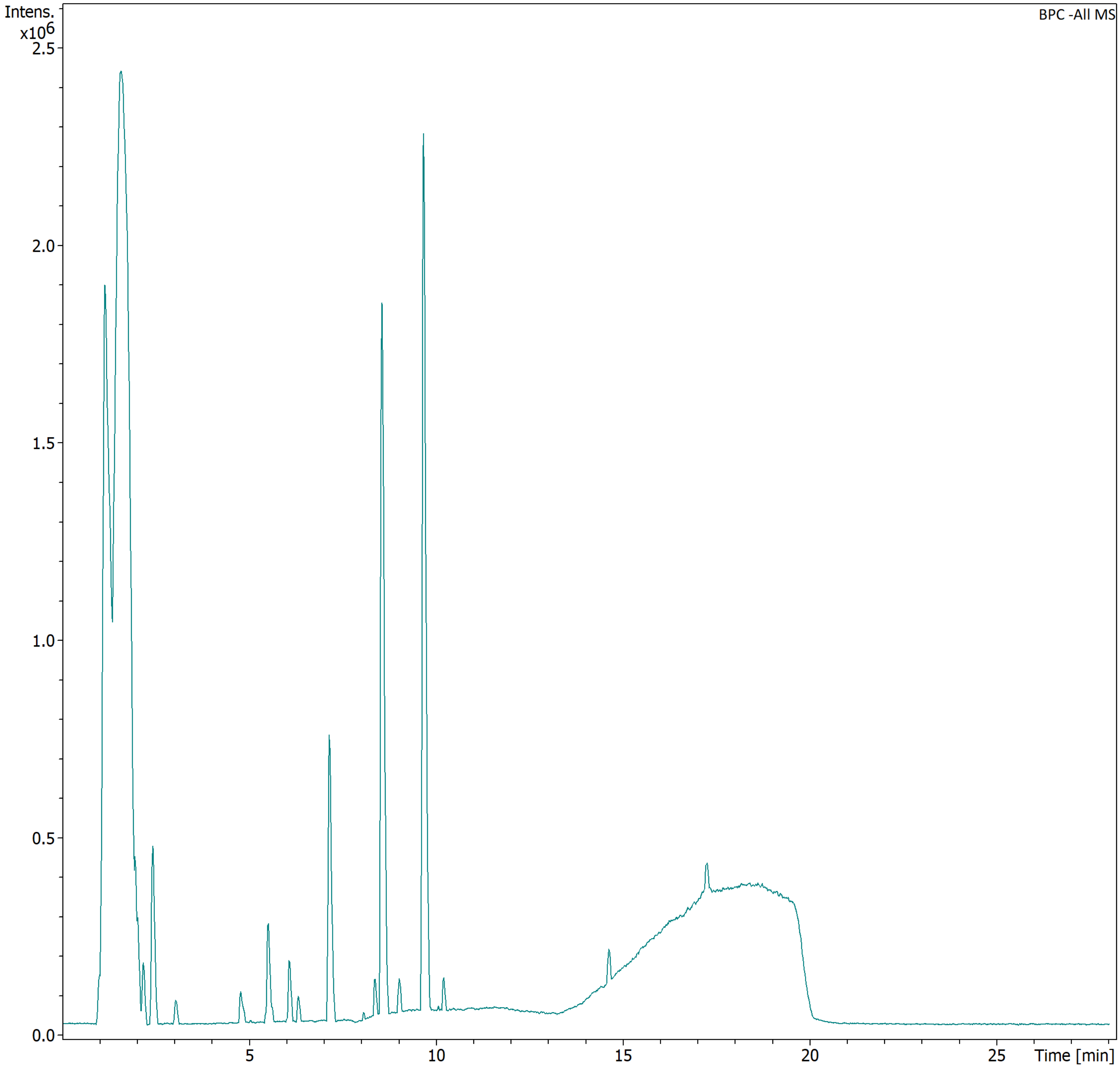

The results of the analyses conducted in our laboratory are intended solely for scientific research purposes. The laboratory is not responsible for the use of this data in legal matters.

AOE "Nazarbayev University"
Office of Research Core Facilities and HPC
Address: 53, Kabanbay Batyr ave.,
Astana, 010000, Republic of Kazakhstan

+7 (7172) 70 64 78
provost.cf@nu.edu.kz

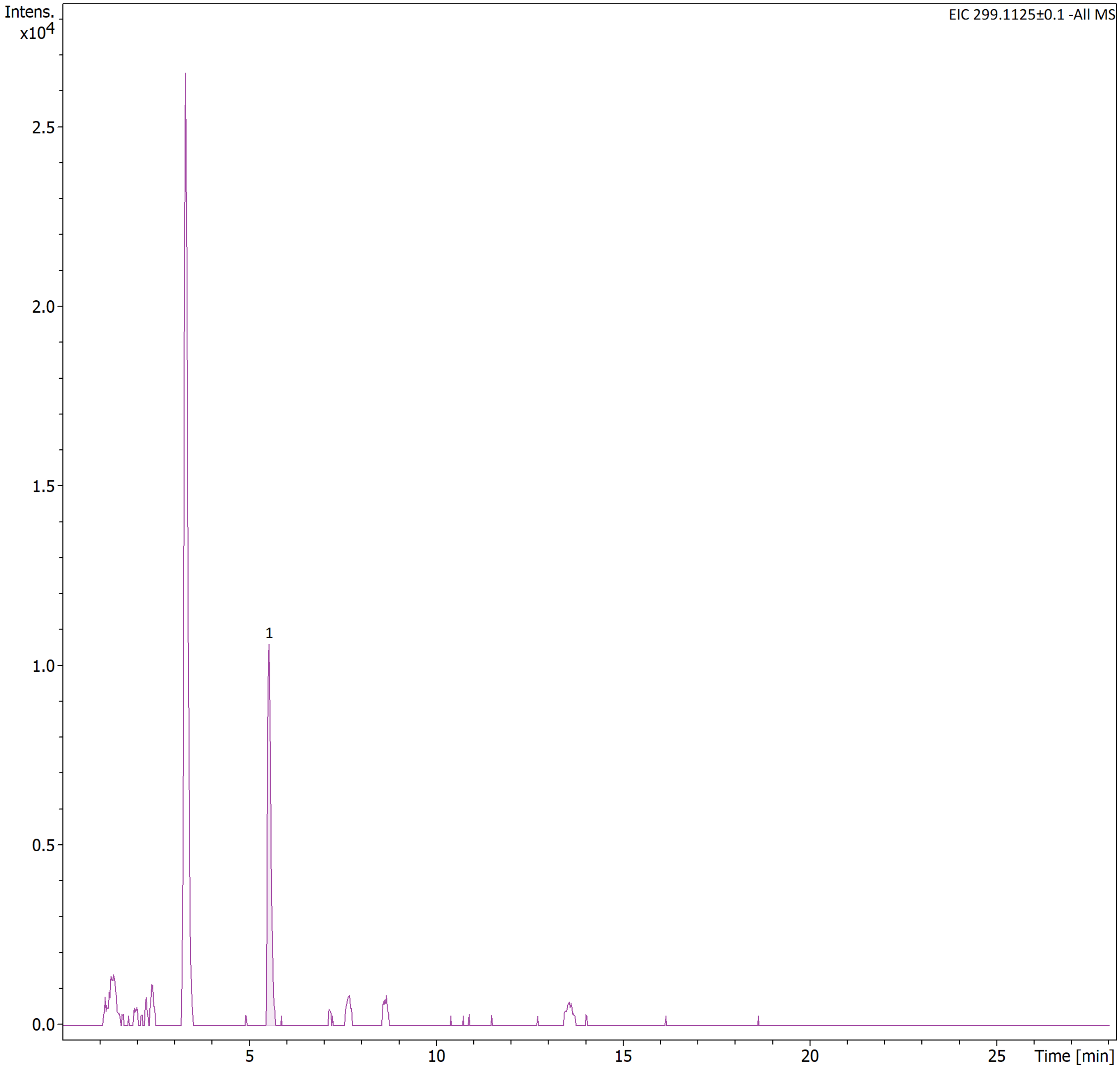

The results of the analyses conducted in our laboratory are intended solely for scientific research purposes. The laboratory is not responsible for the use of this data in legal matters.

AOE "Nazarbayev University"
Office of Research Core Facilities and HPC
Address: 53, Kabanbay Batyr ave.,
Astana, 010000, Republic of Kazakhstan

+7 (7172) 70 64 78
provost.cf@nu.edu.kz

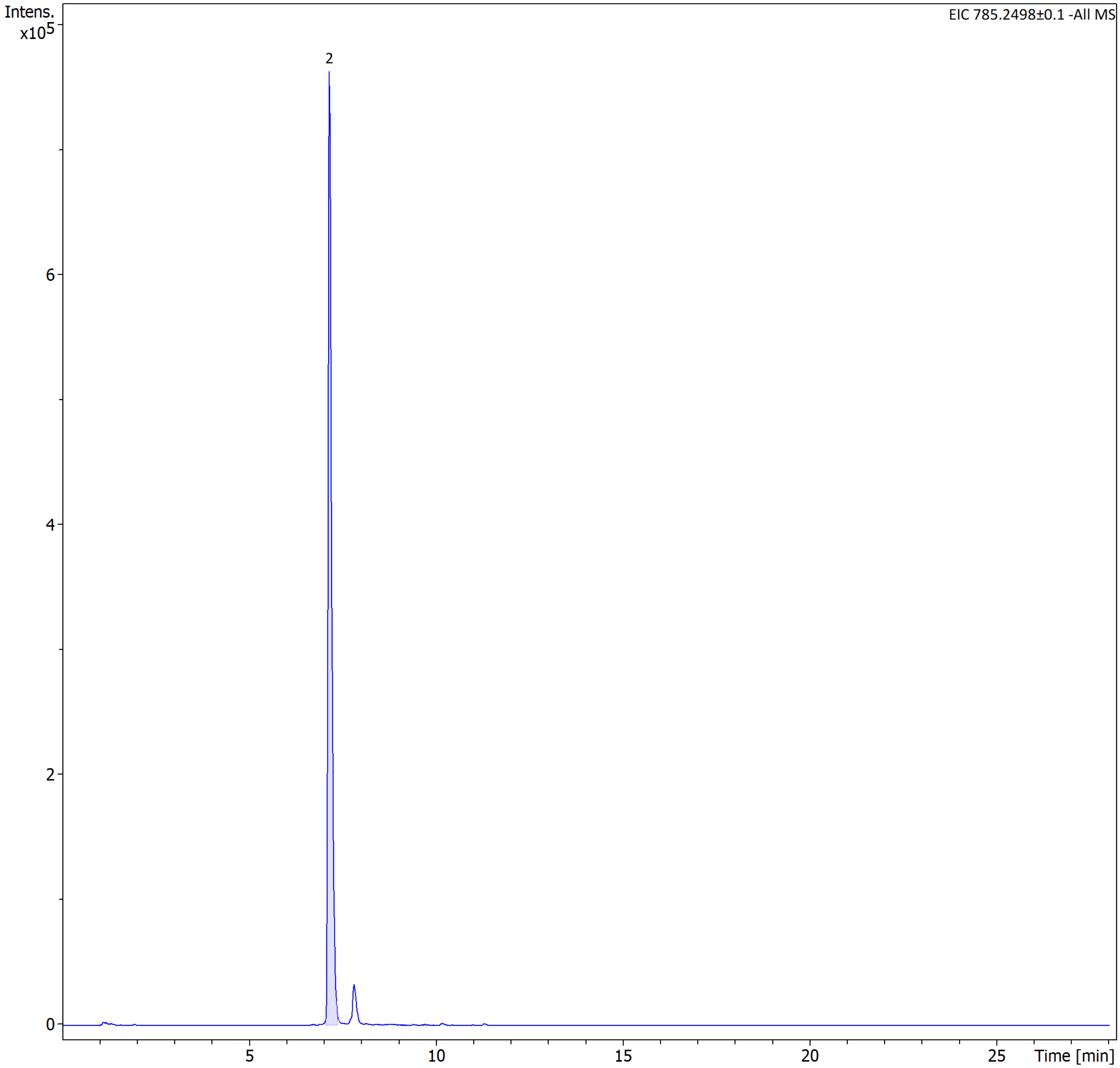

The results of the analyses conducted in our laboratory are intended solely for scientific research purposes. The laboratory is not responsible for the use of this data in legal matters.

AOE "Nazarbayev University"
Office of Research Core Facilities and HPC
Address: 53, Kabanbay Batyr ave.,
Astana, 010000, Republic of Kazakhstan

+7 (7172) 70 64 78
provost.cf@nu.edu.kz

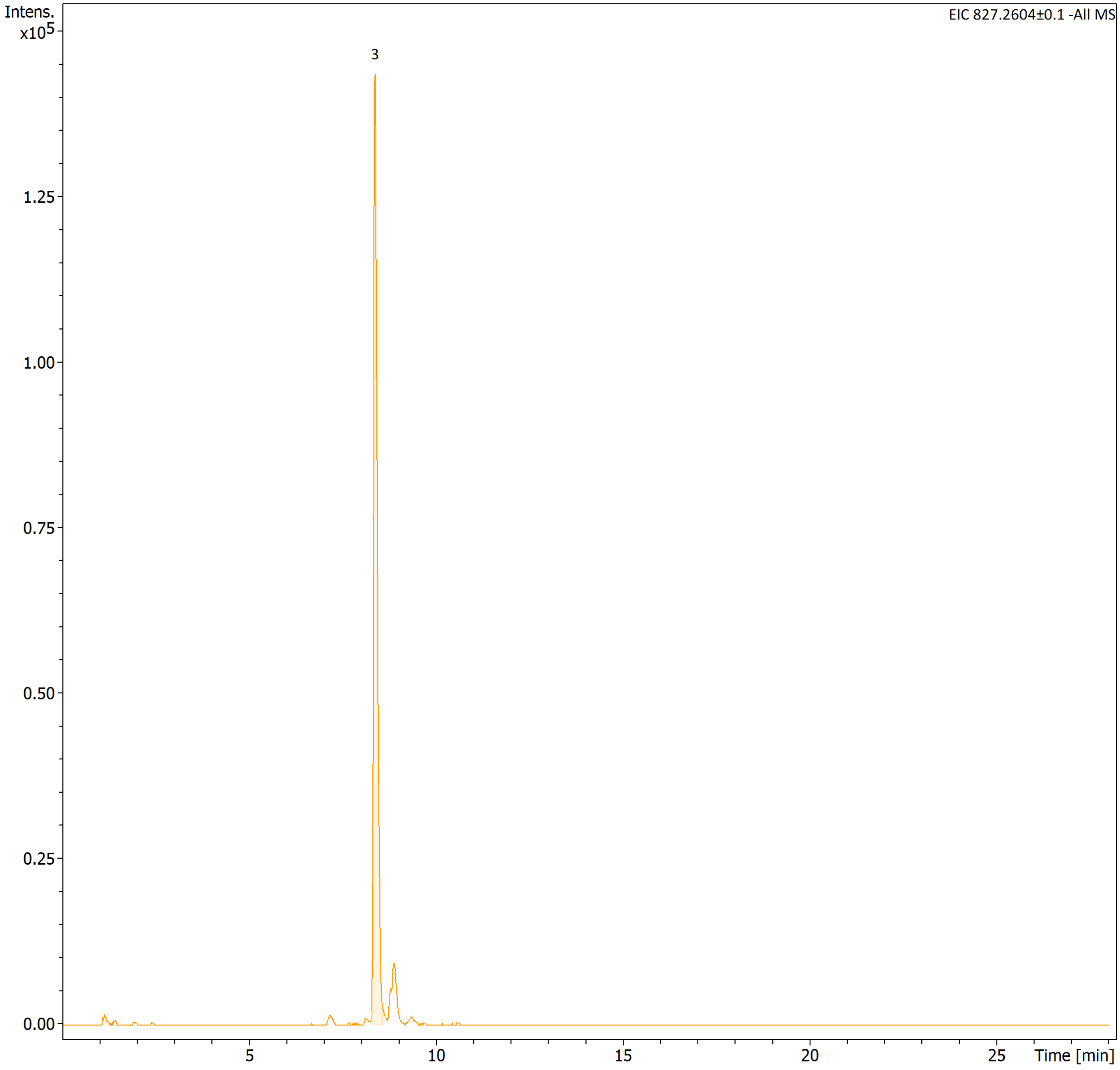

The results of the analyses conducted in our laboratory are intended solely for scientific research purposes. The laboratory is not responsible for the use of this data in legal matters.

AOE "Nazarbayev University"
Office of Research Core Facilities and HPC
Address: 53, Kabanbay Batyr ave.,
Astana, 010000, Republic of Kazakhstan

+7 (7172) 70 64 78
provost.cf@nu.edu.kz

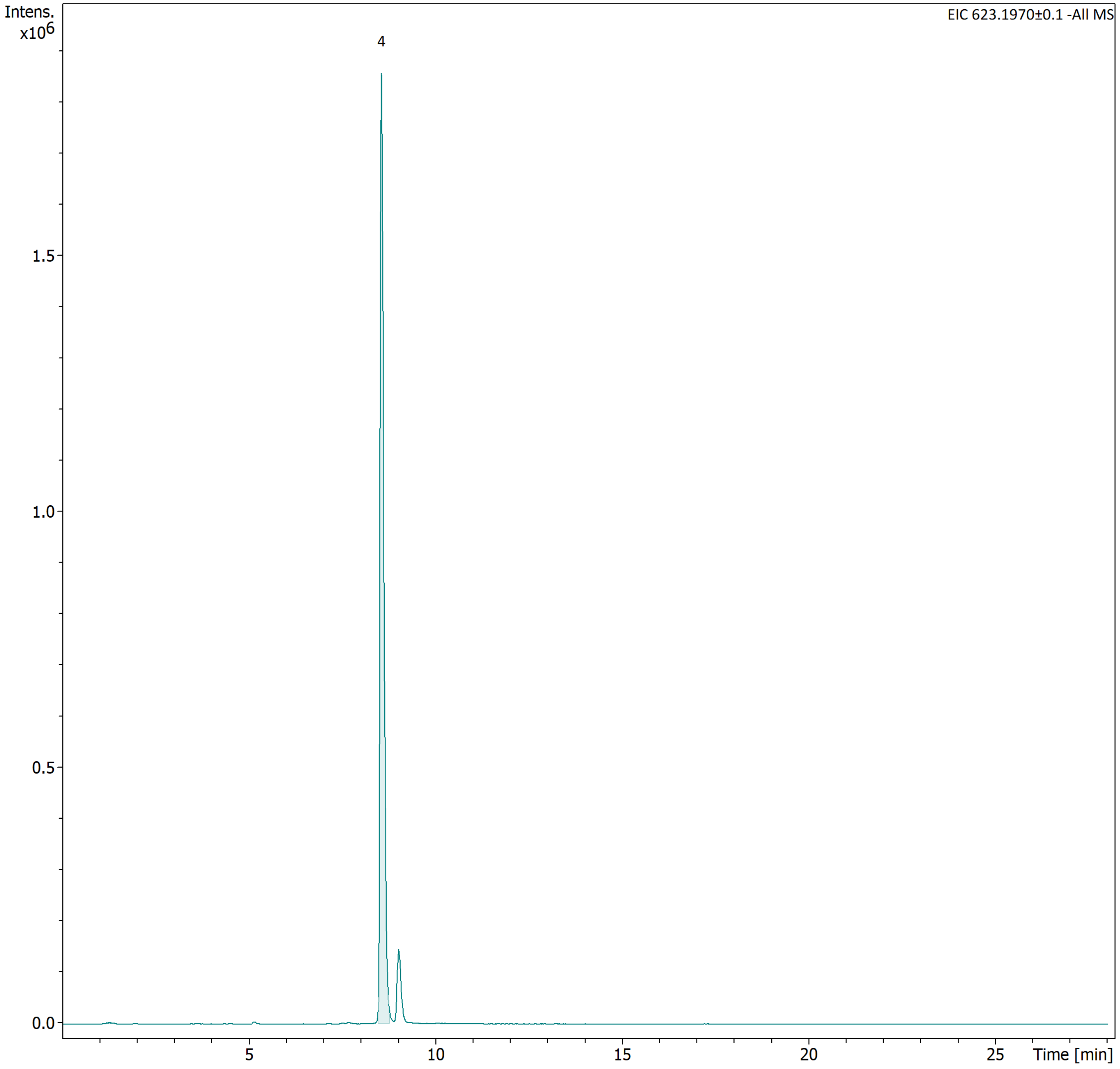

The results of the analyses conducted in our laboratory are intended solely for scientific research purposes. The laboratory is not responsible for the use of this data in legal matters.

NAZARBAYEV UNIVERSITY

AOE "Nazarbayev University"
Office of Research Core Facilities and HPC
Address: 53, Kabanbay Batyr ave.,
Astana, 010000, Republic of Kazakhstan

+7 (7172) 70 64 78
provost.cf@nu.edu.kz

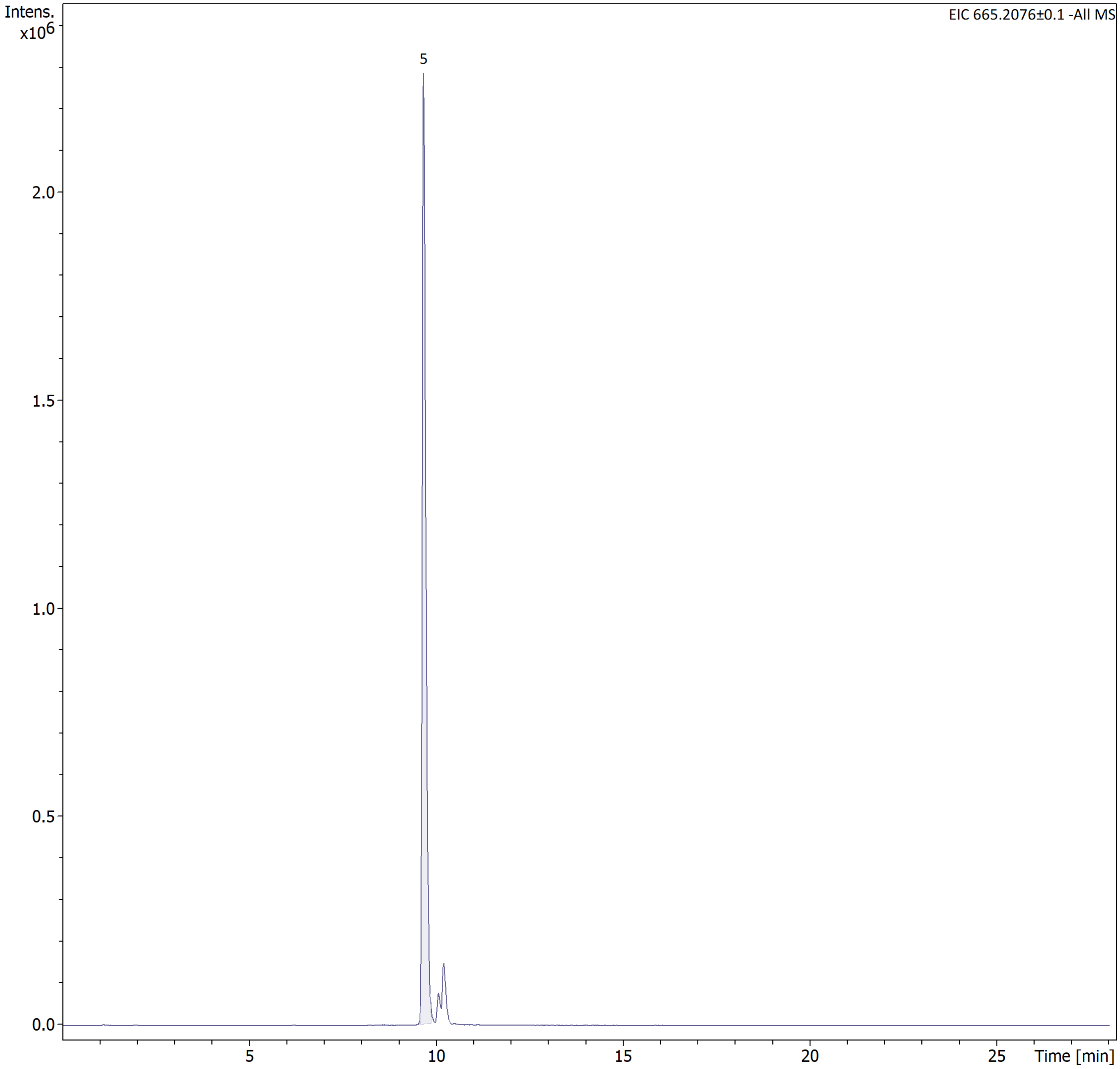

The results of the analyses conducted in our laboratory are intended solely for scientific research purposes. The laboratory is not responsible for the use of this data in legal matters.

AOE "Nazarbayev University"
Office of Research Core Facilities and HPC
Address: 53, Kabanbay Batyr ave.,
Astana, 010000, Republic of Kazakhstan

+7 (7172) 70 64 78
provost.cf@nu.edu.kz

#	RT [min]	Area	Int. Type	I	S/N	Trace	Max. m/z	FWHM [min]
1	5.5	67238	Chromatogram	10645	27.2	EIC 299.1125±0.1 -All MS	391.1250	0.1
2	7.2	5075664	Chromatogram	765345	1791.0	EIC 785.2498±0.1 -All MS	785.2515	0.1
3	8.4	967057	Chromatogram	145033	407.6	EIC 827.2604±0.1 -All MS	827.2624	0.1
4	8.6	12875643	Chromatogram	1899007	6857.2	EIC 623.1970±0.1 -All MS	623.1985	0.1
5	9.7	15789001	Chromatogram	2296803	8654.4	EIC 665.2076±0.1 -All MS	665.2095	0.1

Cmpd 1, 5.5 min

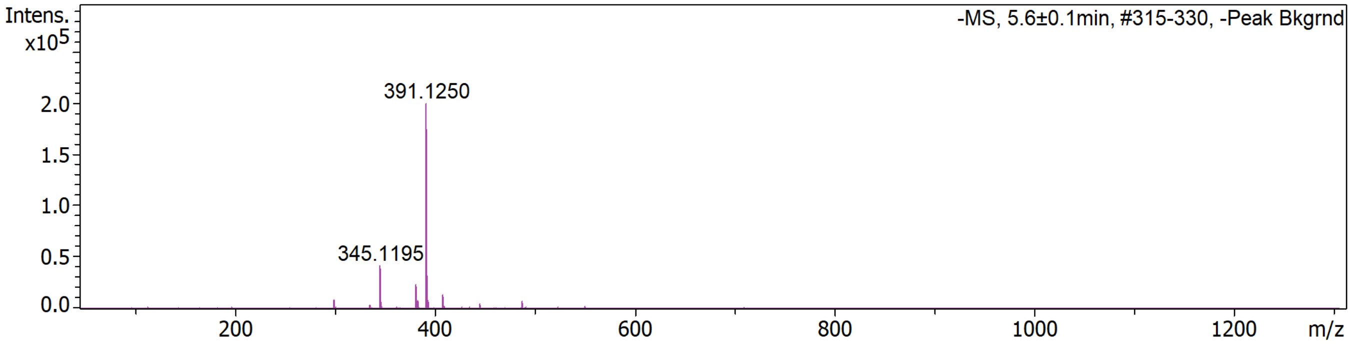

Cmpd 2, 7.2 min

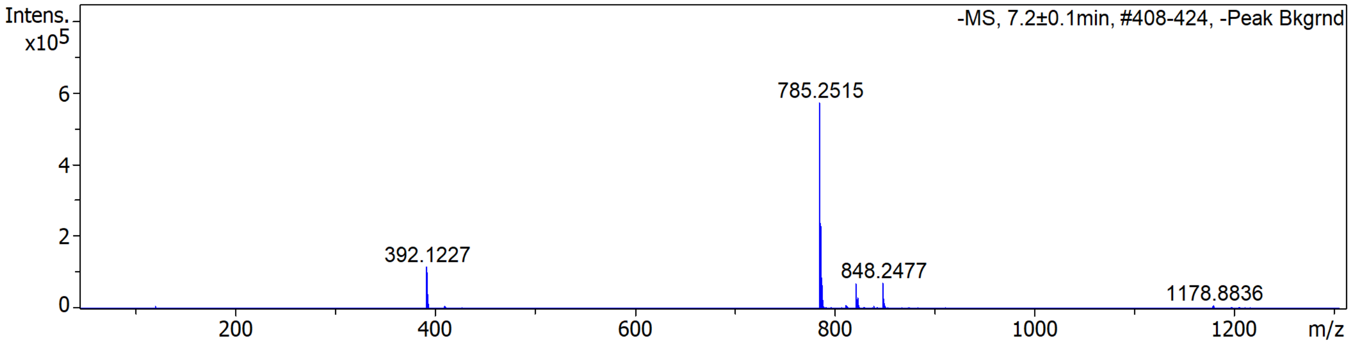

The results of the analyses conducted in our laboratory are intended solely for scientific research purposes. The laboratory is not responsible for the use of this data in legal matters.

AOE "Nazarbayev University"
Office of Research Core Facilities and HPC
Address: 53, Kabanbay Batyr ave.,
Astana, 010000, Republic of Kazakhstan

+7 (7172) 70 64 78
provost.cf@nu.edu.kz

Cmpd 3, 8.4 min

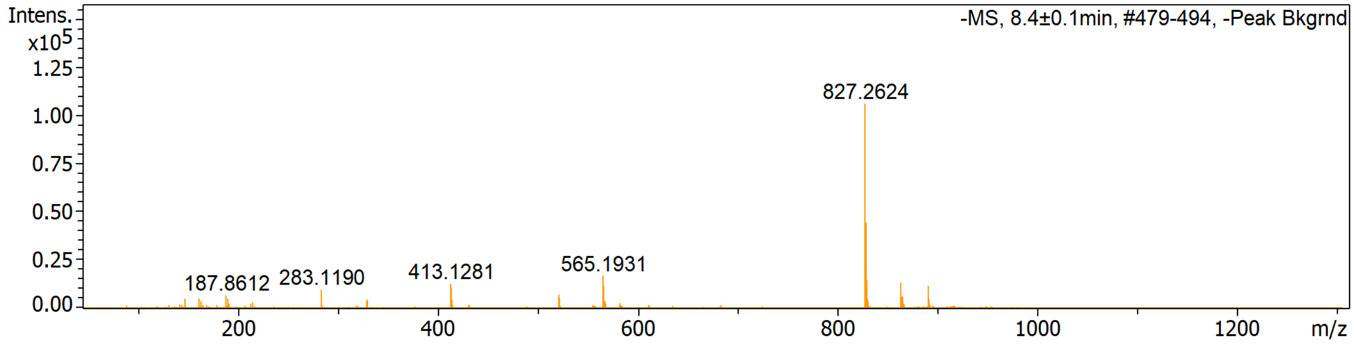

Cmpd 4, 8.6 min

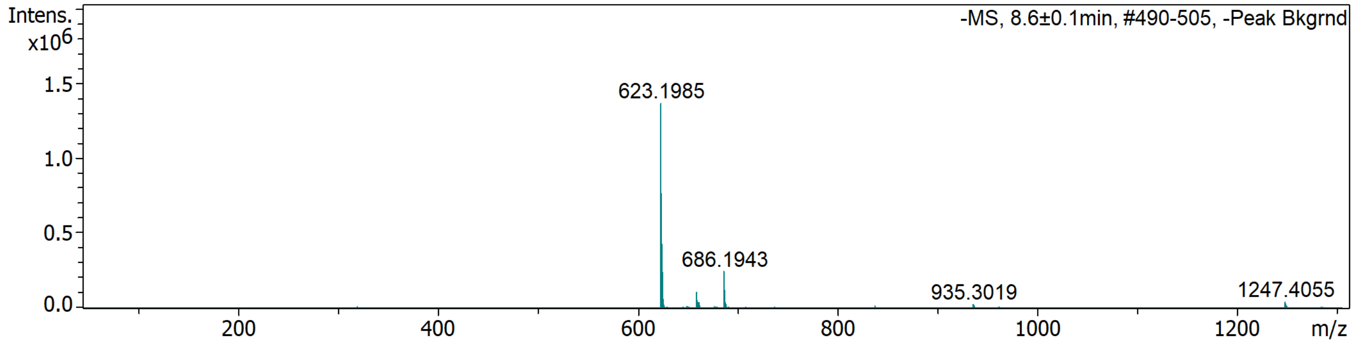

Cmpd 5, 9.7 min

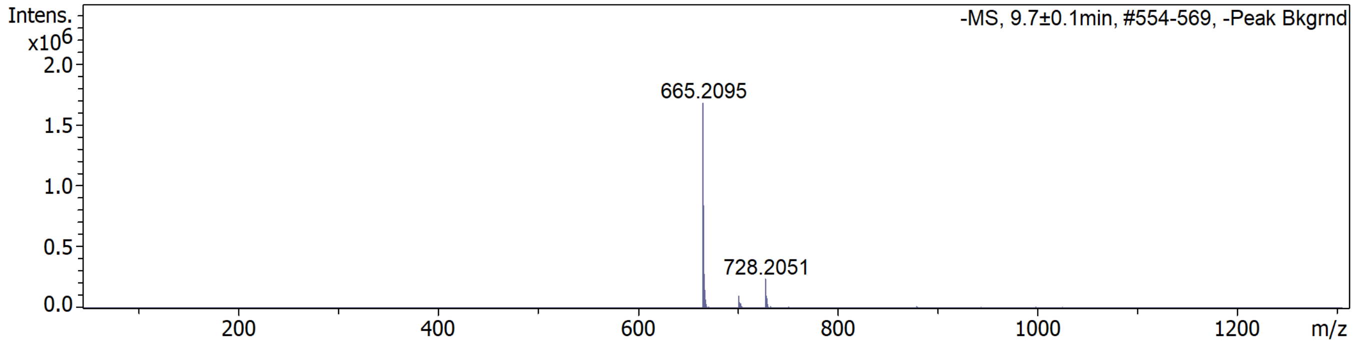

The results of the analyses conducted in our laboratory are intended solely for scientific research purposes. The laboratory is not responsible for the use of this data in legal matters.
