# Supplementary material for: Stress-Induced Secondary Metabolite Profiling in Cistanche deserticola Callus Cultures: Insights from GC-MS and HPLC-MS Analysis
Source: Int J Mol Sci. 2025 Jun 25;26(13):6091. doi: 10.3390/ijms26136091 (PMC12250269; doi:10.3390/ijms26136091)
Supplement: Supplementary file 1 [file ijms-26-06091-s001.zip › Supplementary materials S9_qTOF_UHPLC-MS_Data/qTOF_UHPLC-MS_Results/Standart Samples.pdf]

AOE "Nazarbayev University"
Office of Research Core Facilities and HPC
Address: 53, Kabanbay Batyr ave.,
Astana, 010000, Republic of Kazakhstan

+7 (7172) 70 64 78
provost.cf@nu.edu.kz

Analysis Name	D:\Data\Zhanpeis_Issayev\PhGs\St_mix_10ulst_50ulmeoh_2_1_1069.d	Acquisition Date	3/15/2025 5:45:38 PM
Method	PhGs.m	Operator	Demo User
Sample Name	St_mix_10ulst_50ulmeoh	Instrument	impact II
Comment			

Acquisition Parameter

Source Type	ESI	Ion Polarity	Negative	Set Nebulizer	3.0 Bar
Focus	Active			Set Dry Heater	200 °C
Scan Begin	50 m/z	Set Capillary	3000 V	Set Dry Gas	7.0 l/min
Scan End	1300 m/z	Set End Plate Offset	-500 V	Set Divert Valve	Source

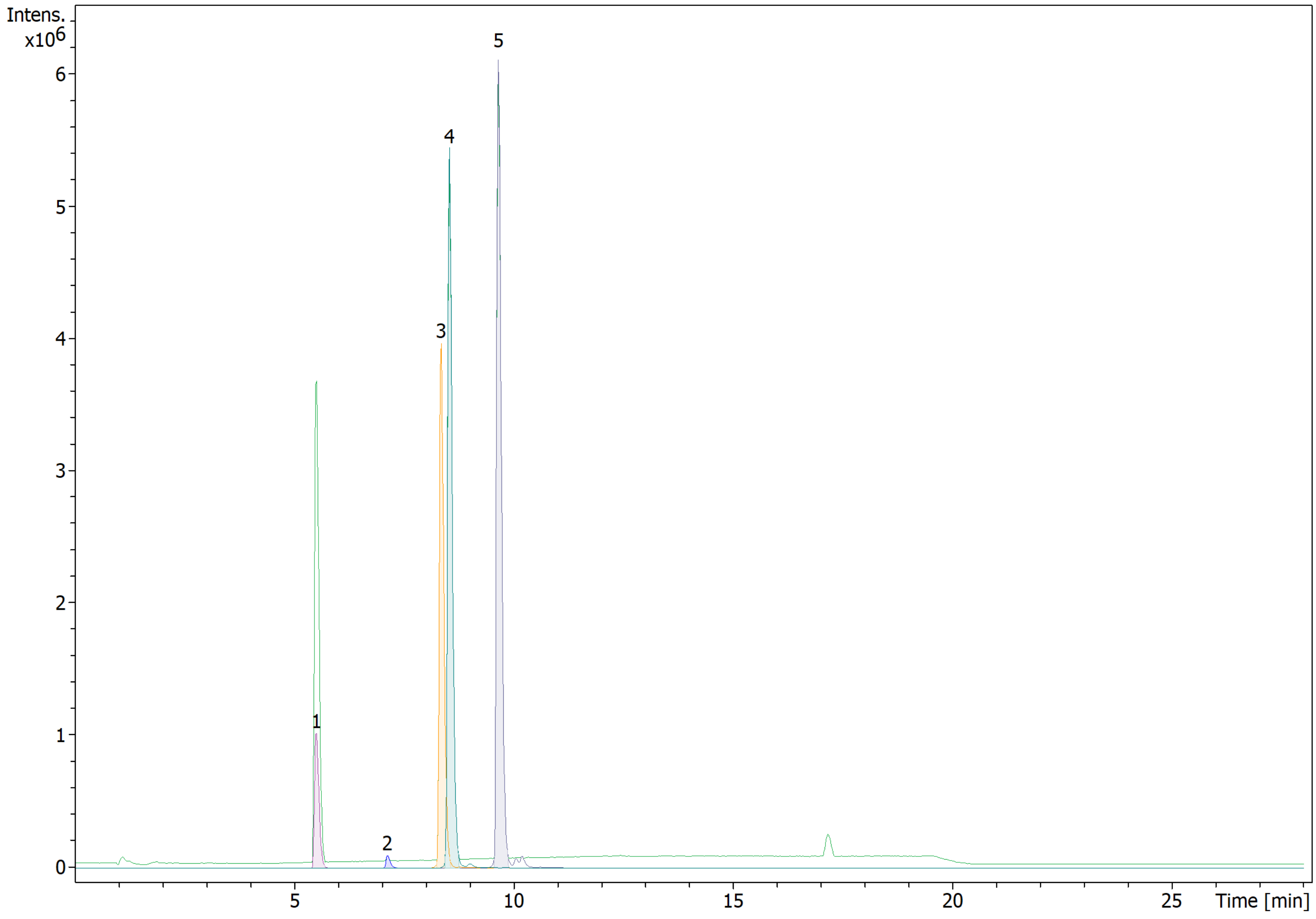

The results of the analyses conducted in our laboratory are intended solely for scientific research purposes. The laboratory is not responsible for the use of this data in legal matters.

NAZARBAYEV UNIVERSITY

AOE "Nazarbayev University"
Office of Research Core Facilities and HPC
Address: 53, Kabanbay Batyr ave.,
Astana, 010000, Republic of Kazakhstan

+7 (7172) 70 64 78
provost.cf@nu.edu.kz

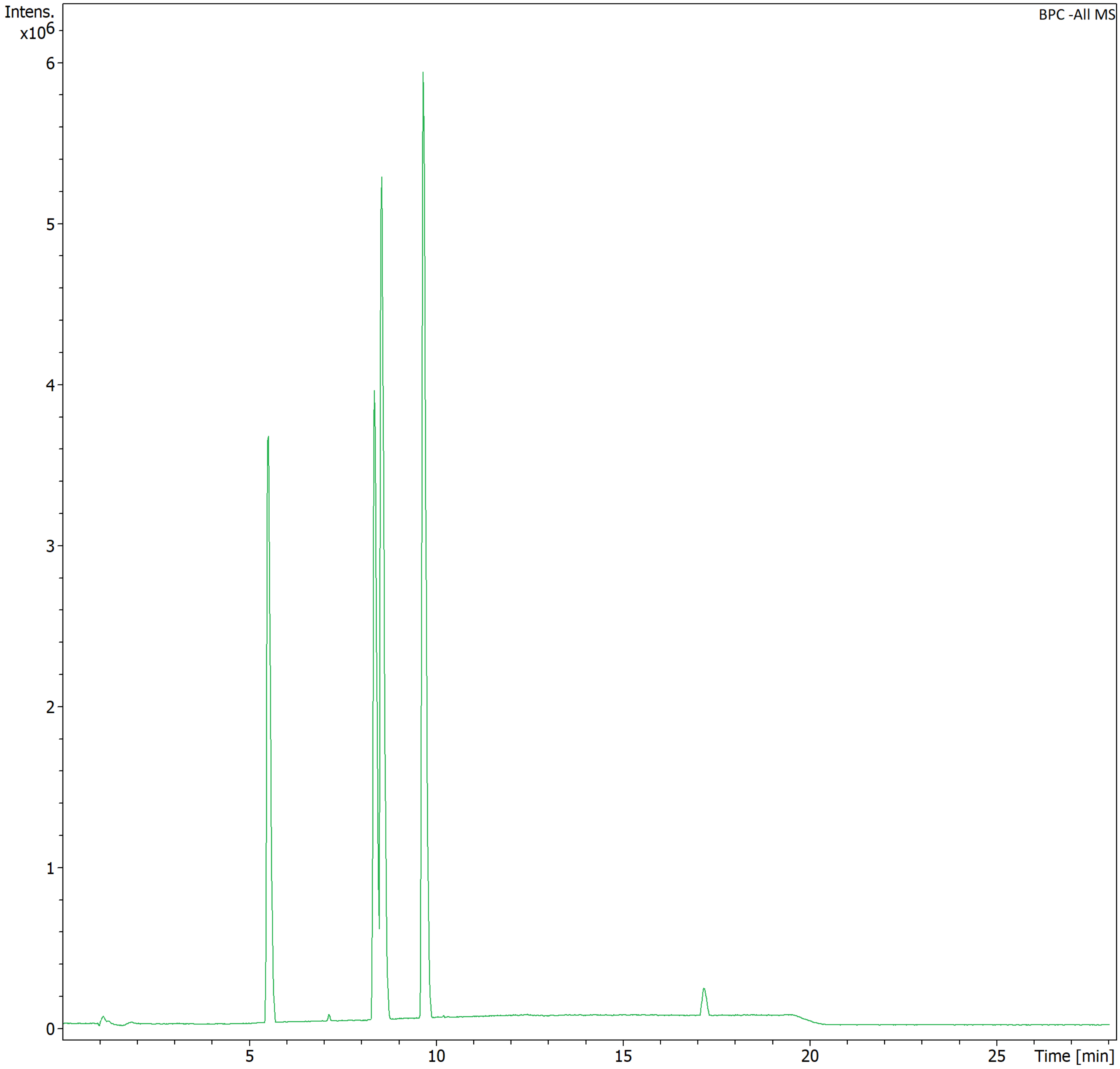

The results of the analyses conducted in our laboratory are intended solely for scientific research purposes. The laboratory is not responsible for the use of this data in legal matters.

AOE "Nazarbayev University"
Office of Research Core Facilities and HPC
Address: 53, Kabanbay Batyr ave.,
Astana, 010000, Republic of Kazakhstan

+7 (7172) 70 64 78
provost.cf@nu.edu.kz

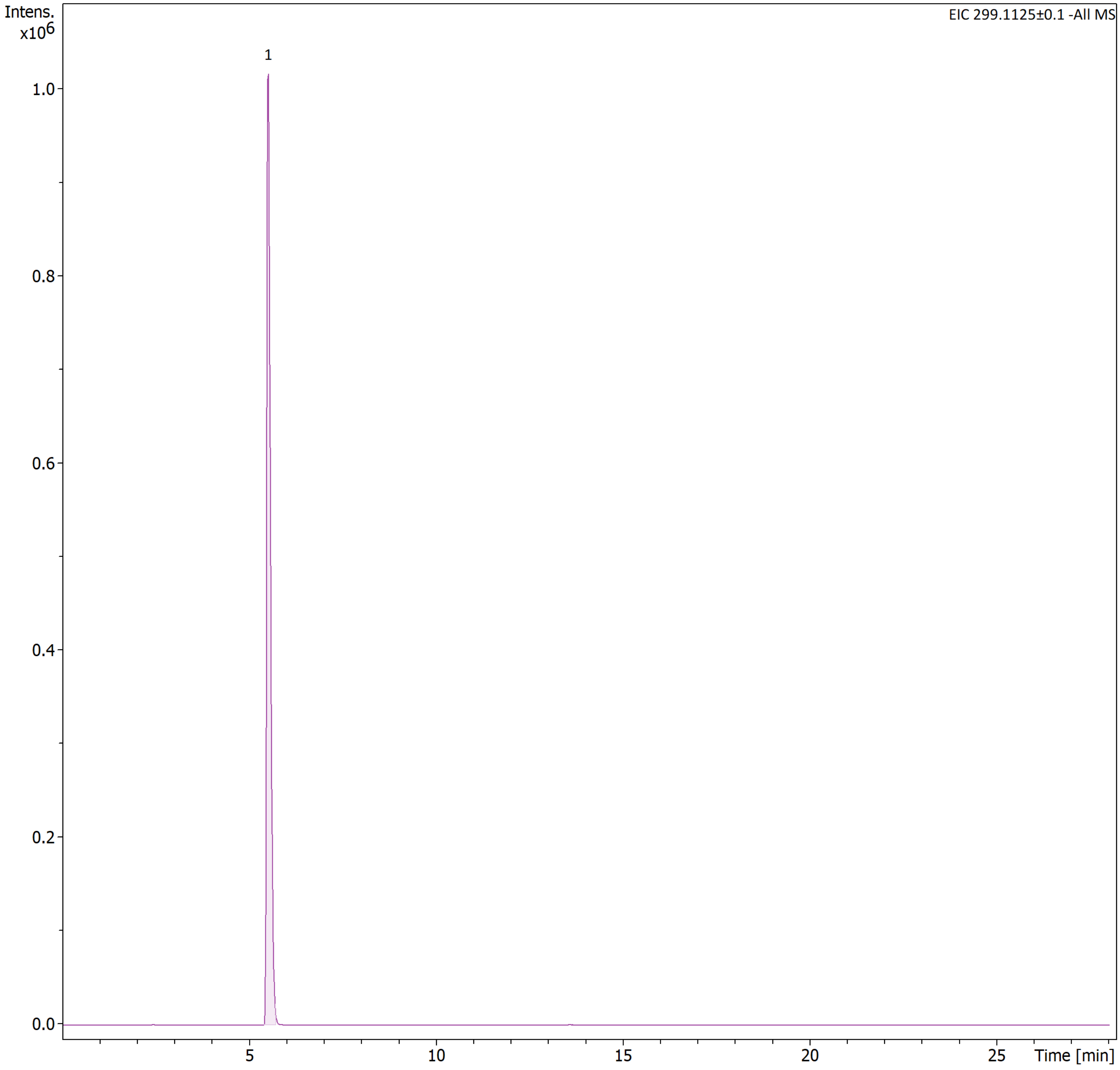

The results of the analyses conducted in our laboratory are intended solely for scientific research purposes. The laboratory is not responsible for the use of this data in legal matters.

AOE "Nazarbayev University"
Office of Research Core Facilities and HPC
Address: 53, Kabanbay Batyr ave.,
Astana, 010000, Republic of Kazakhstan

+7 (7172) 70 64 78
provost.cf@nu.edu.kz

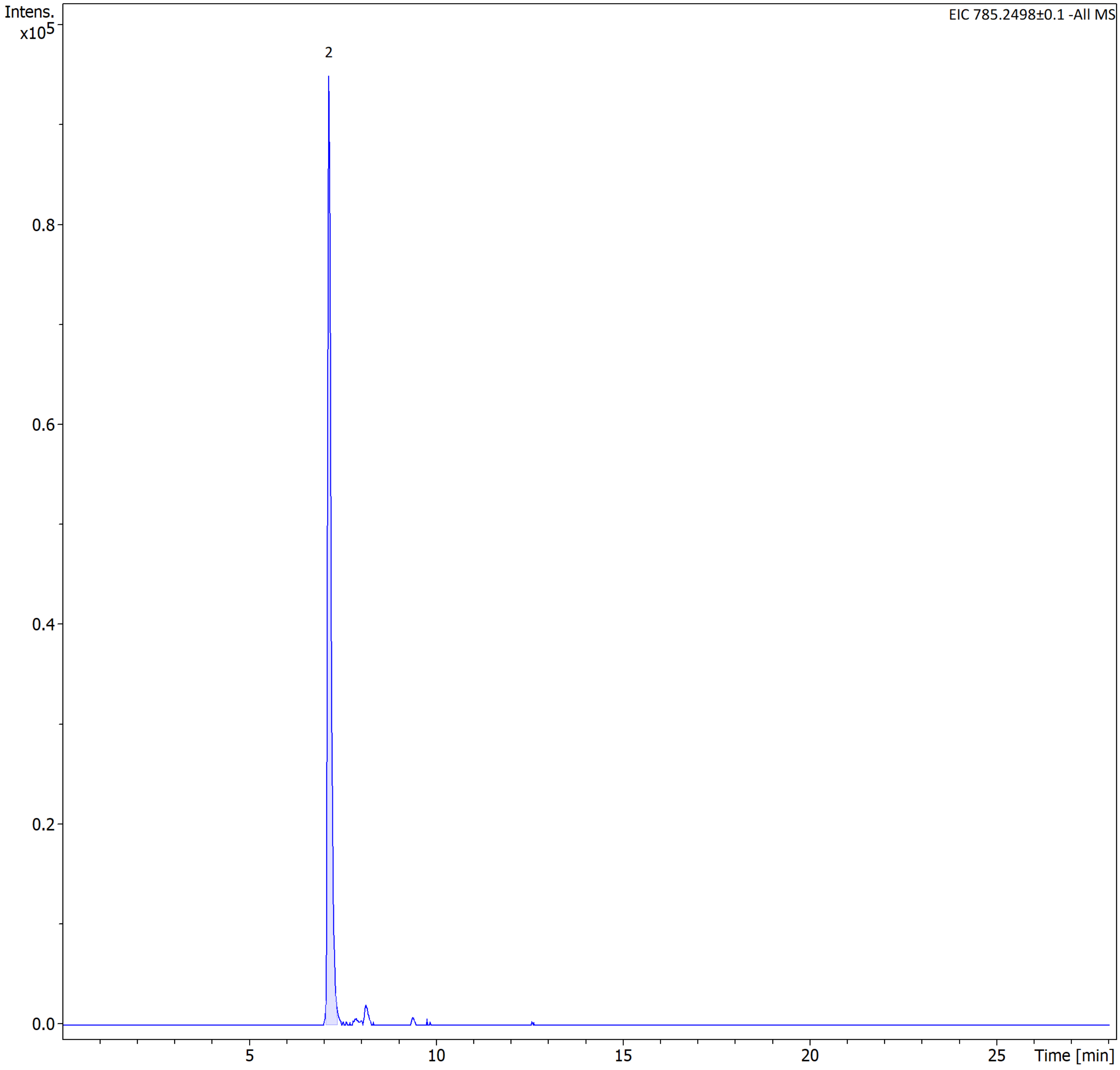

The results of the analyses conducted in our laboratory are intended solely for scientific research purposes. The laboratory is not responsible for the use of this data in legal matters.

AOE "Nazarbayev University"
Office of Research Core Facilities and HPC
Address: 53, Kabanbay Batyr ave.,
Astana, 010000, Republic of Kazakhstan

+7 (7172) 70 64 78
provost.cf@nu.edu.kz

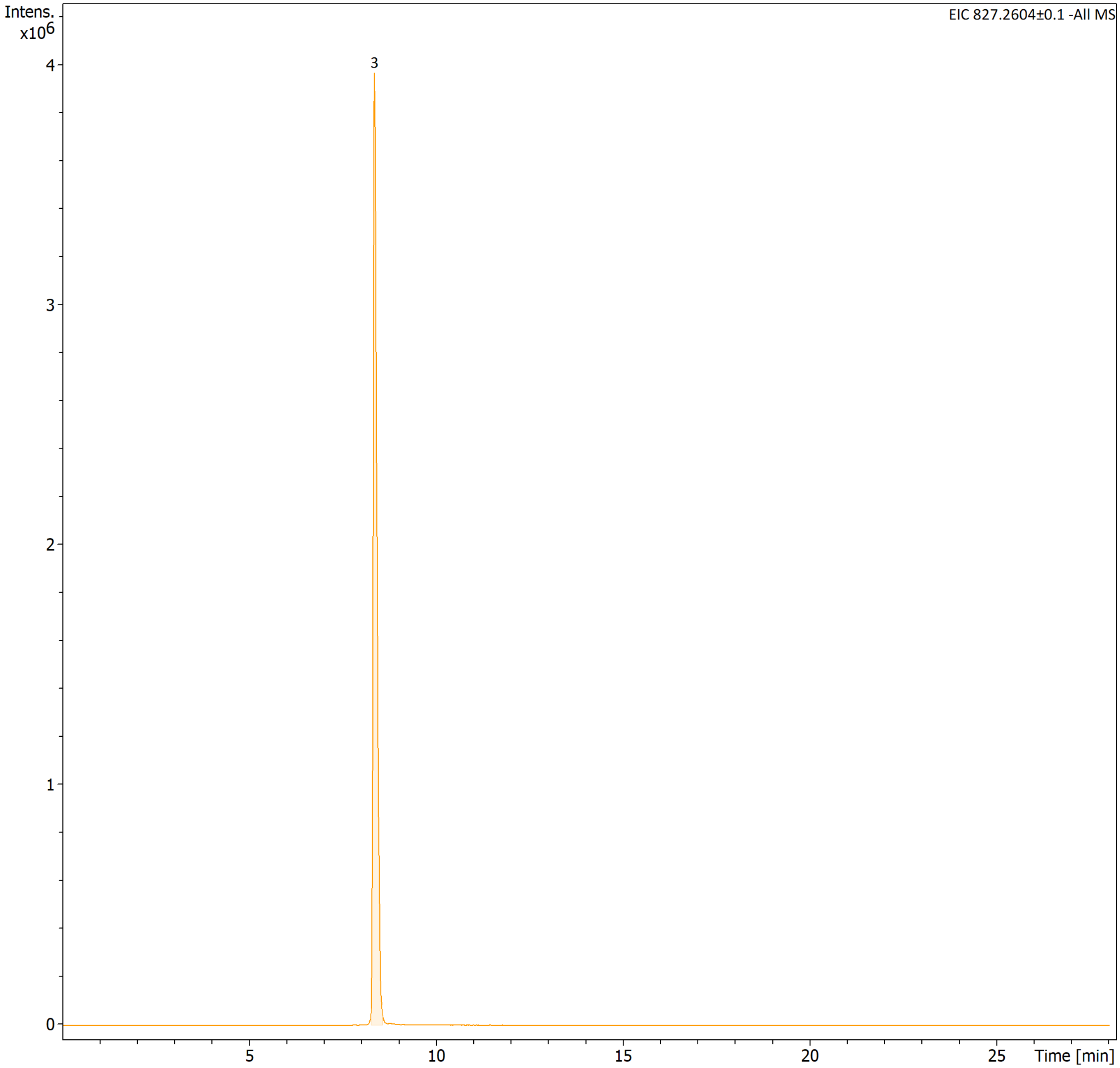

The results of the analyses conducted in our laboratory are intended solely for scientific research purposes. The laboratory is not responsible for the use of this data in legal matters.

AOE "Nazarbayev University"
Office of Research Core Facilities and HPC
Address: 53, Kabanbay Batyr ave.,
Astana, 010000, Republic of Kazakhstan

+7 (7172) 70 64 78
provost.cf@nu.edu.kz

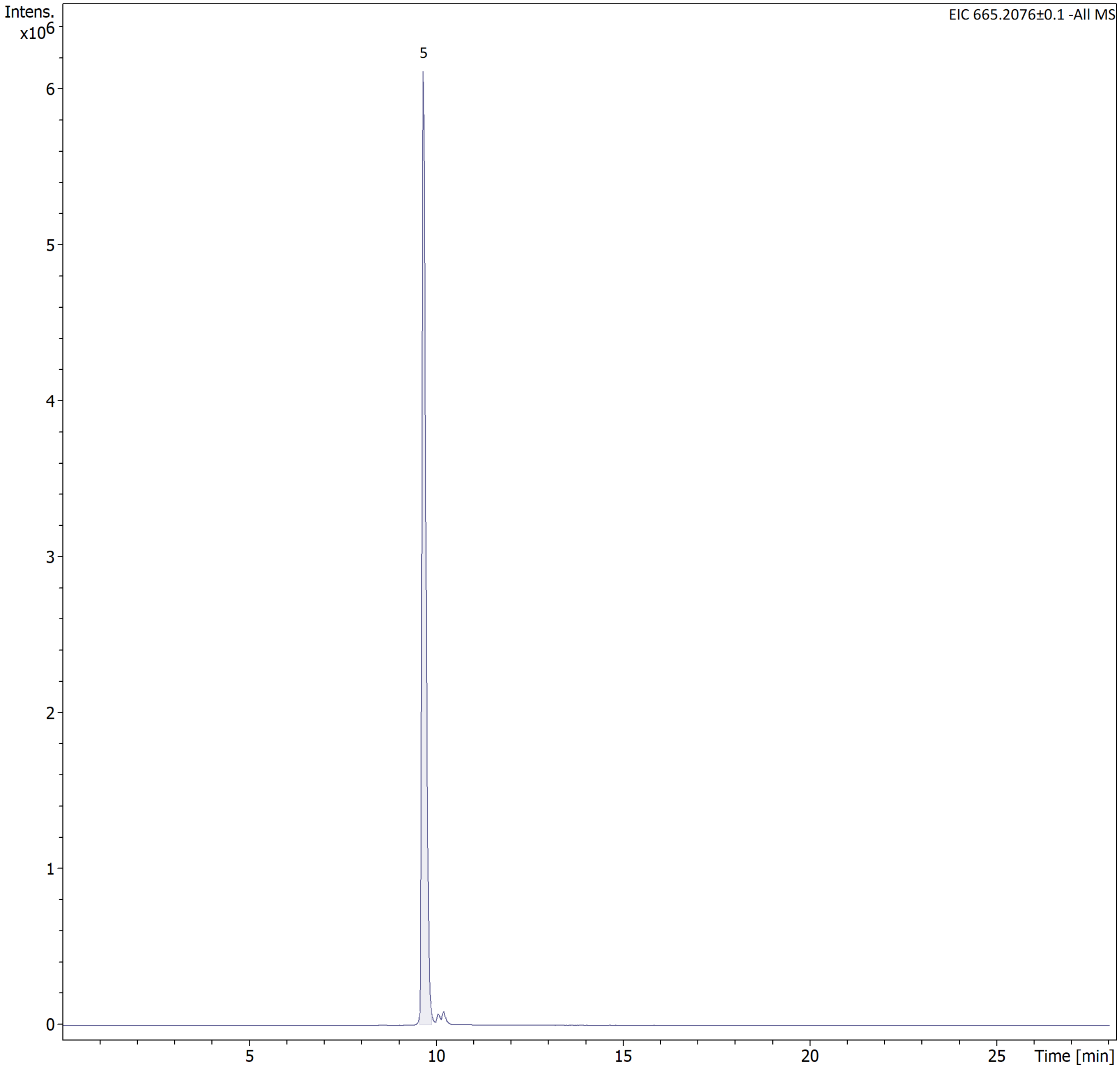

The results of the analyses conducted in our laboratory are intended solely for scientific research purposes. The laboratory is not responsible for the use of this data in legal matters.

AOE "Nazarbayev University"
Office of Research Core Facilities and HPC
Address: 53, Kabanbay Batyr ave.,
Astana, 010000, Republic of Kazakhstan

+7 (7172) 70 64 78
provost.cf@nu.edu.kz

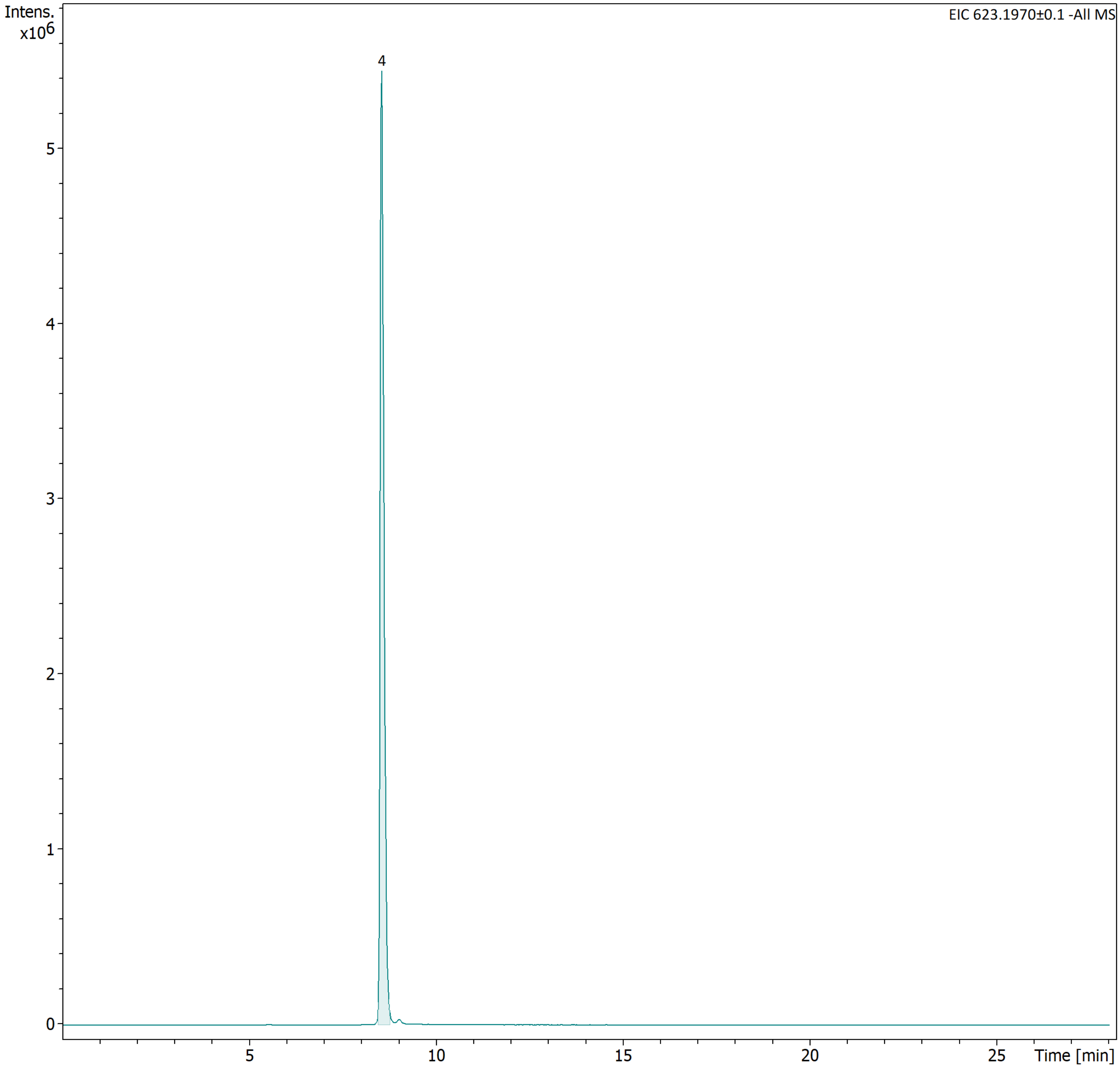

The results of the analyses conducted in our laboratory are intended solely for scientific research purposes. The laboratory is not responsible for the use of this data in legal matters.

AOE "Nazarbayev University"
Office of Research Core Facilities and HPC
Address: 53, Kabanbay Batyr ave.,
Astana, 010000, Republic of Kazakhstan

+7 (7172) 70 64 78
provost.cf@nu.edu.kz

#	Compound Label	RT [min]	Area	Int. Type	I	S/N	Trace	Max. m/z	FWHM [min]
1	Salidroside	5.5	7126789	Chromatogram	1026279	35.4	EIC 299.1125±0.1 -All MS	345.1190	0.1
2	Echinacoside	7.1	620258	Chromatogram	96257	196.5	EIC 785.2498±0.1 -All MS	785.2509	0.1
3	Tubuloside	8.4	28205370	Chromatogram	3967115	19172.5	EIC 827.2604±0.1 -All MS	827.2614	0.1
4	Verbascoside	8.5	39472084	Chromatogram	5444587	26562.7	EIC 623.1970±0.1 -All MS	623.1980	0.1
5	Acetylacteoside	9.7	45592488	Chromatogram	6167697	24674.8	EIC 665.2076±0.1 -All MS	665.2089	0.1

Salidroside

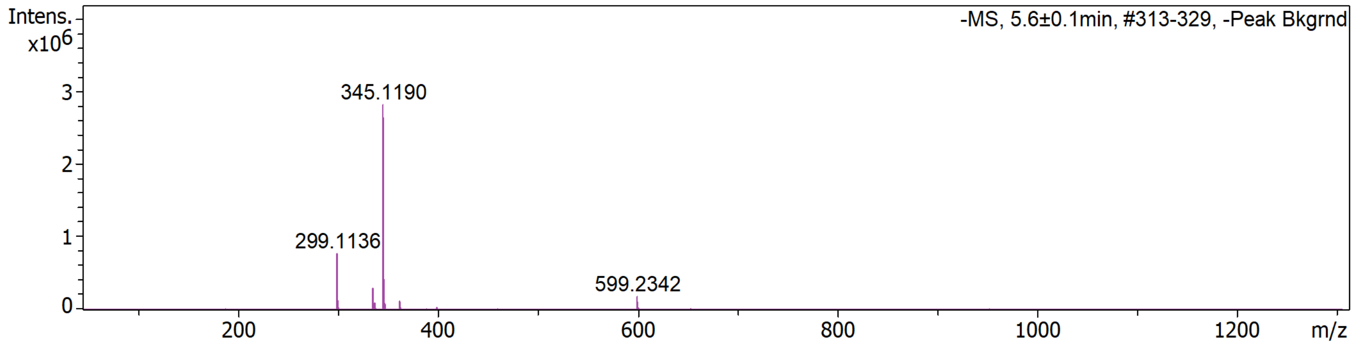

Echinacoside

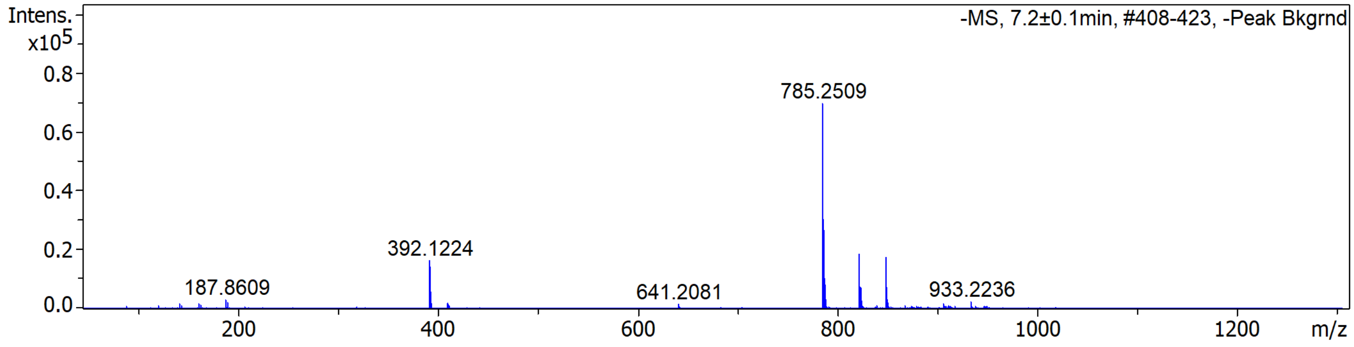

The results of the analyses conducted in our laboratory are intended solely for scientific research purposes. The laboratory is not responsible for the use of this data in legal matters.

NAZARBAYEV UNIVERSITY

AOE "Nazarbayev University"
Office of Research Core Facilities and HPC
Address: 53, Kabanbay Batyr ave.,
Astana, 010000, Republic of Kazakhstan

+7 (7172) 70 64 78
provost.cf@nu.edu.kz

Tubuloside

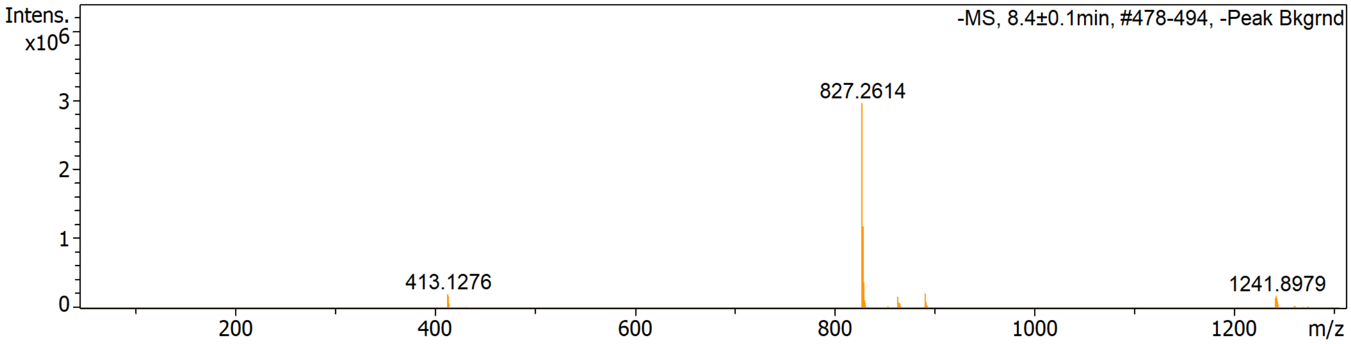

Verbascoside

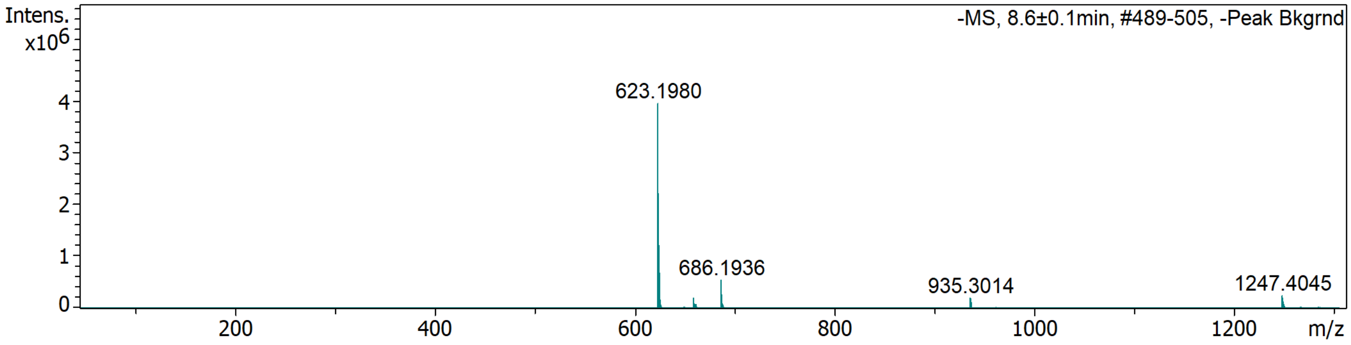

Acetylacteoside

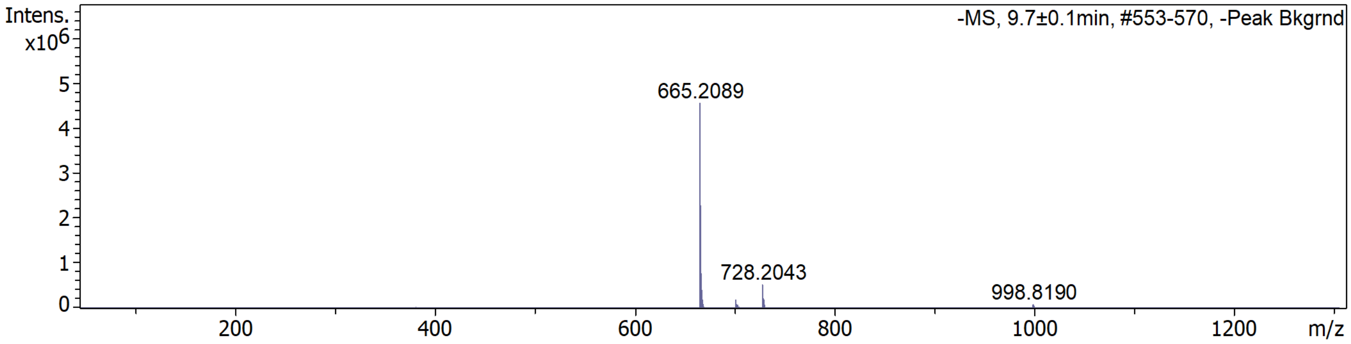

The results of the analyses conducted in our laboratory are intended solely for scientific research purposes. The laboratory is not responsible for the use of this data in legal matters.
